# Saudi Arabian evidence‐based clinical practice guideline for the management of children with autism spectrum disorder: A national guideline adaptation using the KSU‐modified‐ADAPTE methodology

**DOI:** 10.1002/gin2.70001

**Published:** 2024-11-21

**Authors:** Shuliweeh Alenezi, Fahad Bashiri, Amel Alawami, Ayman Alhazmi, Somayyah Aladamawai, Faisal Alnemary, Yasser Alqahtani, Maysaa Buraik, Saleh AlSuwailem, Shahad Akhalifah, Saleh Al‐Salehi, Yasser Amer

**Affiliations:** ^1^ Department of Psychiatry College of Medicine, King Saud University Riyadh Saudi Arabia; ^2^ Pediatrics Department, Pediatric Neurology Division College of Medicine, King Saud University Riyadh Saudi Arabia; ^3^ Johns Hopkins Aramco Healthcare Dhahran Saudi Arabia; ^4^ Developmental Pediatric Department Children's Hospital, King Saud Medical City, Ministry of Health Riyadh Saudi Arabia; ^5^ King Abdullah bin Abdulaziz University Hospital Riyadh Saudi Arabia; ^6^ College of Medicine Princess Nourah bint Abdulrahman University Riyadh Saudi Arabia; ^7^ Autism Center of Excellence Riyadh Saudi Arabia; ^8^ King Fahad Armed Forces Hospital Jeddah Saudi Arabia; ^9^ Pediatrics Department, King Khalid University Hospital King Saud University Medical City Riyadh Saudi Arabia; ^10^ Clinical Practice Guidelines and Quality Research Unit, Corporate Quality Management Department King Saud University Medical City Riyadh Saudi Arabia; ^11^ Research Chair for Evidence‐Based Health Care and Knowledge Translation Deanship of Scientific Research, King Saud University Riyadh Saudi Arabia; ^12^ Adaptation Working Group Guidelines International Network Perth Scotland; ^13^ Department of Internal Medicine Ribeirão Preto Medical School, University of São Paulo (FMRP‐USP), Ribeirão Preto São Paulo Brazil

**Keywords:** adaptation, autism spectrum disorder, eastern mediterranean region, evidence‑based medicine, mental health, practice guideline, Saudi Arabia

## Abstract

**Introduction:**

Autism spectrum disorder (ASD) is one of the national mental health priorities that has manifested a wide variability in practice in the Kingdom of Saudi Arabia (KSA). This work aimed to adapt evidence‐based clinical practice guidelines (CPGs) for ASD to synthesize the first national CPG for the management of children with ASD in KSA.

**Methods:**

The CPG adaptation group comprised multidisciplinary expert clinicians and a CPG methodologist following the KSU‑Modified‑ADAPTE methodology. The last search date for source evidence‐based guidelines was March 2022.

**Recommendations:**

Three main categories of recommendations were included: (i) prevention and early identification, coding, psychometric tools, telehealth, risk factors and referral criteria, (ii) diagnosis, differential diagnosis, investigations and family support, (iii) interventions with problem minimization and avoidance, treatment goals, physical wellbeing, nonpharmacological interventions, sensory integration, parent‐mediated interventions, cognitive behavioural therapy, pharmacological interventions, psycho‐education of the family, special cases or comorbidities, sleep management, gastrointestinal and feeding interventions, the transition of care from paediatrics to adulthood. CPG implementation tools included a baseline assessment tool, clinical scenarios, pathways, quality measures, referral forms, screening tools and useful online resources. The adapted CPG presents practical, evidence‑based guidance with implementation tools for managing children with ASD in KSA. The project illustrated the applicability of the KSU‑modified‑ADAPTE and highlighted the importance of collaboration between clinicians and methodologists for adapting national CPGs.

## Highlights

- *Guideline adaptation*: The King Saud University (KSU)‐Modified‐ADAPTE method was used to adapt international autism spectrum disorder (ASD) management guidelines to fit Saudi Arabia's healthcare needs and system, emphasizing the importance of the local context.
- *Implementation tools*: The adapted national ASD guideline included practical tools like decision aids, educational resources, clinical pathways, performance measures, team qualifications, screening tools, online platforms and patient information tools. These aimed to improve adherence and clinical practice.
- *Facilitators and barriers*: During the adaptation, various challenges and solutions specific to Saudi Arabian healthcare were identified to enhance guideline effectiveness.
- *Research needs*: Identified gaps in research will guide future studies to support updates and ensure the guideline's relevance and impact.

## RECOMMENDATIONS SUMMARY

**DIAGNOSIS**

**Referral for diagnostic clarification**

▪ It is recommended that the healthcare provider *must* receive formal professional training in typical child development and the signs and/or symptoms of common neurodevelopmental and behavioral conditions, including those associated with autism‐spectrum‐disorder (ASD), as well as common co‐occurring and differential diagnosis conditions.
▪ Healthcare professionals *must* have a good understanding of the different forms of expression of ASD symptomatology across developmental stages and the symptomatology of common coexisting and alternative conditions.

**Assessment setting**

▪ It is recommended that a clinic setting *should* be considered an appropriate, but not essential, venue for an assessment of ASD concerns.
▪ It is recommended that information about an individual's presentation in all community settings relevant to their daily life *must* be collected.
▪ It is recommended that information about an individual's presentation in community settings *must* be obtained by one or more members of the Assessment Team.
▪ It is recommended that functional assessments *must* take place in a setting where the client feels comfortable and confident to discuss their level of functioning and support needs.

**Diagnostic criteria**

▪ All professionals involved in diagnosing ASD in children, young people or adults *should* consider using the current version of either ICD‐10‐AM or DSM‐5. The classification system used for diagnosis *should* be recorded in the patient's notes.
▪ The autism spectrum disorder multidisciplinary team (ASD‐MDT) *should* have the skills and competencies to: (i) carry out an autism diagnostic assessment, and (ii) communicate with children and young people with suspected or known autism, and with their parents and carers, and sensitively share the diagnosis with them.
▪ Single clinician diagnostic evaluation *must* include a medical practitioner who holds specialist registration in Kingdom of Saudi Arabia (KSA) in the field of community child health, general pediatrics, child and adolescent psychiatry & pediatric neurology, or a medical practitioner (family medicine, pediatrician) who holds general or specialist registration in KSA with at least 6 years of relevant experience, training, or supervision in the assessment of neurodevelopmental disorders plus a certified occupational therapist, clinical neuropsychologist, or pediatric speech pathologist.

**Timing of diagnosis**

▪ Regardless of the findings of any earlier assessments, referral for further assessment for ASD *should* be considered at any age.

**Differential diagnosis**

▪ It is recommended that, at each stage of the Diagnostic Evaluation, the clinicians *must* collect and evaluate information to consider the full range of clinical explanations for the presentation of signs and/or symptoms and test these possible explanations against the evidence for an ASD diagnosis in the context of other differential and co‐occurring diagnoses.
▪ Clinicians without the clinical qualifications or expertise to adequately evaluate potential differential diagnoses for a given individual *should not* undertake the assessment of ASD concerns.
▪ Healthcare professionals *should* recognize that children and young people with ASD may also have additional developmental disorders, medical problems or emotional difficulties/disorders and should have access to the same range of therapeutic interventions as any other child.
▪ *Do not* routinely perform any medical investigations as part of an autism diagnostic assessment.

**INTERVENTIONS**

**Nonpharmacological interventions**

▪ Treatment plans *should* be comprehensive, and include behavioral needs, educational interventions, psychosocial treatments, communication, environmental and systems issues and the suitability (or not) of medication.
▪ The intervention *should* be delivered by a trained professional. For preschool children consider parent, carer, or teacher mediation. For school‐aged children consider peer medication.

**Pharmacological interventions**

▪ Patients and their carers *should* be advised of potential side effects before treatment is started.

**Psychopharmacology adverse effects**

▪ Full understanding and agreement *should* be documented in the medical records.

**Psychoeducation for the family**

▪ Education and skills interventions *should* be offered to parents of all children and young people diagnosed with ASD.

**Sleep management**

▪ Behavioral therapy *should* be considered for children and young people with ASD who experience sleep problems.
▪ Children with ASD who present with signs of possible obstructive sleep apnea, or sleep‐disordered breathing (loud snoring, choking or periodic stopping of breathing during sleep) *should* be referred to sleep medicine services or ENT for assessment.
▪ In children with ASD who have sleep difficulties which have not resolved following behavioral interventions, a trial of melatonin to improve sleep onset *should* be considered.

**Gastrointestinal and feeding interventions**

▪ Gastrointestinal symptoms in children and young people with ASD *should* be managed in the same way as in children and young people without ASD.

**Transition of care from pediatrics to adulthood**

▪ Local autism teams *should* ensure that young people with autism who are receiving treatment and care from pediatric services are reassessed at around 14 years to establish the need for continuing treatment into adulthood.
▪ The timing of transition may vary locally and individually but *should* usually be completed by the time the young person is 18 years. Variations should be agreed on by both child and adult services.
▪ As part of the preparation for the transition to adult services, health and social care professionals *should* carry out a comprehensive assessment of the young person with autism.

## 1 INTRODUCTION AND SCOPE

Autism spectrum disorder (ASD) is a neurodevelopmental disorder characterized by challenges in communication, social interaction and repetitive behaviours. Its origins are complex, attributed to genetic, environmental and biological factors.^1^, ^2^ Worldwide, approximately 1 in 100 children are diagnosed with ASD, and this trend is mirrored in the Kingdom of Saudi Arabia (KSA), which has seen a steady increase in ASD diagnoses over the years.^3^, ^4^

The repercussions of ASD are profound, affecting not only the diagnosed individuals but also their families and the broader community. The societal and economic impact includes direct medical expenses, costs related to education and lost productivity.^5^ Moreover, given the chronic nature of ASD, affected individuals often need continuous, specialized support throughout their lives.^6^

In Saudi Arabia, there is a heightened focus on ASD due to its increasing prevalence and the evident need for improved diagnostic and support services. While progress has been made recently, there is an ongoing demand for more research, enhanced public awareness and specialized training for healthcare professionals tailored to the challenges presented by ASD in the Saudi context.^7^

However, KSA currently lacks standardized national guidelines for ASD, which are essential for providing consistent and effective care.^8^ These guidelines, when tailored to KSA's unique sociocultural environment, would aid in early diagnosis and interventions, ensuring healthcare providers have access to the latest best practices.^9^ The emphasis on cultural sensitivity is pivotal for better acceptance and adherence, resulting in improved outcomes for ASD individuals and their families.^7^

The Clinical Practice Guideline Adaptation methodology provides a structured process to adapt guidelines from one cultural or organizational environment to another. It is a practical alternative to creating new guidelines from the ground up.^10^, ^11^, ^12^, ^13^ The development of such national guidelines is aligned with the overarching goals of the 2030 Vision Health Sector Transformation Strategy in KSA.^14^

At the onset of this project, KSA lacked an official national guideline for the management of ASD in children. This study, championed by the National Center for Developmental Behavioural Disorders (NCDBD‐SHC) and endorsed by the National Center for Evidence‐Based Medicine (NCEBM‐SHC), sought to adapt comprehensive, evidence‐based guidelines for ASD care to KSA's specific healthcare landscape. This endeavour used the structured and evidence‐informed CPG adaptation approach known as the King Saud University modified ADAPTE (or the KSU‐Modified‐ADAPTE) method.^12^, ^13^

The objective of this adapted clinical practice guideline (CPG) is to provide evidence‐based recommendations for management including screening, surveillance, diagnosis, assessment and clinical and therapeutic interventions for children with ASD. It aims to decrease morbidity and mortality and to improve the experience in general for children with ASD and those who care for them.

## 2 METHODS

This CPGA project followed the KSU Modified ADAPTE Methodology that has been used earlier in several local and national CPG projects and included the three phases of CPGA (set‑up, adaptation and finalization) (Table 1).^13^, ^14^, ^15^

**Table 1:** The KSU‐modified‐ADAPTE CPGA methodology.^a^

Phase	Steps/tools
PHASE 1: SET‐UP	1. Check whether adaptation is feasible
	Tool 1: Clinical practice guideline (CPG) development and implementation resources
	Tool 2: Search sources and strategies (modified)
	2. Establish an organizing committee/CPG adaptation group
	3. Select a high‐priority CPG topic
	4. Identify necessary resources and skills
	5. Complete tasks for the set‐up phase (including registering the protocol of the SR of CPGs and the CPG project, and identifying all stakeholders)
	Tool 3: Declaration of the conflict of interests
	Tool 4: Consensus process resources
	6. Write adaptation plan
	Tool 5: Adaptation working plan template
PHASE 2: ADAPTATION	7. Determine the health questions (PIPOH)
	Tool 6: PIPOH model (modified) template
	8. Search for SOURCE CPGs and other relevant documents
	Tool 2: Search sources and strategies
	Tool 7: Table for summarizing CPG characteristics.
	(New) Tool: Checklist for inclusion/exclusion criteria
	9. Screen retrieved SOURCE CPGs
	Tool 8: Table for summarizing CPG content
	10. Reduce a large number of retrieved SOURCE CPGs
	Tool 9: The AGREE II Instrument (or domain 3 only)
	11. Assess CPG quality
	Tool 9: The AGREE II instrument (updated)
	Tool 10: AGREE inter‐rater agreement spreadsheet and AGREE II score calculation spreadsheet
	12. Assess CPG currency
	Tool 11: Currency survey of CPG developers
	13. Assess CPG content
	Tool 12: Sample recommendation matrix
	14. Assess CPG consistency *(The group did not use this step and relied on the standardized of AGREE II Domain 3 scores of the included appraised source CPGs)*
	Tool 13: Evaluation sheet search and selection of evidence
	Tool 14: Evaluation sheet‐scientific validity of CPGs (consistency recommendations)
	15. Assess acceptability and applicability *(the group did not use this step and relied on the standardized scores of AGREE II domains 2 and 5 scores of the included appraised source CPGs)*
	Tool 15: Evaluation sheet‐acceptability/applicability
	16. Review assessments (new) Tool
	17. Select between CPGs and recommendations to create an adapted CPG
	18. Prepare draft adapted CPG
	Tool 16: Checklist of adapted CPG content (the group was guided by the items of the RIGHT‐Ad@pt Checklist and the AGREE Reporting Checklist)
PHASE 3: FINALIZATION	19. External review—target audience of the CPG
	Tool 17: External clinical content and methodology review templates (modified)
	20. Consult with endorsement bodies
	21. Consult with source CPG developers
	22. Acknowledge source documents
	23. Plan for the aftercare of the adapted CPG
	Tool 18: Table for reporting on results of the update process (the future update to be guided by the CheckUp Tool)
	24. Produce final guidance document and implementation tools

a References: Fervers et al.,^16^ Amer et al.,^13^ Amer et al.,^12^ Brouwers et al.,^17^ Song et al.^18^

Moreover, we have used the AGREE Reporting Checklist and the RIGHT Statement extension for reporting adapted CPGs (RIGHT‐Ad@pt Checklist) to report this CPG^18^, ^19^ (Supporting Information S1: File S1).

### 2.1 Guideline registration

The CPGA project was registered in two international CPG registries:

1. The Practice guideline REgistration for transPAREncy (PREPARE) platform (formerly named the International Practice Guideline Registry Platform (IPGRP)). Registration Number: IPGRP‐2022CN002 http://www.guidelines-registry.org/guid/1385.
2. International Guidelines Library and Registry, Guidelines International Network (GIN) https://guidelines.ebmportal.com/node/70209 (through the NCEBM‐SHC, a GIN organizational member).

### 2.2 The KSU modified ADAPTE methodology

#### 2.2.1 Phase one: Set‐up

##### Selecting and prioritizing the CPG health topic and checking the feasibility of adaptation

The NCDBD‐SHC identified ASD as the high‐priority health topic for this national CPGA project. A multidisciplinary guideline adaptation group (GAG) was gathered from all relevant clinical and academic stakeholders that were identified by the project lead/clinical chair and methodology chair. A preliminary rapid search revealed many published ASD CPGs.

##### Establishing a GAG

The NCDBD‐SHC assigned the project lead and clinical chair (S. A.), who worked closely with the methodology chair (Y. S. A.) to define the initial scope and required stakeholders for the GAG.

The GAG clinical panel members included two child and adolescent psychiatrists, a paediatric neurologist, three developmental‐behavioural paediatricians, a behaviour analyst, a clinical psychologist, a speech and language therapist and an occupational therapist who represent different healthcare sectors in KSA.

##### Identifying necessary resources and skills

The methodology chair conducted capacity‐building sessions for the GAG members on the CPGA methodology and systematic review and quality appraisal of CPGs at the outset of the project, in addition to hands‐on technical support throughout the process.^17^, ^20^, ^21^, ^22^, ^23^

##### Completing the tasks of the set‐up phase

All members of the GAG declared their conflicts of interest (COIs) at the outset of the CPG project. No competing interests were identified based on the nine core principles for managing COIs that were recommended by the Guidelines International Network.^24^ The COI forms were documented by all contributors using Google Forms (Supporting Information S1: File S1).

##### Writing up the CPGA working plan

A timeline for the CPGA project was outlined by the NCDBD‐SHC, finalized, and agreed upon by all GAG members. The project started on 28 April 2021 and was completed on 30 December 2021. Secretarial support was provided by the NCDBD‐SHC to organize and facilitate 13 2‐h meetings for the larger groups that included two face‐to‐face and 11 online meetings. Moreover, 10 parallel meetings were conducted by subgroups of the GAG to complete specific tasks within the CPGA process (e.g., searching for source CPGs, screening source CPGs, quality assessment of CPGs, drafting the first version of the adapted recommendations, identifying and drafting the CPG implementation tools, and drafting the final version of the adapted CPG based on the external review input).

#### 2.2.2 Phase two (adaptation)

##### Determining the health questions, searching, screening of source CPGs and relevant documents and reducing the large number of retrieved CPGs

The PIPOH (viz. patient population, interventions, professionals, outcomes and healthcare context) Model was used to identify the health questions that guided the search and screening process (Table 2). A comprehensive systematic review of relevant Source CPGs was conducted as part of this project and reported separately.^21^ Eligible CPGs included evidence‐based CPGs with a properly documented development methodology (rather than expert or consensus‐based CPGs), CPG development groups (rather than single‐authored CPGs), international and national CPGs, CPGs published after October 2015, recommendations in Arabic or English only (other languages were excluded), and original de novo developed source CPGs (rather than adapted CPGs).^21^ The last search date for source evidence‐based guidelines was March 2022.

**Table 2:** Health or clinical questions (PIPOH).

Patient (target population)	Children with autism spectrum disorders (ASD), both genders, age group from 2 to 18 years. Common comorbidities: ADHD, anxiety and other relevant medical problems.
Interventions, practices considered, CPG category	CPG category: Management
	*Key priorities*
	*DIAGNOSIS*
	1. What is the best practice in the prevention and early identification of ASD in children to decrease morbidity and mortality for professionals?
	2. What is the best practice in assessment setting, diagnostic criteria and differential diagnosis of ASD in children to decrease morbidity and mortality for professionals?
	3. What is the best practice of the multidisciplinary team versus the single clinician for the assessment of children suspected of ASD to support early diagnosis and decrease morbidity and mortality for professionals?
	4. What is the best practice in disclosing the results of the diagnostic assessment and providing information and support for parents, families and carers of children diagnosed with ASD?
	*INTERVENTIONS*
	5. What is the best practice in providing nonpharmacological interventions (i.e., care plan, ASD multidisciplinary team, functional assessment of behaviour, psychosocial interventions for behaviour, developmental interventions, social communication skills, intensive behavioural interventions, education, support, and transition, occupational therapy, parent training and coaching for occupational therapy, sensory integration, parent‐mediated interventions and cognitive behavioural therapy) for children with ASD to decrease morbidity and mortality for professionals?
	6. What is the best practice in providing pharmacological interventions (i.e., antipsychotics, ADHD medications and psychopharmacology adverse effects) for children with ASD to decrease morbidity and mortality for professionals?
	7. What is the best practice in providing psychoeducation for the parents, families and carers of children with ASD to decrease morbidity and mortality?
	8. What are the special cases or comorbidities that need special attention in children with ASD?
	9. What is the best practice in sleep management and sleep medications for children with ASD to decrease morbidity and mortality for professionals?
	10. What is the best practice in gastrointestinal and feeding interventions for children with ASD to decrease morbidity and mortality for professionals?
	11. What is the best practice in the transition of care from paediatric to adult healthcare services for children with ASD to decrease morbidity and mortality for professionals?
	12. What are the best practice recommendations for living in the community, recreation and leisure for children with ASD?
	13. What are the recommendations for future research and health policymakers to improve the quality and safety of healthcare services for children with ASD?
Professionals (i.e., intended or target users or stakeholders)	Physicians, pharmacists, nurses, therapists, technicians.
Specific clinical specialties*	Physicians, including psychiatrists, paediatricians (behavioural developmental and paediatric neurology), medical geneticists, physical medicine and rehabilitation physicians and mental health specialists.
	Clinical Pharmacists, nurses, occupational therapists, psychologists, social workers, speech pathologists and patient/family representatives.
	*Other professionals who are not part of the main diagnostic assessment team could be included if indicated (these mentioned in detail within the CPG recommendations).
Outcomes (high priority)	Decrease death (mortality).
	Decrease major neurodevelopmental disability in surviving children (Morbidity).
Healthcare settings	Primary, secondary and tertiary neonatal healthcare services mainly nurseries, NICUs and outpatient clinics in the Kingdom of Saudi Arabia.

##### Quality appraisal of the included CPGs using the AGREE II instrument

Four eligible source ASD CPGs were critically appraised using the Appraisal of Guidelines for REsearch and Evaluation II (AGREE II) Instrument that is well known as the gold standard for CPG quality appraisal.^21^ The four national ASD CPGs were developed by The Australian Autism Cooperative Research Centre (ACRC), The New Zealand Ministry of Health (NZ‐MOH), The National Institute for Health and Care Excellence (NICE) and The Scottish Intercollegiate Guidelines Network, Healthcare Improvement Scotland (SIGN‐HIS).^21^, ^25^, ^26^, ^27^, ^28^ The findings of that systematic review, quality assessment, and analysis of the four CPGs revealed them to be of high quality, evidence‐based in compliance with the international standards of trustworthy CPGs, and potentially applicable and acceptable to the local KSA healthcare system and services provided to children with ASD.^21^

Moreover, the standardized score of the AGREE II third domain of rigour of development was 84%, 93%, 86% and 85% for the four appraised CPGs ACRC, NICE, NZ‐MOH and SIGN‐HIS, respectively.^21^ The four CPGs clearly reported the evidence base of their clinical recommendations. NICE and SIGN‐HIS CPGs documented and reported their Grading of Recommendations: Assessment, Development and Evaluation (GRADE) Evidence Tables that were reviewed and accepted by the GAG. Accordingly, the GAG did not opt to develop new GRADE Evidence‐to‐Decision Frameworks (EtDs).^29^

##### Assessing the currency of the four selected source CPGs

At the end of this phase, the GAG thoroughly studied the four source CPGs and updated the search to confirm that there was no new evidence relevant to these CPGs that could possibly invalidate any of their recommendations, no plans to update the CPGs in the near future, and the four CPGs were last updated or revalidated in August 2016 (NZ‐MOH), December 2017 (NICE), October 2018 (AACRC) and August 2019 (SIGN‐HIS).

##### Methods for formulating recommendations (decision‐making process)

After a thorough review, comparison and several focus‐group discussions of the sets of recommendations and implementation tools presented by the four high‐quality CPGs, the GAG decided to accept, adopt and adapt their recommendations and linked evidence with practical contextualization related to the KSA national health system and the actual local organizations involved in different ASD care services. The first draft of the KSA‐adapted ASD CPG with its set of recommendations and CPG Implementation (CPGI) tools was prepared.

Each member of the GAG was tasked with reviewing specific sections related to the diagnosis or intervention recommendations for ASD across four CPGs. Their role involved providing initial feedback on the applicability and acceptability of these recommendations within the healthcare system of KSA, as well as presenting their findings to the GAG for further discussion and consensus‐building. Through this collaborative process, a total of 16 new good practice points (GPPs) were developed, drawing upon the clinical expertise and healthcare insights of the GAG members. Additionally, three GPPs were adopted from the GPPs outlined in the SIGN‐HS CPGs.

The GAG conducted an informal consensus process after reviewing the recommendations of the four source high‐quality CPGs in consideration for their applicability, acceptability and implementability in KSA and in line with its national health system.

#### 2.2.3 Phase three (finalization)

##### External review (clinical content and methodology)

The first draft of the adapted ASD CPG draft was sent to a national external review group (ERG) representing different healthcare sectors in KSA. This ERG conducted clinical content and methodology reviews. The ERG clinical reviewers included a consultant psychoanalyst, a child and adolescent psychiatrist, a consultant developmental and behavioural paediatrician, a consultant clinical neuropsychologist, a consultant occupational therapist, a consultant speech and language pathologist, a senior nurse academic, a senior clinical psychiatric pharmacist, and a medical technologist and ASD patient representative and advocate. Additionally, a senior expert guideline methodologist and academic was invited to review the CPGA methodology. The GAG reviewed the feedback received from the ERG and addressed their comments and suggestions in the final draft of the CPG. For detailed recommendations and implementation tools, please refer to Supporting Information S1: File S1.

##### Consulting with endorsement bodies and the source CPG developer(s)

The clinical chair of the GAG (S. A.) contacted the relevant contact persons of the developer organizations of the four source CPGs that have granted us official permission to use their CPGs for our KSA ASD CPGA (reported in the full CPG document) (Supporting Information S1: File S1).

The finalized copy of the KSA‐adapted ASD CPG was reviewed, approved and endorsed by the NCDBD‐SHC and afterwards submitted to the NCEBM‐SHC where it was reviewed and discussed by its scientific committee, appraised by the AGREE II Instrument, and finally was approved by the centre as a national CPG in the KSA with the addition of its “SAEEB” official seal on the CPG's cover page after the GAG addressed all of its scientific committee's comments like including only medications that are approved by the Saudi Food and Drug Authority.

##### Acknowledging the source documents

The four source CPGs' articles, documents and websites have been transparently acknowledged in the final adapted CPG full document after being granted their permissions.

##### Planning for aftercare of the adapted CPG: Future review and update

The GAG has recommended a planned review and update of this adapted CPG using the AGREE Enterprise's Checklist for the Reporting of Updated Guidelines (CheckUp) Tool to be after 2 years from its publication and release in 2023 (scheduled in 2026) or earlier, if any potentially consequential update was published either in the source CPGs or in their relevant evidence.^30^, ^31^ Moreover, the results of national and/or local regular audit and feedback activities during CPGI should inform and contribute to the regular updates of this CPG.

##### Producing the final adapted CPG document

An electronic copy of the final adapted CPG full document can be made available from the official website of the NCDBD‐SHC. Table 3 presents the full map of the CPGA process, including the steps that were followed and the steps that were not followed with the relevant reasons.

**Table 3:** Decision support for the autism‐spectrum‐disorder (ASD) guideline adaptation group (GAG).

Phase	Module	Step	Tool	Decision		Reason *(if not utilized)*
				Utilized	Not utilized	
ONE: SET‐UP	1.1 Preparation	1	1	√		
			2	√		
		2		√		
		3		√		
		4		√		
		5	3	√		
			4	√		
			1	√		
		6	5	√		
TWO: ADAPTATION	2.1. Scope and purpose	7	6	√		
	2.2. Search and screen	8	2	√		
			7	√		
		9	8	√		
		10	9		√	Decided to rely on inclusion/exclusion criteria (filters) and PIPOH (viz. patient population, interventions, professionals, outcomes and healthcare context) compatibility
			10		√	
	2.3. Assessment	11	9	√		
			10	√		
		12	11	√		
		13	12		√	The GAG decided to select the relevant recommendations from the four clinical practice guidelines (CPGs): AACRC 2018, MOH NZ 2016, NICE 2017 and SIGN‐HIS 2016 to the Saudi Arabian Healthcare context
		14	13		√	The GAG decided to rely on the standardized Scores of AGREE II domain 3 (Rigour of development)
			14		√	
		15	15		√	Decided to rely on the standardized scores of AGREE II Domains 2 (Stakeholder Involvement) and 5 (applicability)
	2.4. Decision and selection	16	Table 3	√		
		17	Decision‐making and selection	√		The GAG modified the options to be two (Accept or Reject) rather than five according to the KSU‐Modified‐ADAPTE methodology
	2.5. Customization	18	16	√		
THREE: FINALIZATION	3.1. External review and acknowledgement module	19	17	√		
		20		√		
		21		√		
		22		√		
	3.2. Aftercare planning	23	18	√		
	3.3. Final production	24		√		

The adaptation of recommendations of the four source CPGs was conducted as related to what is applicable to the KSA health system and the ASD services currently provided, planned, or under development (e.g., the current transformation of healthcare, the new model of care, and the new standards of the Saudi Central Board for Accreditation of Healthcare Institutions (CBAHI) Autism service certification programme). Two main factors facilitated the adoption and adaptation of the recommendations. First, the health systems of the developer countries of the four national CPGs included systems of National Health Service (UK) and National Health Insurance (Australia and New Zealand) that are both applicable to the transforming KSA national health system as the government provides most healthcare services through public healthcare facilities and/or insurance. Second, KSA is classified by the World Bank as a high‐income country like the United Kingdom, Australia and New Zealand.^32^, ^33^

We could not recruit a health economist either for the GAG nor for the ERG and henceforth we did not conduct Health Technology Assessment(s) during this CPGA project.

Benefits and harms were considered for each adopted or adapted recommendation and were assessed as part of the AGREE II appraisal for the four source CPGs.

The potential values and preferences of children with ASD, their families, and carers were discussed during the external review process through the participation of a patient/family representative and from the input of the multidisciplinary ASD healthcare providers representing different healthcare sectors and services in KSA. This discussion was reflected in the finalized adapted recommendations and CPGI tools that included a patient and family health educational guide in standard Arabic.

## 3 RECOMMENDATIONS

### 3.1 Diagnosis

#### 3.1.1 Referral for diagnostic clarification

▪ It is recommended that the healthcare provider *must* receive formal professional training in typical child development and the signs and/or symptoms of common neurodevelopmental and behavioural conditions, including those associated with ASD, as well as common co‐occurring and differential diagnosis conditions (Adapted from ACRC). *Rationale:* We have chosen consistent terminology for our CPGs, ensuring alignment with the must/should system while maintaining the intended recommendation strength.
▪ Healthcare professionals *must* have a good understanding of the different forms of expression of ASD symptomatology across developmental stages and the symptomatology of common coexisting and alternative conditions (Adopted from NZ MOH).

#### 3.1.2 Assessment setting

▪ It is recommended that a clinic setting *should* be considered an appropriate, but not essential, venue for an assessment of ASD concerns (Adapted from ACRC). *Rationale:* We have chosen consistent terminology for our CPGs, ensuring alignment with the must/should system while maintaining the intended recommendation strength.
▪ It is recommended that information about an individual's presentation in all community settings relevant to their daily life *must* be collected (Adapted from ACRC). *Rationale:* We have chosen consistent terminology for our CPGs, ensuring alignment with the must/should system while maintaining the intended recommendation strength.
▪ It is recommended that information about an individual's presentation in community settings *must* be obtained by one or more members of the Assessment Team (Adapted from ACRC). *Rationale:* We have chosen consistent terminology for our CPGs, ensuring alignment with the must/should system while maintaining the intended recommendation strength.
▪ It is recommended that functional assessments *must* take place in a setting where the client feels comfortable and confident to discuss their level of functioning and support needs (adapted from ACRC). *Rationale:* We have chosen consistent terminology for our CPGs, ensuring alignment with the must/should system while maintaining the intended recommendation strength.

#### 3.1.3 Diagnostic criteria

▪ All professionals involved in diagnosing ASD in children, young people or adults *should* consider using the current version of either ICD‐10‐AM or DSM‐5. The classification system used for diagnosis *should* be recorded in the patient's notes (Adapted from SIGN). *Rationale:* We only specified the ICD‐10‐AM version as it is the official diagnostic classification system used by the Ministry of Health and the National Health Information Center, Saudi Health Council in Saudi Arabia.
▪ The ASD multidisciplinary team (ASD‐MDT) *should* have the skills and competencies to (i) carry out an autism diagnostic assessment, and (ii) communicate with children and young people with suspected or known autism, and with their parents and carers, and sensitively share the diagnosis with them (Adapted from NICE). *Rationale:* We decided to unify the term for the autism team to be: The ASD‐MDT.
▪ Single clinician diagnostic evaluation *must* include a medical practitioner who holds specialist registration in KSA in the field of community child health, general paediatrics, child and adolescent psychiatry and paediatric neurology, or a medical practitioner (family medicine, paediatrician) who holds general or specialist registration in KSA with at least 6 years of relevant experience, training, or supervision in the assessment of neurodevelopmental disorders plus a certified occupational therapist, clinical neuropsychologist, or paediatric speech pathologist (Adapted from ACRC). *Rationale:* We have chosen consistent terminology for our CPGs, ensuring alignment with the must/should system while maintaining the intended recommendation strength. Additionally, we specified Kingdome of Saudi Arabia (KSA) registration to reflect the local context.

#### 3.1.4 Timing of diagnosis

▪ Regardless of the findings of any earlier assessments, referral for further assessment for ASD *should* be considered at any age (Adopted from SIGN).

#### 3.1.5 Differential diagnosis

▪ It is recommended that, at each stage of the diagnostic evaluation, the clinicians *must* collect and evaluate information to consider the full range of clinical explanations for the presentation of signs and/or symptoms and test these possible explanations against the evidence for an ASD diagnosis in the context of other differential and co‐occurring diagnoses (Adapted from ACRC). *Rationale:* We have chosen consistent terminology for our adapted CPG, ensuring alignment with the must/should system while maintaining the intended recommendation strength.
▪ Clinicians without the clinical qualifications or expertise to adequately evaluate potential differential diagnoses for a given individual *should not* undertake the assessment of ASD concerns (Adopted from ACRC).
▪ Healthcare professionals *should* recognize that children and young people with ASD may also have additional developmental disorders, medical problems or emotional difficulties/disorders and should have access to the same range of therapeutic interventions as any other child (Adopted from SIGN).
▪ *Do not* routinely perform any medical investigations as part of an autism diagnostic assessment (Adopted from NICE).

### 3.2 Interventions

#### 3.2.1 Nonpharmacological interventions

▪ Treatment plans *should* be comprehensive and include behavioural needs, educational interventions, psychosocial treatments, communication, environmental and systems issues and the suitability (or not) of medication (Adopted from NZ MOH).
▪ The intervention *should* be delivered by a trained professional. For preschool children, consider parent, carer, or teacher mediation. For school‐aged children, consider peer medication (Adopted from NICE).

#### 3.2.2 Pharmacological interventions

▪ Patients and their carers *should* be advised of potential side effects before treatment is started (Adopted from NICE).

#### 3.2.3 Psychopharmacology adverse effects

▪ Full understanding and agreement *should* be documented in the medical records (GPP).

#### 3.2.4 Psychoeducation for the family

▪ Education and skills interventions *should* be offered to parents of all children and young people diagnosed with ASD (GPP).

#### 3.2.5 Sleep management

▪ Behavioural therapy *should* be considered for children and young people with ASD who experience sleep problems (Adopted from SIGN).
▪ Children with ASD who present with signs of possible obstructive sleep apnoea, or sleep‐disordered breathing (loud snoring, choking or periodic stopping of breathing during sleep) *should* be referred to sleep medicine services or ENT for assessment (Adopted from SIGN).
▪ In children with ASD who have sleep difficulties which have not been resolved following behavioural interventions, a trial of melatonin to improve sleep onset *should* be considered (Adopted from SIGN).

#### 3.2.6 Gastrointestinal and feeding interventions

▪ Gastrointestinal symptoms in children and young people with ASD *should* be managed in the same way as in children and young people without ASD (Adopted from SIGN).

#### 3.2.7 Transition of care from paediatrics to adulthood

▪ Local autism teams *should* ensure that young people with autism who are receiving treatment and care from paediatric services are reassessed at around 14 years to establish the need for continuing treatment into adulthood (Adopted from NICE).
▪ The timing of transition may vary locally and individually but *should* usually be completed by the time the young person is 18 years old. Variations should be agreed on by both child and adult services (Adopted from NICE).
▪ As part of the preparation for the transition to adult services, health and social care professionals *should* carry out a comprehensive assessment of the young person with autism (Adopted from NICE).

### 3.3 Implementation considerations

The full CPG document, including the complete recommendations and multifaceted categories of implementation tools, can be downloaded from the SHC website (Link: https://shc.gov.sa/Arabic/Evidences/Pages/CAccredited.aspx). Moreover, the CPG full document was provided as Supporting Information S1: File S1.

The ASD National CPG Implementation Tools included a baseline assessment template, clinical pathways, clinical scenarios with slide sets, do not do information, eight key performance indicators, the list of members and recommended qualifications of the ASD multidisciplinary team, ASD referral form, a list of factors associated with an increased prevalence of ASD, ASD screening tools, useful online links and resources, CBAHI ASD certification programme (for autism centres), National ASD Registry, and two online platforms to search and identify the ASD service providers in KSA (Links: https://shc.gov.sa/Arabic/NCDBD/servicecenterssite/Pages/default.aspx and https://acesaudi.org/center/), and patient and family educational information in Arabic (Supporting Information S1: File S1).

The NCDBD‐SHC launched a series of nationwide implementation face‐to‐face training workshops for the ASD Adapted CPG's recommendations in a number of cities, including Riyadh, Jeddah and Dhahran in addition to online webinars.

### 3.4 Discussion and further research recommendations

Between 2018 and 2020, a CPG for ASD was developed as part of the Prince Mohammed Bin Salman Programme for Autism and Developmental Disorders.^34^ This initial CPG provided recommendations for assessment, interventions and family‐centred practices. It also pointed out existing gaps in the ASD CPGs available at that time.

In the current National ASD CPG, a comprehensive suite of CPG implementation (CPGI) tools is included. These tools, six of which were adapted from the National Institute for Health and Care Excellence (NICE), range from baseline assessment to ‘do not do’ recommendations and various templates and clinical pathways.^27^

New CPGI tools were also created based on these adapted guidelines. These tools cover a broad spectrum, including the qualifications for an ASD Multidisciplinary Team and a compilation of ASD assessment and screening tools detailing their validation status and availability in Arabic. They also feature algorithms, patient and family information in Arabic, and two directories of service providers for ASD in KSA provided by the Autism Center of Excellence and the NCDBD‐SHC.

Additionally, four significant national supportive initiatives have been connected to these CPGI tools: the CBAHI National ASD Centres Standards, the National Survey on ASD, Autism Digital Certificates and the Rights of People with Disabilities.^33^

Published evidence identified the limited knowledge about ASD and recommended educational, media and social media campaigns that focus on improving the public attitude and knowledge about ASD terminology and treatment.^35^, ^36^ Another review highlighted the importance of culturally and linguistically sensitive methods for diagnosing and evaluating ASD, offering an innovative viewpoint for upcoming research and best practices in KSA.^37^

Alkhonezan et al. emphasized the significance of improved social support and acknowledgement of anxious parents and families of children with ASD in the KSA healthcare context.^38^ Another cross‐sectional study investigated the influence of child and family characteristics on the age of ASD diagnosis in KSA.^39^

#### 3.4.1 Facilitators and barriers during the ASD national guideline adaptation process and potential solutions for the challenges in Saudi Arabia

Facilitators play a crucial role in the adaptation process of national guidelines for ASD in Saudi Arabia. Among these facilitators were recruiting dedicated professionals, including clinicians, researchers and guideline methodologists, who possess a deep understanding of both the local context and international best practices. Their expertise allowed for the identification of high‐quality practice guidelines, evidence‐based interventions and strategies that are culturally relevant and responsive to the unique needs of people with ASD and their families in Saudi Arabia. Moreover, the collaboration between various stakeholders, such as the organizing governmental organization, subject matter experts, guideline methodology experts, and a patient advocate, provides a comprehensive approach to guideline adaptation, ensuring diverse and holistic perspectives are considered. The improving and rising accessible specialized and advanced ASD services, including diagnostic facilities, early intervention programmes, and trained professionals, poses a considerable facilitator to effective guideline implementation.

Potential enablers of the CPGI include national strategies and models of care, national committees, initiatives and new ASD healthcare services. The National ASD Centres of Excellence and related professional bodies also play a crucial role in disseminating and implementing this CPG.^31^ The increasing integration and coordination among national healthcare sectors in neurodevelopmental health and rehabilitation services as part of the healthcare transformation is another strength.

However, several barriers hindered the national guideline adaptation project. One significant challenge is the limited awareness and understanding of ASD among the general population and even some healthcare professionals. This lack of awareness can lead to misconceptions, stigma and delayed diagnosis and intervention. Furthermore, cultural factors, such as societal attitudes towards disability and traditional beliefs about causation and treatment, can influence the acceptance and uptake of evidence‐based practices for ASD.

To address these ongoing challenges, concerted efforts are needed to enhance awareness, education and training regarding ASD throughout KSA. This includes implementing public awareness campaigns, providing professional development opportunities for healthcare providers and educators, and integrating ASD‐related content into academic curricula. Increasing access to diagnostic and intervention services, particularly in underserved regions with limited resources, is also critical. Additionally, fostering community engagement and collaboration can help combat stigma and promote acceptance and inclusion of individuals with ASD. By addressing these challenges through a multifaceted approach, KSA can better implement this nationally adapted guideline for ASD, ultimately improving outcomes for individuals and families affected by this condition.

Challenges remain, such as the limited access to specialized ASD healthcare services in remote areas, primary care providers' lack of awareness of the latest ASD CPGs, lack of public awareness about ASD, and suboptimal transitions from paediatric to adult ASD healthcare services.^31^, ^32^, ^34^

The ‘KSU‐Modified‐ADAPTE’ method was instrumental in the development of this CPG, offering a clear, structured approach with supporting tools and templates for the CPGA process.^12^ Including a patient and family advocate in the Expert Reference Group (ERG) also strengthened the project, ensuring that the adapted CPG was informed by those directly affected by ASD.

Finally, the GAG has recommended seven key implementation strategies, including leadership engagement and support, clinical and quality champions support, dissemination strategies, regular training and education, regular audit and feedback, networking with existing projects, and patients and families as champions for change.

Additionally, seven topics were recommended for future research and policy improvements to enhance the quality and safety of healthcare for children and people with ASD in KSA (Supporting Information S1: File S1).

In conclusion, this adapted CPG presents practical, evidence‑based guidance with implementation tools for managing children with ASD in KSA. The project illustrated the applicability of the KSU‑Modified‑ADAPTE and highlighted the importance of collaboration between clinicians and methodologists for adapting national CPGs.

## AUTHOR CONTRIBUTIONS

**Shuliweeh Alenezi**: Conceptualization; project administration; supervision; resources; writing—original draft; funding acquisition; methodology; writing—review and editing; visualization; investigation; data curation; validation. **Fahad Bashiri**: Investigation; data curation; writing—review and editing. **Amel Alawami**: Investigation; writing—review and editing; data curation. **Ayman Alhazmi**: Investigation; writing—review and editing; data curation. **Somayyah Aladamawai**: Investigation; writing—review and editing; data curation. **Faisal Alnemary**: Investigation; writing—review and editing; data curation. **Yasser Alqahtani**: Investigation; writing—review and editing; data curation. **Maysaa Buraik**: Investigation; writing—review and editing; data curation. **Saleh AlSuwailem**: Investigation; writing—review and editing; data curation. **Shahad Akhalifah**: Investigation; writing—review and editing; data curation. **Saleh Al‐Salehi**: Writing—review and editing; investigation; data curation. **Yasser Amer**: Conceptualization; methodology; investigation; writing—original draft; writing—review and editing; data curation; visualization; supervision; validation; resources. All authors have reviewed and agreed to the submitted version of the manuscript.

## CONFLICT OF INTEREST STATEMENT

The authors declare no conflict of interest.

## ETHICS STATEMENT

Not applicable.

## PERMISSION TO REPRODUCE MATERIAL FROM OTHER SOURCES

We were granted official permissions from the developer organizations to use their four source clinical practice guidelines for our National ASD guideline adaptation project in Saudi Arabia, as mentioned on page 104 of the adapted full guideline document. The email messages can be obtained from the clinical chair (Shuliweeh Alenezi).

## Supporting information

SUPPLEMENTARY MATERIAL
**PDF File: Evidence‐based clinical practice guideline for the management of children with autism spectrum disorder (ASD).** First Edition (Ramadan 1444/April 2023) SHC‐CPG‐02.

Supporting information.

## Data Availability

The data that support the findings of this study have been made available in the tables, figures, and Supplementary material of this article. Further details could be made available from the contact information of the National Center for Developmental Behavioural Disorders (Email: nccdd@shc.gov.sa) and from the authors upon reasonable request to the corresponding authors.
