# Supplementary material for: Saudi Arabian evidence‐based clinical practice guideline for the management of children with autism spectrum disorder: A national guideline adaptation using the KSU‐modified‐ADAPTE methodology
Source: Clin Public Health Guidel. 2024 Nov 21;1(5):e70001. doi: 10.1002/gin2.70001 (PMC13563337; doi:10.1002/gin2.70001)
Supplement: Supplementary file 1 [file GIN2-1-e70001-s001.pdf]

SAUDI HEALTH COUNCIL
NATIONAL CENTER FOR
DEVELOPMENTAL BEHAVIORAL
DISORDERS (NCCDD)

المجلس الصحي السعودي
Saudi Health Council

EVIDENCE-BASED CLINICAL
PRACTICE GUIDELINE FOR

MANAGEMENT OF CHILDREN WITH AUTISM SPECTRUM DISORDER (ASD)

First Edition
Ramadan 1444 - April 2023
SHC-CPG-02

صائب
Approved
Center for Evidence-Based Medicine

المجلس الصحي السعودي
Saudi Health Council

المجلس الصحي السعودي
Saudi Health Council

المركز الوطني لاضطرابات النمو الشامل

الدليل الإرشادي للممارسة السريرية المبنية على البراهين لتدبير الأطفال ذوي اضطراب طيف التوحد

الاصدار الأول

Contents

Disclaimer 03

Acknowledgements 04

Abbreviations 05

Guideline Registration 06

Guideline Adaptation 08

External Review Group (ERG) 12
- Reviewer of the Clinical Content 13
- Reviewer of the Adaptation Methodology 13

Saudi Health Council (SHC) Approvals 14
- CPG Overview 15
- The Developers of the source CPGs 15
- Adaptation Process Methodology 15

Introduction 16

Scope and Purpose 20
- Disease/Condition 21
- Guideline Objective(s) 21
- Health or Clinical Questions (PIPOH Model) 21

Recommendations 22
- Definitions of Quality of Evidence and Strength of Recommendations 23
- Recommendations 24
  - Prevention and Early Identification 24
  - Early recognition and diagnosis (DSM-5 and ICD-10-AM) 24
  - Assessment & Psychometric Assessment Tools 25
  - Telehealth Setting (Regional and Remote Locations) 26
  - Risk factors associated with ASD 27
  - Referral for Diagnostic Clarification 27

Diagnosis 28
- Assessment Setting 29
  - Clinic Setting 29
  - Community Setting 29
- Diagnostic criteria 30
- Use of Autism Spectrum Disorder Multidisciplinary Team Assessment 30
- Components of Assessment 31
- Timing of diagnosis 38
- Differential diagnosis 38
- Medical investigations 39
- Disclosure of the results of diagnostic assessment 39
- Information and support for families 41

Contents

**Interventions**	**44**
Problem minimization and avoidance	45
Treatment goals	45
Physical wellbeing and needs	46
Non-pharmacological Interventions	46
Development of Care Plan	47
ASD Multidisciplinary Team (ASD-MDT)	48
Functional assessment of behavior	48
Psychosocial interventions for behavior	49
Developmental interventions	49
Interventions for social communication skills	49
Intensive behavioral interventions	50
Educational assistance/Support and planning for transition	50
Occupational Therapy	50
Parent training and coaching for occupational therapy	51
Sensory integration and Others	51
Parent mediated interventions	51
Cognitive Behavioral Therapy (CBT)	51
Pharmacological Interventions	52
Antipsychotics (conventional and atypical)	52
ADHD Medication	53
Stimulants	54
Non-stimulant	54
Antidepressants-Selective Serotonin Reuptake Inhibitors (SSRIs)	54
Antianxiety and benzodiazepines	54
Psychopharmacology adverse effects	55
Psycho-education for the family	55
Special cases or comorbidities	55
Sleep Management	55
Sleep medication:	56
Gastrointestinal and feeding interventions	57
Transition of care from pediatrics to adulthood	58
Living in the community	59
Recreation and leisure	59
**Recommendations for future research and health policy makers**	**60**
**Implementation**	**62**
Implementation Strategies	63
Implementation Tools	63
**Patient and Family information in Arabic**	**84**
**Plan for Scheduled Review and Update**	**88**
**List of Funding Sources**	**90**
**References**	**92**
**Appendices**	**96**
Appendix 1. List of the websites and databases we searched	97
Appendix 2. Search strategy (MEDLINE)	98
Appendix 3. PRISMA 2020 flow diagram	99
Appendix 4. Adaptation Process Methodology	100
Phase One – Set-Up	101
Phase Two – Adaptation	102
Phase Three – Finalization	105

Disclaimer

Clinical Practice Guidelines (CPGs) are intended to serve as an aid to clinical judgment but are in no way a substitute for a medical professional's independent judgment and should not be considered medical advice. This CPG is a working document that reflects the state of the field at the time of publication and is based upon the accessible best updated published evidence. Because rapid changes in this area are expected, periodic revisions are inevitable. Standards of care are determined on the basis of all clinical data available for an individual case and are subject to change as scientific knowledge and technology advance and patterns of care evolve, these parameters of practice should be considered CPGs only. The presented recommendations may not be appropriate in all situations.

Adherence to the CPG recommendations will not ensure a successful outcome in every case, nor should they be construed as including all proper methods of care or excluding other acceptable methods of care aimed at the same results. Any decision by practitioners to apply these CPGs must be made in light of local resources and individual patient circumstances. The ultimate judgment regarding a particular clinical procedure or treatment plan must be made by the appropriate healthcare professional(s) responsible for clinical decisions regarding a specific clinical situation; the doctor. This judgment should only be arrived at following discussion of the options with the patient, in light of the diagnostic and treatment choices available. However, it is advised that significant departures from a national CPG or any local CPG derived or adapted from it should be fully documented in the patient's medical records at the time the relevant decision is taken.

Intellectual Property Rights

All intellectual property rights are reserved to the Saudi Health Council, National Center for Developmental Behavioral Disorders (NCCDD), and the Authorship group. No part of this publication may be reproduced or transmitted in any form or by any means without permission in writing from the institution and the authors. Permission to use the recommendations of the source CPGs was sought from Their developer organizations .

Printed in Riyadh, Saudi Arabia (1444 - 2023).

Acknowledgements

The Saudi Health Council, National Center for Developmental Behavioral Disorders (NCCDD), would like to present their highest gratitude and acknowledgment to the members of the clinical practice guideline (CPG) adaptation group and the external review group for their distinguished efforts in finalizing this first evidence-based CPG for Autism Spectrum Disorder in Saudi Arabia.

The ASD Adapted CPG was critically appraised using the AGREE II Instrument by the National Center for Evidence-Based Medicine, Saudi Health Council.

Abbreviations

- **ACRC** – THE COOPERATIVE RESEARCH CENTRE FOR LIVING WITH AUTISM (AUTISM CRC), AUSTRALIA
- **ADHD** – ATTENTION DEFICIT HYPERACTIVITY DISORDER
- **ASD** – AUTISM SPECTRUM DISORDER
- **CBT** – COGNITIVE BEHAVIORAL THERAPY
- **CPG** – CLINICAL PRACTICE GUIDELINE
- **DSM-5** – DIAGNOSTIC AND STATISTICAL MANUAL OF MENTAL DISORDERS (5TH EDITION)
- **GAG** – GUIDELINE ADAPTATION GROUP
- **GPP** – GOOD PRACTICE POINT
- **GIN** – GUIDELINES INTERNATIONAL NETWORK, SCOTLAND, UK
- **ICD-10-AM** – INTERNATIONAL STATISTICAL CLASSIFICATION OF DISEASES AND RELATED HEALTH PROBLEMS, TENTH REVISION, AUSTRALIAN MODIFICATION
- **IPGRP** – INTERNATIONAL PRACTICE GUIDELINE REGISTRY PLATFORM
- **NICE** – NATIONAL INSTITUTE FOR HEALTH AND CARE EXCELLENCE, UK
- **NZMOH** – NEW ZEALAND MINISTRY OF HEALTH GUIDELINE GROUP, NEW ZEALAND
- **SIGN** – SCOTTISH INTERCOLLEGIATE GUIDELINES NETWORK, UK
- **SHC** – SAUDI HEALTH COUNCIL, SAUDI ARABIA
- **SSRI** – SELECTIVE SEROTONIN REUPTAKE INHIBITORS

Guideline Registration

Guideline Registration

- **PREPARE** (Practice guideline REgistration for trancPAREncy), World Health Organization (WHO) Collaborating Centre for Guideline Implementation and Knowledge Translation, Evidence-Based Medicine Center, University of Lanzhou, Lanzhou, China. Registration number: IPGRP-2022CN002.
  Link: http://www.guidelines-registry.org/guid/1385?lang=en

- **GIN** International Guidelines Library and Registry, Guidelines International Network (GIN), Perth, Scotland
  Link: https://guidelines.ebmportal.com/node/70209

Guideline Adaptation

Guideline Adaptation

Name of the Working Group:

Guideline Adaptation for Management of Children with ASD.

Project Leader and Clinical Chair:

Dr. Shuliweeh Alenezi

Methodology Chair:

Dr. Yasser S. Amer

Guideline Authorship:

The GAG has decided on group authorship and stated the order of authorship.

Table 1. Members of the Guideline Adaptation Group (GAG)

Full Name	Affiliation(s)	Role/ Clinical specialty
Dr. Shuliweeh Alenezi, MBBS, FRCP, FAPA, Med. Edu.	Psychiatry Department, College of Medicine, King Saud University, Riyadh, Saudi Arabia	Clinical Expert (Associate Professor, Consultant Child and Adolescent Psychiatrist)<br>Clinical Chair, GAG<br>Project Lead
Prof. Fahad A. Bashiri , MBBS, FRCP	Pediatric Neurology Division, Pediatrics Department, College of Medicine, King Saud University, Riyadh, Saudi Arabia	Clinical Expert (Professor, Consultant Pediatric Neurologist and Epileptologist)

Full Name	Affiliation(s)	Role/ Clinical specialty
Dr. Amel Hussain Alawami, MD, MPA	Johns Hopkins Aramco Healthcare, Saudi Arabia	Clinical Expert (Consultant Developmental Behavioral Pediatrician)<br>Member
Dr. Ayman Shawqi Alhazmi, MD	Developmental Pediatric Department, Children's Hospital, King Saud Medical City, Ministry of Health, Riyadh, Saudi Arabia	Clinical Expert (Consultant Developmental Behavioral Pediatrician)<br>Member<br>Member
Dr. Somayyah A. Al-adamawi, MD	1. King Abdullah bin Abdulaziz University Hospital, Riyadh, Saudi Arabia<br>2. College of Medicine, Princess Nourah bint Abdulrahman University, Riyadh, Saudi Arabia	Clinical Expert (Assistant Professor, Consultant Developmental Behavioral Pediatrician)<br>Member
Dr. Faisal Alnemary, PhD, BCBA	Autism Center of Excellence, Riyadh, Saudi Arabia	Clinical Expert (Board Certified Behavior Analyst)<br>Member
Dr. Yasser Alqahtani, MD	King Fahad Armed Forces Hospital, Jeddah, Makkah Region, Saudi Arabia	Clinical Expert (Consultant Child and Adolescent Psychiatrist)<br>Member
Dr. Maysaa W. Buraik, PsyD	John Hopkins Aramco Healthcare, Dhahran, Saudi Arabia	Clinical Expert (Consultant, Clinical Psychology)<br>Member
Mr. Saleh AlSuwailem, MSc	King Abdullah bin Abdulaziz University Hospital, Riyadh, Saudi Arabia	Clinical Expert (Senior Speech and Language Therapist)<br>Member

Full Name	Affiliation(s)	Role/ Clinical specialty
Ms. Shahad Mohammad Akhalifah, MSc, PGCert.	Autism Center of Excellence, Riyadh, Saudi Arabia	Clinical Expert (Senior Occupational Therapist) Member
Dr. Yasser Sami Amer, MBBCh, M. Pediatrics, M. HCI, CPHQ, FISQua, IPFPH	1. Pediatrics Department, King Khalid University Hospital, Riyadh, Saudi Arabia<br>2. Clinical Practice Guidelines, Quality Research, Quality Management, King Saud University Medical City, Riyadh, Saudi Arabia<br>3. Research Chair for Evidence-Based Health Care & Knowledge Translation, King Saud University, Riyadh, Saudi Arabia<br>4. Egyptian Pediatrics Clinical Practice Guidelines Committee, Egypt<br>5. Adaptation Working Group, Guidelines International Network, Scotland, UK	Guideline Methodologist General Pediatrician Methodology Chair, GAG

Logistic and support group

- Ms. Latifa Abdullah AlDrees
- Ms. Maram Abdulrahman Binsabbar
- National Center for Developmental Behavioral Disorders (NCDBD) Saudi Health Council

Contact Information

Email: nccdd@shc.gov.sa
Phone: +966 11 457 9300/2206

The Design

Mr. Yaman Alsebai

External Review Group (ERG)

- Reviewer of the Clinical Content 13
- Reviewer of the Adaptation Methodology 13

External Review Group

Reviewer of the Clinical Content:

Dr. Monirah Albloushi
Assistant Professor, College of Nursing, King Saud University, Riyadh, Saudi Arabia

Dr. Muhammad Al-Heizan
Assistant Professor, Occupational Therapy Consultant, Department of Rehabilitation Sciences, Occupational Therapy Program College of Applied Medical Sciences, King Saud University, Riyadh, Saudi Arabia

Mr. Majed Alshakhori
Clinical Psychiatric Pharmacist
Pharmacy Services Department, Johns Hopkins Aramco health care, Dhahran

Nouf Alzrayer, PhD., BCBA-D
King Saud University,
Riyadh, Saudi Arabia

Ms. Ghada AlSanea
Medical Technologist, King Faisal Specialist Hospital & Research Center ASD Patient Representative, Riyadh, Saudi Arabia

Dr. Eiman H. Alismail
Consultant Clinical Neuropsychologist
King Faisal Specialist Hospital & Research Center for Autism Research,
Riyadh, Saudi Arabia

Dr. Majid I. Alshahwan
Consultant Speech & Language Pathologist
Speech & Hearing Program, Department of Rehabilitation Sciences, College of Applied Medical Sciences, King Saud University,
Riyadh, Saudi Arabia

Dr. Yasser Ad-Dab'bagh
Consultant, Psychoanalyst and Child and Adolescent Psychiatrist,
Department of Mental Health, Neuroscience Center King Fahad Specialist Hospital – Dammam (KFSH-D) Dammam, Saudi Arabia

Dr. Saleh Mohammed AlSalehi, MD
Assistant Professor and Consultant,
Developmental and Behavioral Pediatrics,
Child Development Center, King Abdullah bin Abdulaziz University Hospital,
Princess Norah bint AbdulRahman University,
Riyadh, Saudi Arabia

Reviewer of the Adaptation Methodology

Prof. Lubna A. Al-Ansary
Professor and Consultant of Family Medicine, Family and Community Medicine Department,
College of Medicine, King Saud University,
Founder, Research Chair for Evidence-Based Health Care and Knowledge Translation, King Saud University
Chairperson, Clinical Practice Guidelines Steering Committee, King Saud University Medical City,
Riyadh, Saudi Arabia
Former Assistant Director General, Metrics and Measurement, World Health Organization, Geneva, Switzerland

Saudi Health Council (SHC) Approvals

CPG Overview	15
The Developers of the source CPGs	15
Adaptation Process Methodology	15

Saudi Health Council (SHC) Approvals

Dr. Ahmed Aboabat	Director, National Center of Developmental and Behavioral Disorders.
Dr. Nahar Alazemi	Secretary General, Saudi Health Council, Riyadh, Saudi Arabia.

CPG Overview

CPG Title	Evidence Based Clinical Practice Guideline for Management of Children with ASD.
CPG Code	SHC-CPG-02
CPG Release Date	1444 - 2023
Status	Adapted using 'KSU Modified ADAPTE' CPG Adaptation Methodology.[8]
Printed Copies	Available upon request from the SHC.
Electronic Sources	Available on the official website of the SHC.

The Developers of the source CPGs

- Australian Autism CRC (ACRC 2018), Australia
- National Institute for Health and Care Excellence (NICE 2017), UK
- Ministry of Health New Zealand (NZ 2016), New Zealand
- Scottish Intercollegiate Guidelines Network (SIGN), Healthcare Improvement Scotland (HIS) (SIGN 2019), UK

Adaptation Process Methodology

The description of the methodology for the production of this CPG can be fulfilled by utilizing the sequential process for trans-contextual adaptation of CPGs 'KSU Modified ADAPTE' as one of the formal adaptation methodologies for CPGs. Details process is reported in the Appendices.

Introduction

Introduction

Autism Spectrum Disorder (ASD) is a neurodevelopmental disorder with onset in early childhood. A diagnosis of ASD requires the presence of impairment in two major domains: Communication and social interaction, and restricted or repetitive patterns of behavior [1]. ASD is considered multifactorial in origin where multiple genetic and epigenetic factors may contribute to the etiology [2]. Research on ASD is relatively limited in developing countries, including Saudi Arabia, and thus most of the current clinical practices in Saudi Arabia are based on conclusions from studies conducted in Western developed countries [3]. Although a marked growth in local research started to emerge in 2008, a lot has yet to be investigated. For instance, the prevalence of ASD in Saudi Arabia has not yet been accurately determined. One study stated that 42,500 cases were diagnosed with autism in [4] 2002. A more recent systematic review showed that the prevalence of ASD in Arabian Gulf countries, including Saudi Arabia was, ranging from 1.4 to 29 per 10,000 population [5]. This is, in fact, lower than the prevalence rate measured by studies conducted in developed countries (77-39 per 10,000). This does not necessarily mean that it is less prevalent in Saudi Arabia; as the discrepancy could be explained by methodological flaws, the limited capacity to diagnose ASD, and lower level of awareness of ASD among parents which reduces the likelihood of recognizing symptoms and consequent attempts to access care [6]. A cross-sectional study involving 205 individuals with ASD, showed that the ratio between males to female in that sample was 4.9:1. Psychiatric comorbidities were found in %65 of the patients. Among these comorbidities, attention deficit hyperactivity disorder (ADHD) was the most prevalent (%53), followed by intellectual disability (%8), then epilepsy and cerebral palsy (%2 for each) [7].

In Saudi Arabia, there is a large gap between the needs of individuals diagnosed with ASD and the currently available services. Although Saudi Arabia is a large country, most of the services are localized in its largest cities (i.e., Riyadh, Jeddah, or Dammam) [7]. Furthermore, the provided services tend not to meet the satisfaction of families. In Saudi, a study that used a scale of excellent-good-acceptable-or-bad to evaluate publicly provided services for ASD showed that about %75 of the families scored them as acceptable or bad [7]. Attesting to difficulties in accessing services in Saudi Arabia, Khan et al., in a recent cross-sectional study published in 2020, found that only %31 of children on the autism spectrum could access nearby autism centers, and %72 have no access to private schools for ASD in their area. [8]. The areas that are targeted through early intervention are language, play skills and social participation, activities of daily living (ADLs), and challenging behaviors [9]. Moreover, pharmacological management can be initiated to target specific symptoms

Introduction (Continue)

related to ASD, such as irritability, hyperactivity, aggression, aberrant social behavior, repetitive behavior, inattention, and insomnia. However, all medications might cause side effects that need to be monitored by a professional, so it should be prescribed by either a neurologist or a psychiatrist [10].

The lack of governmental and institutional support for a child on the autism spectrum places a significant burden on parents, which might affect the quality of life for other siblings. It has been shown that almost %88.5 of parents of children on the autism spectrum in Saudi Arabia experience significant distress. The challenges and unmet needs of children on the autism spectrum resulting from lack of access to needed resources resulted in negative impacts on social and financial aspects of their lives, their family relationships, and the quality of life of the siblings of the children on the autism spectrum[11]. In another cross-sectional study that involved 61 families of individuals diagnosed with ASD, %37 of the families felt embarrassed for having children on the autism spectrum, while almost %64 were bothered by the way that others in the community dealt with them [6].

Currently, several authorities are involved in the journey of care, education, and empowerment for individuals diagnosed with ASD and others with other developmental disabilities. The initial screening (i.e., especially at a young age) and diagnostic services are carried out by specialized and trained healthcare professionals (i.e., general pediatricians, psychiatrists, child psychologists, developmental-behavioral or neurodevelopmental pediatricians, pediatric neurologists, or child psychiatrists) working in entities affiliated with the Ministry of Health (MoH) or accredited by public authorizing entities, such as Saudi Health Council (SHC), Saudi Commission for Health Specialties (SCFHS), and Saudi Central Board of Accreditation of Healthcare Institutions (CBAHI). This discrepancy in carrying out such services with different capacities might be attributed to the lack of clear guidance, shortage of specialized professionals, and availability of validated tools.

For post-diagnostic services, rehabilitation services are overseen by three entities: the MoH, the Ministry of Education (MoE), and the Ministry of Human Resources and Social Development (MHRSD), where services are provided through governmental (free-of-charge) providers or private (pay-for-services or covered by health insurance) ones (e.g., hospitals, schools, special needs centers, specialized clinics). While MHRSD is overseeing and subsidizing intervention services that private centers provide to children who

Introduction (Continue)

are not in school because they are younger than six years old or are severely impacted (IQ > 50), MoE is serving students on the autism spectrum that do not have severe intellectual disability and have effective verbal communication in their public school systems, including some institutions that are designated for students with disabilities, special-needs classrooms, or mainstreamed (inclusion) classrooms along with their typical peers. Intervention and special education services may vary from one center to another. Generally, the number of intervention hours received and its structure (group-format vs. individualized-format) varies mainly based on the limited service slots, ranging from 20 hours/week to only a few. However, about %80 of families of individuals on the autism spectrum accessing early intervention services pay for them out-of-pocket and almost %92 of families reported an excessive financial burden related to the management of the needs of their children on the autism spectrum [6]. As such, the status of services in Saudi Arabia might require a thorough review of the needs of individuals on the autism spectrum and their families and concerted efforts to attempt to meet them.

The aim of this adapted clinical practice guideline (CPG) is to provide evidence-based recommendations for screening, surveillance, diagnosis, assessment and clinical and therapeutic interventions for children with ASD. It aims to improve the experience of children and those who care for them. These recommendations were adapted from the relevant CPGs developed by the Australian Autism CRC (AACRC 2018) [11], Ministry of Health New Zealand (NZ 2016) [12], National Institute of Clinical and Health Excellence (NICE 2017) [13], and Scottish Intercollegiate Guidelines Network (SIGN) Healthcare Improvement Scotland (HIS) [14] using a formal methodology for CPG adaptation: the KSU-Modified-ADAPTE [17-15].

Scope and Purpose

- Disease/Condition 21
- Guideline Objective(s) 21
- Health or Clinical Questions (PIPOH Model) 21

Scope and Purpose

Disease/Condition:

Autism spectrum disorders (ASD) is a continuum of neurodevelopmental disorders characterized by early-onset impairments in social communication and interaction, as well as by restricted, repetitive interests and behaviors (DSM-5).

Guideline Objective(s):

The aim of this adapted clinical practice guideline (CPG) is to provide evidence-based recommendations for management including screening, surveillance, diagnosis, assessment and clinical and therapeutic interventions for children with ASD. It aims to improve the experience of children and those who care for them

Health or Clinical Questions (PIPOH Model):

P: Patient (Target Population):

Children with autism spectrum disorders (ASD). Both genders. Age group from 2 to 18 years.

Common comorbidities: ADHD, anxiety, and other relevant medical problems.

I: Interventions and Practices Considered / CPG Category:

(Refer to Recommendations Section).

P: Professionals (Intended / Target Users or Stakeholders):

Physicians, pharmacists, nurses, therapists, technicians.

Clinical Specialties:

Physicians: psychiatrists, pediatricians (behavioral developmental, neurology), medical genetics, physical medicine and rehabilitation, mental health specialists.

Clinical Pharmacists, nurses, speech therapy, Special education, Occupational therapy, and (patient/ family representative).

O: Major Outcomes Considered:

1. Death (Mortality).
2. Major neurodevelopmental disability in surviving children (Morbidity).

H: Healthcare Settings:

Primary, secondary, and tertiary neonatal healthcare services mainly nurseries, NICUs, and outpatient clinics.

Recommendations

Definitions of Quality of Evidence and Strength of Recommendations 23
Key Recommendations 24

Definitions of Quality of Evidence and Strength of Recommendations

The source CPGs from NICE and SIGN have followed the GRADE method (Grading of Recommendations, Assessment, Development, and Evaluation) their CPG development methodology 13,14. Recommendatins obtained from other gudilnes converted based on thier level of evidence and grading to ensure connsistnecy.
Strength of Recommendations (adopted from the NICE Manual)
https://www.nice.org.uk/process/pmg20/chapter/writing-the-guideline#wording-the-recommendations.

Must: Recommendations That Must (or Must Not) Be Followed:
The words 'must' or 'must not' are generally only used if there is a legal duty to apply the recommendation. Occasionally the word 'must' (or 'must not') has been used if the consequences of not following the recommendation could be extremely serious or potentially life threatening.

Should: Recommendations That Should (or Should Not) Be Followed – a 'Strong' Recommendation:
The word 'offer' (and similar words such as 'refer' or 'advise') has been used when there is a good degree of confidence that, for the vast majority of patients, an intervention will do more good than harm, and be cost effective. Similar forms of words (for example, 'Do not offer…') are used when an intervention will not be of benefit for most patients.

Could: Recommendations That Could Be Followed:
The word 'consider' is used when an intervention will do more good than harm for most patients, and will be cost effective, but other options may be similarly cost effective. The choice of intervention, and whether or not to have the intervention at all, is more likely to depend on the patient's/caregiver's values and preferences than on the strength of a recommendation, and so the healthcare professional should spend more time considering and discussing the options with the patient.

Recommendations

PREVENTION AND EARLY IDENTIFICATION:

Early recognition and diagnosis (DSM-5 and ICD-10-AM):

01 | Early identification should be achieved by (NZ MOH):

- Comprehensive developmental surveillance of all children so deviations from normal development are recognized early.
- Valuing and addressing parental concerns about their child's development.
- Referral to diagnostic services when risk for ASD is confirmed by using early screening tools.
- Ensuring data collection and audit of the pathway takes place.

02 | Clinical assessment should incorporate a high level of vigilance for features suggestive of ASD, in the domains of social interaction and play, speech, language and communication difficulties and behaviour (SIGN).

03 | The assessment of children and young people with developmental delay, emotional and behavioral problems, psychiatric disorders, impaired mental health or genetic syndromes should include surveillance for ASD as part of routine practice (SIGN).

04 | Instruments may be used for information gathering, but they should not be used to make or rule out a referral for an assessment for ASD (SIGN).

05 | Healthcare professionals should consider informing families that there is a substantial increased risk of ASD in siblings of affected children (SIGN).

06 | It is worth noting that good eye contact, and smiling and showing affection to family members should not rule out the autism diagnosis (GPP).

Recommendations

Assessment & Psychometric Assessment Tools:

Be aware that tools to identify children and young people with an increased likelihood of autism may be useful in gathering information about signs and symptoms of autism in a structured way but are not essential and should not be used to make or rule out a diagnosis of autism (NICE). Also be aware that:

- a positive score on tools to identify an increased likelihood of autism may support
- a decision to refer but can also be for reasons other than autism
- a negative score does not rule out autism.

When referring children and young people to the autism team, include in the referral letter the following information (NICE):

- reported information from parents, carers and professionals about signs and/or symptoms of concern
- your own observations of the signs and/or symptoms.

When referring children and young people to the autism team, include in the referral letter the following information, if available (NICE):

- antenatal and perinatal history
- developmental milestones
- factors associated with an increased prevalence of autism (see Implementation tools)
- relevant medical history and investigations
- information from previous assessments.

Explain to parents or carers and, if appropriate, the child or young person, what will happen on referral to the autism team or another service (NICE).

Recommendations

Telehealth Setting (Regional and Remote Locations)

Rural families experience significant challenges and disadvantages in accessing resources and services (eg, transportation, lack of local support, isolation, financial resources, lack of employment opportunities) should be considered compared with urban dwellers (GPP).

Distance education programmes could be one way to make information accessible. One based on written materials with weekly telephone contact and one delivered through videoconferencing platform (GPP).

Contact by telephone, the Internet, informal support groups and joining ASD and/or disability associations should be encouraged (GPP).

Telehealth Considerable feedback was received about the use of telehealth during the assessment process and should be encouraged. While there was a broad agreement that technology can facilitate better access to assessment services for individuals living in rural and remote (GPP).

It is recommended that in circumstances where a clinician with the professional background and assessment expertise prerequisites to being a member of the autism diagnosis team is not present in the local community, a partnership between local clinicians and an assessment team in another location be facilitated through other methods (ACRC).

In regional and remote settings, there may not be any medical or allied health professionals with clinical expertise in ASD. In some remote settings, a relevant clinician could visit the community only a limited number of times each year (GPP).

Recommendations

Risk factors associated with ASD

Standardized ASD assessment interviews and schedules should be used (NZ MOH).

The intellectual, adaptive and cognitive skills associated with ASD should be seriously considered and, where possible and appropriate, formally assessed (NZ MOH).

Referral for Diagnostic Clarification

It is suggested that a referral for an assessment of ASD concerns should be initiated by a primary healthcare provider. This individual's professional discipline may differ between private and public healthcare settings (ACRC).

It is recommended that the healthcare provider must receive formal professional training in typical child development and the signs and/or symptoms of common neurodevelopmental and behavioural conditions, including those associated with ASD, as well as common co-occurring and differential diagnosis conditions (ACRC).

If the primary healthcare professional administers clinical assessments as part of the process for initiating a referral for an assessment of ASD concerns, they should have training and expertise in administering these assessments (with all prerequisites for using the instrument in clinical practice met) (ACRC).

Health care professionals must have a good understanding of the different forms of expression of ASD symptomatology across developmental stages and the symptomatology of common coexisting and alternative conditions (NZ MOH).

DIAGNOSIS

Assessment Setting	29
▪ Clinic Setting:	29
▪ Community Setting:	29
Diagnostic criteria	30
Use of Autism Spectrum Disorder Multidisciplinary Team Assessment	30
Components of Assessment	31
Timing of diagnosis	38
Differential diagnosis	38
Medical investigations	40
Disclosure of the results of diagnostic assessment	40
Information and support for families	41

Assessment Setting:

Clinic Setting

It is recommended that a clinic setting should be considered an appropriate, but not essential, venue for an assessment of ASD concerns (ACRC).

Community Setting

- It is recommended that information about an individual's presentation in all community settings relevant to their daily life must be collected (ACRC).
- It is recommended that information about an individual's presentation in community settings must be obtained by one or more members of the Assessment Team through a combination of (ACRC):
  - direct observation in the community setting - asking the client(s) about behaviour in the community setting during an interview or through a questionnaire or survey.
  - observation of video recordings of the individual in the community setting that have been recorded and supplied by the client or other professional(s) with the client's permission.
  - verbal or written communication about the client's behaviour in the community setting from other professional(s).
- It is recommended that functional assessments must take place in a setting where the client feels comfortable and confident to discuss their level of functioning and support needs. This may be in a clinic, community or telehealth setting. Information is to be collected about the individual's level of functioning in all relevant community settings, though it is not essential for the clinician to make direct observations at these locations (ACRC).

Diagnostic criteria:

All professionals involved in diagnosing ASD in children, young people or adults should consider using the current version of either ICD-10-AM or DSM-5. The classification system used for diagnosis should be recorded in the patient's notes (SIGN).

Use of Autism Spectrum Disorder Multidisciplinary Team Assessment:

The Autism Spectrum Disorder Multidisciplinary Team (ASD-MDT) should have the skills and competencies to (NICE):

- carry out an autism diagnostic assessment.
- communicate with children and young people with suspected or known autism, and with their parents and carers, and sensitively share the diagnosis with them.

ADS-MDT members should (NICE):

- provide advice to professionals about whether to refer children and young people for autism diagnostic assessments.
- decide on the assessment needs of those referred or when referral to another service will be needed.
- carry out the autism diagnostic assessment.
- share the outcome of the autism diagnostic assessment with parents and carers, and with children and young people if appropriate.
- with parent or carer consent and, if appropriate, the consent of the child or young person, share information from the autism diagnostic assessment directly with relevant services, for example through a school visit by an autism team member.
- offer information to children, young people and parents and carers about appropriate services and support.

It is recommended that all clinicians involved in the assessment of ASD concerns obtain relevant training and expertise covering all the following areas (ACRC):

- developmental assessment (typical/atypical).
- Neurodevelopmental disorders clinical presentation.
- signs and symptoms associated with common co-occurring or differential diagnosis conditions.
- the criteria for ASD and co-occurring or differential diagnosis conditions.

Components of Assessment:

Refer first to a pediatrician or pediatric neurologist (if this has not already happened) children or young people (NICE):

- older than 3 years with regression in language.
- of any age with regression in motor skills.
- The pediatrician or pediatric neurologist can refer back to the ASD-MDT if necessary.

The Autism Case Coordinator should (NICE):

- act as a single point of contact for the parents or carers and, if appropriate, the child or young person being assessed, through whom they can communicate with the rest of the autism team.
- keep parents or carers and, if appropriate, the child or young person, up-to-date about the likely time and sequence of assessments.
- arrange the provision of information and support for parents, carers, children and young people as directed by the autism team.
- gather information relevant to the autism diagnostic assessment.

Discuss with the parents or carers and, if appropriate, the child or young person, how information should be shared throughout the autism diagnostic assessment, including communicating the outcome of the assessment. Take into account, for example, the child or young person's age and ability to understand (NICE).

Include in every autism diagnostic assessment (NICE):

- detailed questions about parent's or carer's concerns and, if appropriate, the child's or young person's concerns.
- details of the child's or young person's experiences of home life, education and social care.
- a developmental history, focusing on developmental and behavioural features consistent with ICD-10 or DSM-5 criteria (consider using an autism-specific tool to gather this information).
- assessment (through interaction with and observation of the child or young person) of social and communication skills and behaviours, focusing on features.
- a medical history, including prenatal, perinatal and family history, and past and current health conditions.

- a physical examination.
- consideration of the differential diagnosis.
- Systematic assessment for conditions that may coexist with autism.
- Occupational therapy should be considered where relevant.
- Assessment of language, cognitive, adaptive, motor, and sensory domains.

Perform a general physical examination and look specifically for (NICE):

- skin stigmata of neurofibromatosis or tuberous sclerosis using a Wood's light.
- signs of injury, for example self-harm or child maltreatment (see the National Family Safety Program).
- congenital anomalies and dysmorphic features including macrocephaly or microcephaly.

Consider which assessments are needed to construct a profile for each child or young person, for example (NICE):

- intellectual ability and learning style.
- academic skills.
- speech, language and communication.
- fine and gross motor skills.
- adaptive behavior (including self-help skills).
- mental and emotional health (including self-esteem).
- physical health and nutrition.
- sensory sensitivities.
- behavior likely to affect day-to-day functioning and social participation.
- socialization skills.

Use information from all sources, together with clinical judgment, to diagnose autism based on ICD-10-AM or DSM-5 criteria (NICE).

Do not rely on any autism-specific diagnostic tool alone to diagnose autism (NICE).

Be aware that in some children and young people there may be uncertainty about the diagnosis of autism, particularly in (NICE):

- children younger than 24 months.
- children or young people with a developmental age of less than 18 months.

- children or young people for whom there is a lack of available information about their early life (for example, some looked-after or adopted children).
- older teenagers' children or young people with a complex coexisting mental health disorder (for example ADHD, conduct disorder, a possible attachment disorder), sensory impairment (for example severe hearing or visual impairment), or a motor disorder such as cerebral palsy.

Be aware that some children and young people will have features of behavior that are seen in the autism spectrum but do not reach the ICD-10-AM or DSM-5 diagnostic criteria for definitive diagnosis. Based on their profile, consider referring to appropriate services (NICE).

Carry out an autism diagnostic assessment if there is regression in language or social skills in a child younger than 3 years (NICE).

Carry out an autism diagnostic assessment if there is regression in language or social skills in a child younger than 3 years (NICE).

If there is uncertainty after the autism diagnostic assessment about the diagnosis, consider keeping the child or young person under review, taking into account any new information (NICE).

If any of the following apply after assessment, consider obtaining a second opinion (including referral to a specialized tertiary ASD-MDT if necessary) (NICE):

- continued uncertainty about the diagnosis.
- disagreement about the diagnosis within the ASD-MDT.
- disagreement with parents or carers or, if appropriate, the child or young person, about the diagnosis.
- a lack of local access to particular skills and competencies needed to reach a diagnosis in a child or young person who has a complex coexisting condition, such as a severe sensory or motor impairment or mental health problem.
- a lack of response as expected to any therapeutic interventions provided to the child or young person.

During the autism diagnostic assessment, consider any potential risk of harm to, and from, the child or young person and take appropriate action (NICE).

Address the impact of other important considerations, such as intellectual and/or communication capacity, culturally, linguistically and/or socio-economically diverse background, regional or remote location, or complex psychosocial factors, on the assessment of ASD concerns (ACRC).

In cases of absence of ASD-MDT Diagnostic Team, it is recommended that a Single Clinician Diagnostic Evaluation be conducted by a clinician who, in addition to the expertise required by all members of the Assessment Team, has relevant training and expertise in the following areas (ACRC):

- clinical reasoning in weighing evidence, and performing diagnostic formulations and decisions.
- signs and symptoms associated with common co-occurring or differential diagnosis conditions.
- the criteria for ASD and co-occurring or differential diagnosis conditions described by the current version of international diagnostic manuals (e.g. DSM-5 and/or ICD-10-AM).

Single Clinician Diagnostic Evaluation must include (ACRC):

- Medical practitioner who holds specialist registration with the SCFHS in the field of community child health, general paediatrics, Child and adolescent psychiatry & pediatric neurology.
- Medical practitioner (family medicine, pediatrician) who holds general or specialist registration with the SCFHS and has at least six years of relevant experience, training or supervision in the assessment of neurodevelopmental disorders.
- Plus:
    - ☐ Occupational therapist who holds registration with the pediatric Occupational Therapy Board.
    - ☐ Clinical / Neuro- psychology who holds general registration, with training or supervision in the assessment of neurodevelopmental disorder.
    - ☐ Pediatric speech pathologist who is eligible to be a Certified Practising Member of Speech Pathology.

It is recommended that members of the ASD-MDT should obtain and maintain the additional skills and expertise listed above through peer observation, peer supervision and peer mentoring. Formal training courses and/or further qualifications may supplement these peer learning approaches (ACRC).

It is recommended that the Single Clinician should obtain and maintain the additional skills and expertise listed in Recommendation 38 through peer observation, peer supervision, and peer mentoring. Formal training courses and/or further qualifications may supplement these peer learning approaches (ACRC).

It is suggested that a Single Clinician Diagnostic Evaluation should involve the collection of information from at least one other clinician from a different discipline or specialty to the Single Clinician, if information from at least one clinician from a different discipline has not yet been obtained (for example, from the Comprehensive Needs Assessment (ACRC).

it is recommended that information should be collected during a Single Clinician Diagnostic Evaluation on the following (ACRC):

- overview of topics covered in the Comprehensive Needs Assessment.
- signs and/or symptoms specified in diagnostic criteria for ASD and potential co-occurring and/or differential conditions.
- biological, personal and environmental factors relevant to the individual.
- communication with clinicians who conducted the Comprehensive Needs Assessment.
- file review of any additional assessment reports.
- interview with the client.
- observation of the individual undergoing assessment.
- communication with other professional(s) as required.

It is suggested that the clinician who conducted the Single Clinician Diagnostic Evaluation invite additional clinician(s) as required to participate in the ASD-MDT Diagnostic Evaluation, based on the match between professional expertise and the area(s) of diagnostic uncertainty identified during the Single Clinician Diagnostic Evaluation. This should involve at least one other professional from a different discipline or specialty to the clinician who conducted the Single Clinician Diagnostic Evaluation (ACRC).

It is recommended that ASD-MDT Diagnostic Evaluation should include at least one additional clinician who meets at least one of the following eligibility criteria (ACRC):

- Medical practitioner who holds registration with the SCFHS in the field of
- community child health, general paediatrics, psychiatry or neurology

  Plus:

  - ASD-MDT members:
    - Medical practitioner.
    - Nurse.
    - Occupational therapist.
    - Psychologist: At least Senior clinical psychology/ neuropsychology.
    - Social worker.
    - Speech pathologist.

It is recommended that clinicians conducting the ASD-MDT Diagnostic Evaluation should use their clinical judgement to reach a consensus diagnostic decision by (ACRC):

- taking into account all information collected during all stages of assessments, in the context of a biopsychosocial framework
- integrating and weighing the available evidence against each diagnostic criterion (according to the current version of the DSM or ICD)
- testing alternative explanations for signs and/or symptoms that may warrant co-occurring or differential diagnosis or alternative clinical pathways
- considering if sufficient information is available to make a diagnostic decision with high confidence

It is recommended that information should be collected during a ASD-MDT Diagnostic Evaluation through a variety of means, including (ACRC):

- review of documentation from the Comprehensive Needs Assessment and Single Clinician Diagnostic Evaluation.
- communication with clinicians who conducted the Comprehensive Needs Assessment and Single Clinician Diagnostic Evaluation.
- file review of any additional assessment reports.
- interview with the client as required.
- observation of the individual undergoing assessment.
- administration of standardised and non-standardised assessments as required.
- communication with other professional(s) as required.

Other Professionals (ACRC):

The Assessment Team should liaise with other medical, allied health, disability and/or educational professionals to obtain further information about the individual being assessed, to support the Comprehensive Needs Assessment and Diagnostic Evaluation.

These other professionals are not part of the Assessment Team; however, their input may be helpful to obtain a more complete clinical picture of the individual's presentation in their everyday environment or provide specialist guidance to explore alternative explanations for presenting signs and/or symptoms.

This includes:

- Accredited practising dietitian.
- Audiologist.
- Board-certified behaviour analyst.
- Childcare worker.
- Dentist.
- Disability employment support person.
- Early intervention service provider.
- Gastroenterologist.
- General practitioner.
- Geneticist.
- Community health worker.
- Neurologist.
- Nurse.
- Occupational therapist.
- Ophthalmologist.
- Optometrist.
- Pediatrician.
- Physiotherapist.
- Preschool or early childhood teacher.
- Primary or secondary school teacher.
- Physiotherapist.
- Psychiatrist.
- Psychologist.
- Sleep and respiratory physician.
- Social worker.
- Special education teacher.
- Speech pathologist.

Timing of diagnosis:

- Regardless of the findings of any earlier assessments, referral for further assessment for ASD should be considered at any age (SIGN).
- Start the autism diagnostic assessment within 3 months of the referral to the autism team (NICE).
- ASD should be part of the differential diagnosis for preschool children displaying absence of age appropriate developmental features, as typical ASD behaviours may not be obvious in this age group (SIGN).

Differential diagnosis:

- It is recommended that, at each stage of the Diagnostic Evaluation, the clinicians must collect and evaluate information to consider the full range of clinical explanations for the presentation of signs and/or symptoms, and test these possible explanations against the evidence for an ASD diagnosis in the context of other differential and co-occurring diagnoses (ACRC).
- It is recommended that members of the ASD-MDT be highly familiar with the range of differential diagnoses for ASD (ACRC).
- Clinicians without the clinical qualifications or expertise to adequately evaluate potential differential diagnoses for a given individual should not undertake the assessment of ASD concerns (ACRC).
- Healthcare professionals should recognise that children and young people with ASD may also have additional developmental disorders, medical problems or emotional difficulties/disorders and should have access to the same range of therapeutic interventions as any other child (SIGN).
- Consider the following other diagnoses for autism and whether specific assessments are needed to help interpret the autism history and observations (NICE):

- **Medical or genetic problems and disorders:**
  - epilepsy and epileptic encephalopathy.
  - chromosome disorders.
    - genetic abnormalities, including:
      - fragile X.
      - tuberous sclerosis.
      - muscular dystrophy.
      - neurofibromatosis.
  - **Functional problems and disorders:**
    - feeding problems, including restricted diets.
    - urinary incontinence or enuresis.
    - constipation, altered bowel habit, faecal incontinence or encopresis.
    - sleep disturbances.
    - vision or hearing impairment.

Consider the following differential diagnoses for autism and whether specific assessments are needed to help interpret the autism history and observations (NICE):

- **Neurodevelopmental disorders:**
  - specific language delay or disorder.
  - a learning (intellectual) disability or global developmental delay.
  - developmental coordination disorder (DCD).
- **Mental and behavioural disorders:**
  - attention deficit hyperactivity disorder (ADHD).
  - mood disorder - anxiety disorder.
  - attachment disorders.
  - oppositional defiant disorder (ODD).
  - conduct disorder.
  - obsessive compulsive disorder (OCD).
  - psychosis.
- **Conditions in which there is developmental regression:**
  - Rett syndrome.
  - epileptic encephalopathy.
- **Other conditions:**
  - severe hearing impairment.
  - severe visual impairment.
  - maltreatment.
  - selective mutism.

Medical investigations:

Do not routinely perform any medical investigations as part of an autism diagnostic assessment (NICE).

Except where clinically relevant, the need for the following should be reviewed for all individuals with ASD (SIGN):

- examination of physical status, with particular attention to neurological and dysmorphic features.
- chromosomal microarray.
- examination of audiological status.
- investigations to rule out recognized etiologies of ASD (e.g. tuberous sclerosis).
- electroencephalography if there is suspicion of epilepsy.

Advice on further testing should be sought from the local genetics service (SIGN).

Disclosure of the results of diagnostic assessment:

After the autism diagnostic assessment discuss the findings, including the profile, sensitively, in person and without delay with the parents or carers and, if appropriate, the child or young person. Explain the basis of conclusions even if the diagnosis of autism was not reached (NICE).

For children and young people with a diagnosis of autism, discuss and share information with parents or carers and, if appropriate, the child or young person, to explain (NICE):

- what autism is.
- how autism is likely to affect the child or young person's development and function.

Provide parents or carers and, if appropriate, the child or young person, with a written report of the autism diagnostic assessment. This should explain the findings of the assessment and the reasons for the conclusions drawn. (NICE)..

Share information, including the written report of the diagnostic assessment, with the primary healthcare practitioner (NICE).

With parental or carer consent and, if appropriate, the assent of the child or young person, share information with key professionals involved in the child's or young person's care, including those in education and social care (NICE).

With parental or carer consent and, if appropriate, the assent of the child or young person, make the profile available to professionals in education (for example, through a school visit by a member of the autism team) and, if appropriate, social care. This is so it can contribute to the child or young person's individual education plan and needs-based management plan (NICE).

For children and young people with a diagnosis of autism, offer a follow-up appointment with an appropriate member of the autism team within 6 weeks of the end of the autism assessment for further discussion (for example about the conclusions of the assessment and the implications for the child or young person) (NICE).

For children and young people with a diagnosis of autism, discuss with parents or carers the risk of autism occurring in siblings and future children (NICE).

Information and support for families:

Provide individual information on support available locally for parents, carers, and autistic children and young people, according to the family's needs. This may include (NICE):

- **contact details for:**
  - local and national support organisations (who may provide, for example, an opportunity to meet other families with experience of autism, or information about specific courses for parents and carers and/or young people)
  - organisations that can provide advice on social and financial benefits from Ministry of Human Resources and Social Development and other relevant organizations
  - organisations that can provide information on educational support and social care
  - information to help prepare for the future, for example transition to adult services.

Make arrangements to support autistic children and young people and their family and carers during times of increased need, including major life changes such as puberty, starting or changing schools, or the birth of a sibling (NICE).

Explore with autistic children and young people, and their families and carers, whether they want to be involved in shared decision-making and continue to explore these issues at regular intervals (NICE).

If children and young people express interest, offer a collaborative approach to treatment and care that takes their preferences into account (NICE).

Ensure that all autistic children and young people have full access to health and social care services, including mental health services, regardless of their intellectual ability or any coexisting diagnosis (NICE).

Offer all families (including siblings) and carers verbal and written information about their right to (NICE):

- short breaks and other respite care.
- a formal carer's assessment of their own physical and mental health needs, and how to access these.

Offer families (including siblings) and carers an assessment of their own needs, including whether they have (NICE):

- personal, social and emotional support
- practical support in their caring role, including short breaks and emergency plans.
- a plan for future care for the child or young person, including transition to adult services.

When the needs of families and carers have been identified, discuss help available locally and, taking into account their preferences, offer information, advice, training and support, especially if they (NICE):

- need help with the personal, social or emotional care of the child or young person, including age-related needs such as self-care, relationships or sexuality.
- are involved in the delivery of an intervention for the child or young person in collaboration with health and social care professionals.

Interventions

- Problem minimization and avoidance 45
- Treatment goals 45
- Physical wellbeing and needs 46
- Non-pharmacological Interventions 46
  - Development of Care Plan 47
  - ASD-MDT 48
  - Functional assessment of behavior 48
  - Psychosocial interventions for behavior 49
  - Developmental interventions 49
  - Interventions for social communication skills 49
  - Intensive behavioral interventions 50
  - Educational assistance/Support and planning for transition 50
  - Occupational Therapy 50
  - Parent training and coaching for occupational therapy 51
  - Sensory integration and Others 51
  - Parent mediated interventions 51
  - Cognitive Behavioral Therapy (CBT) 51
  - Pharmacological Interventions 52
    - Antipsychotics (conventional and atypical) 52
    - ADHD Medication 53
      - Stimulants 54
      - Non-stimulant 54
      - Antidepressants-Selective Serotonin Reuptake 54
      - Inhibitors (SSRIs) 54
      - Antianxiety and benzodiazepines 54
  - Psychopharmacology adverse effects 55
  - Psycho-education for the family 55
  - Special cases or comorbidities 55
  - Sleep Management 55
    - Sleep medication 56
  - Gastrointestinal and feeding interventions 57
  - Transition of care from pediatrics to adulthood 58

Problem minimization and avoidance:

Tests to avoid:

- avoid routine urine tests for metals and minerals in children with autistic behaviors as there is insufficient evidence on association between toxicological exposures and development of autistic behaviors – testing for metals and minerals may be harmful if treatment decisions are based on these results (GPP).
- avoid performing hair analyses for "environmental toxins" in children with behavioral or developmental disorders including autism (GPP).

It is recommended to avoid the use of the following interventions (NICE):

- Neurofeedback to manage speech and language problems in children and young people with autism
- Auditory integration training to manage speech and language problems in children and young people with autism.
- omega-3 fatty acids to manage sleep problems in Children and young people with autism.
- The following interventions to manage autism in any context in children and young people:
    - secretin.
    - chelation.
    - hyperbaric oxygen therapy.
    - exclusion diets (such as gluten- or casein-free diets) unless medically indicated.

Treatment goals:

Treatment should (NZ MOH):

- Encourage functional development.
- Teach skills for independent living.
- Minimize stress for the person with ASD, and their family and carers.

Physical wellbeing and needs:

Pre-treatment assessments should gather detailed information on behavioural, emotional and mental health difficulties, address differential diagnosis, screen for medical conditions and address environmental issues (NZ MOH).

Non-pharmacological Interventions:

Treatment plans should be comprehensive, and include behavioural needs, educational interventions, psychosocial treatments, communication, environmental and systems issues and the suitability (or not) of medication (NZ MOH).

Consider a specific social-communication intervention for the core features of autism in children and young people that includes play-based strategies with parents, carers and teachers to increase joint attention, engagement and reciprocal communication in the child or young person. Strategies should (NICE):

- be adjusted to the child or young person's developmental level.
- aim to increase the parents', carers', teachers' or peers' understanding of, and sensitivity and responsiveness to, the child or young person's patterns of communication and interaction.
- include techniques of therapist modelling and video-interaction feedback.
- include techniques to expand the child or young person's communication, interactive play and social routines.

The intervention should be delivered by a trained professional. For pre-school children consider parent, carer or teacher mediation. For school-aged children consider peer mediation (NICE).

Assess factors that may increase the risk of behavior that challenges in routine assessment and care planning in children and young people with autism including (NICE):

- impairments in communication that may result in difficulty understanding situations or in expressing needs and wishes.
- coexisting physical disorders, such as pain or gastrointestinal disorders.
- coexisting mental health problems such as anxiety or depression and other neurodevelopmental conditions such as ADHD.

- the physical environment, such as lighting and noise levels.
- the social environment, including home, school and leisure activities.
- changes to routines or personal circumstances.
- developmental change, including puberty.
- exploitation or abuse by others.
- inadvertent reinforcement of behaviour that challenges.
- the absence of predictability and structure.
- unmet emotional or attachment needs.

Development of Care Plan:

Develop a care plan with the child or young person and their families or carers that outlines the steps needed to address the factors that may provoke behavior that challenges, including (NICE):

- treatment, for example, for coexisting physical, mental health and behavioural problems.
- support, for example, for families or carers.
- necessary adjustments, for example, by increasing structure and minimising unpredictability.

If a child or young person's behavior becomes challenging, reassess factors identified in the care plan and assess for any new factors that could provoke the behaviour (NICE).

Offer the following to address factors that may trigger or maintain behavior that challenges (NICE):

- treatment for physical disorders, or coexisting mental health and behavioral problems.
- interventions aimed at changing the environment, such as:
  - providing advice to families and carers.
  - making adjustments or adaptations to the physical surroundings.

ASD Multidisciplinary Team (ASD-MDT)

If a child or young person's behavior becomes challenging, reassess factors identified in the care plan and assess for any new factors that could provoke the behaviour (NICE).

At the multidisciplinary review, take into account the following when choosing an intervention for behavior that challenges (NICE):

- the nature, severity and impact of the behaviour.
- the child or young person's physical and communication needs and capabilities.
- the environment.
- the support and training that families, carers or staff may need to implement the intervention effectively.
- the preferences of the child or young person and the family or carers.
- the child or young person's experience of, and response to, previous interventions.

If no coexisting mental health or behavioral problem, physical disorder or environmental problem has been identified as triggering or maintaining the behavior that challenges, offer the child or young person a psychosocial intervention (informed by a functional assessment of behavior) as a first-line treatment (NICE).

Functional assessment of behavior:

The functional assessment should identify (NICE):

- factors that appear to trigger the behavior.
- patterns of behavior.
- the needs that the child or young person is attempting to meet by performing the behavior.
- the consequences of the behavior (that is, the reinforcement received as a result of the behavior).

Psychosocial interventions for behavior:

Psychosocial interventions for behavior that challenges should include (NICE):

- clearly identified target behavior
- a focus on outcomes that are linked to quality of life
- assessment and modification of environmental factors that may contribute to initiating or maintaining the behavior
- a clearly defined intervention strategy that takes into account the developmental level and coexisting problems of the child or young person
- a specified timescale to meet intervention goals (to promote modification of intervention strategies that do not lead to change within a specified time)
- a systematic measure of the target behavior taken before and after the intervention to ascertain whether the agreed outcomes are being met
- consistent application in all areas of the child or young person's environment (for example, at home and at school)
- agreement among parents, carers and professionals in all settings about how to implement the intervention.

Developmental interventions:

Access to support from staff trained in applied behaviour analysis-based technologies (e.g. Picture Exchange Communication System, discrete trial training, task analysis, prompting, fading or shaping) to build independence in adaptive, communication and social skills should be considered for children with ASD (NICE).

Interventions for social communication skills:

Interventions to support communicative understanding and expression in individuals with ASD, such as the Picture Exchange Communication System and the use of environmental visual supports (e.g. in the form of pictures or objects), should be considered (SIGN).

Interventions to support social communication should be considered for children and young people with ASD, with the most appropriate intervention being assessed on an individual basis (SIGN).

Choice of interventions to support communication in children and young people with ASD should be informed by effective assessment (SIGN).

Adapting the communicative, social and physical environments of children and young people with ASD may be of benefit (options include providing visual prompts, reducing requirements for complex social interactions, using routine, timetabling and prompting and minimising sensory irritations) (SIGN).

Intensive behavioral interventions:

Early intensive behavioral intervention (EIBI) should be considered as a treatment of value for young children with ASD to improve outcomes such as cognitive ability, language skills, and adaptive behavior (NZ MOH).

Behavioral interventions could be considered to address a wide range of specific behaviors, including those that challenge, in children and young people with ASD, both to reduce symptom frequency and severity and to increase the development of adaptive skills (SIGN-GPP).

•Healthcare professionals should be aware that some challenging behaviors may be due to an underlying lack of skills development in the child/young person and also may represent an individual's strategy for coping with their difficulties and circumstances severity and to increase the development of adaptive skills (SIGN-GPP).

Healthcare professionals should be aware that factors in the social and physical environment may contribute to positive behaviors or those that challenge (SIGN-GPP)

Educational assistance/Support and planning for transition:

With parental or carer consent and, if appropriate, the assent of the child or young person, share information with key professionals involved in the child's or young person's care, including those in education and social care.

Education and skills interventions should be offered to parents of all children and young people diagnosed with ASD (NICE).

Occupational Therapy:

Children and young people affected by ASD may benefit from occupational therapy, advice and support in adapting environments, activities and routines in daily life (SIGN).

Parent training and coaching for occupational therapy:

Interventions within the scope of occupational therapy practice to improve performance in Activities of daily living (ADLs), Instrumental activities of daily living (IADLs), education, work, rest, and sleep for persons with ASD occupational therapy practice to improve social interaction, restricted and repetitive behaviors, play performance, and leisure participation for persons with ASD (GPP).

Sensory integration and Others:

Sensory behaviours should be taken into account when profiling the needs of individuals with ASD. (SIGN)

Delivering assessment and interventions in a physical environment that is appropriate for people with hyper- and/or hypo-sensory sensitivities. (NICE)

Parent mediated interventions:

Parent-mediated intervention programs should be considered for preschool children and young people who are affected by ASD, as they may help families interact with their child, promote development and increase parental satisfaction, empowerment and mental health. (SIGN)

Cognitive Behavioral Therapy (CBT):

Consider the following for children and young people with autism and mental health comorbidities as indicated who have the verbal and cognitive ability to engage in a cognitive behavioral therapy (CBT) intervention (NICE):

- Group CBT adjusted to the needs of children and young people with autism.
- Individual CBT for children and young people who find group-based activities difficult.

Consider adapting the method of delivery of CBT for children and young people with autism and anxiety to include (NICE):

- Emotion recognition training
- Greater use of written and visual information and structured worksheets
- A more cognitively concrete and structured approach
- Simplified cognitive activities, for example, multiple-choice worksheets
- Involving a parent or carer to support the implementation of the intervention, for example, involving them in therapy sessions
- Maintaining attention by offering regular breaks
- Incorporating the child or young person's special interests into therapy if possible.

Pharmacological Interventions:

Do not use the following interventions for the management of core features of autism in children and young people (NICE):

- antipsychotics.
- antidepressants.
- anticonvulsants.
- exclusion diets (such as gluten- or casein-free diets).

Antipsychotics (conventional and atypical):

Consider second generation antipsychotic medication for managing behavior (irritability and hyperactivity) that challenges children and young people with autism when psychosocial or other interventions are insufficient or could not be delivered because of the severity of the behavior (NICE).

Antipsychotic medication should be initially prescribed and monitored by a pediatrician or psychiatrist who should (NICE):

- identify the target behavior
- decide on an appropriate measure to monitor effectiveness, including frequency and severity of the behaviour and a measure of global impact
- review the effectiveness and any side effects of the medication after 3–4 weeks
- stop treatment if there is no indication of a clinically important response at 6 weeks.

- If antipsychotic medication is prescribed:
  - start with a low dose.
  - use the minimum effective dose needed.
  - regularly review the benefits of the antipsychotic medication and any adverse events including specific testing for metabolic side effects.

When choosing antipsychotic medication, should take into account side effects, acquisition costs, the child or young person's preference (or that of their parent or carer where appropriate) and response to previous treatment with an antipsychotic (NICE).

Patients and their carers should be advised of potential side effects before treatment is started.
Monitoring while using antipsychotic medication includes(NICE):

- Body mass Index and waist circumference measurement ideally monthly for the first 3 months of the initiation of medication then at 6 months then annually.
- HgbA1c, Fasting Blood Glucose, Fasting Lipid Profile at baseline, then at 3 months then annually: https://cdn.mdedge.com/files/s3fs-public/Document/September-0_051/2017913CP_SavvyPsych_FINAL.pdf

When prescribing is transferred to primary or community care, the specialist should give clear guidance to the practitioner who will be responsible for continued prescribing about (NICE):

- the selection of target behaviours
- monitoring of beneficial and side effects
- the potential for minimally effective dosing
- the proposed duration of treatment
- plans for stopping treatment.

ADHD Medication:

ADHD medication to treat comorbid ADHD and not the core symptoms of autism.

Associated ADHD coould be managed with stimulant medications such as Methylphenidate or Atomoxetine to reduce some symptoms (GPP).

Stimulants:

- Methylphenidate may be considered for management of attention difficulties/hyperactivity in children or young people with ASD (SIGN).
- Use of a test dose to assess if methylphenidate is tolerated could be considered in children prior to any longer trial (SIGN).
- Side effects should be carefully monitored (SIGN).

Non-stimulant:

- Atomoxetine can be used in children and young adults with autism as it may be associated with improved symptoms of hyperactivity/impulsivity and inattention in children and adolescents with autism spectrum disorder and ADHD symptoms (GPP).

Antidepressants-Selective Serotonin Reuptake Inhibitors (SSRIs):

- Selective serotonin reuptake inhibitors (SSRI) should not be used to manage core features of ASD (e.g., repetitive behaviors) in children and young people (SIGN).
- SSRIs should be considered for children and young people with comorbid symptoms (anxiety, depression and/or obsessive symptoms) on a case-by-case basis.
  Use SSRI with caution and careful monitoring (NZ MOH)
- There is insufficient evidence to make any recommendation about the use of other types of antidepressants (NZ MOH).

Antianxiety and benzodiazepines:

- Benzodiazepines can cause troublesome sedation and cognitive impairment in both short-term and long-term treatment, and tolerance and dependence can occur with prolonged use: and it is hard to identify those patients at risk of developing long-term problems (GPP).
- it is advisable to limit prolonged use of benzodiazepines and related gamma-aminobutyric-acid (GABA) agonists (GPP).

Psychopharmacology adverse effects:

- Healthcare clinicians need to discuss the potential side effects of any medication to the patient and/or the caregivers (GPP).
- Full understanding and agreement should be documented in the medical records (GPP).

Psycho-education for the family:

- Education and skills interventions should be offered to parents of all children and young people diagnosed with ASD (GPP).

Special cases or comorbidities:

- When severe behaviors are evident, people with ASD need to be assessed for co-morbid conditions such as seizures, attention deficit hyperactivity disorder (ADHD), anxiety disorders, depression, and gastrointestinal problems (NZ MOH).
- In severe or life-threatening situations, medication may be the best therapy (NZ MOH).

Sleep Management:

- If the child or young person with autism snores loudly, chokes or appears to stop breathing while sleeping, refer to a specialist to check for obstructive sleep apnoea and perform assessment that identifies (NICE):
    - what the sleep problem is (for example, delay in falling asleep, frequent waking, unusual behaviors, breathing problems or sleepiness during the day)
    - day and night sleep patterns, and any change to those patterns
    - whether bedtime is regular
    - what the sleep environment is like, for example:
        - the level of background noise.
        - use of a blackout blind .
        - a television or computer in the bedroom.
        - whether the child shares the room with someone.
    - presence of comorbidities especially those that feature hyperactivity or other behavioral problems.
    - levels of activity and exercise during the day

- possible physical illness or discomfort (for example, reflux, ear or toothache, constipation or eczema).
- effects of any medication.
- any other individual factors thought to enhance or disturb sleep, such as emotional relationships or problems at school.
- the impact of sleep and behavioral problems on parents or carers and other family members.

Behavioral therapy should be considered for children and young people with ASD who experience sleep problems (SIGN).

Develop a sleep plan (this will often be a specific sleep behavioral intervention) with the parents or carers to help address the identified sleep problems and to establish a regular night-time sleep pattern (NICE).

Ask the parents or carers to record the child or young person's sleep and wakefulness throughout the day and night over a 2-week period (NICE).

Use this information to modify the sleep plan if necessary and review the plan regularly until a regular sleep pattern is established (NICE).

Children with ASD who present with signs of possible obstructive sleep apnea, or sleep-disordered breathing (loud snoring, choking or periodic stopping of breathing during sleep) should be referred to sleep medicine services or ENT for assessment (SIGN).

Sleep medication:

Healthcare providers may use pharmacological intervention to aid sleep in the following situations (NICE):

- sleep problems persist despite following the sleep plan
- Sleep problems are having a negative impact on the child or young person and their family or carers.

In children with ASD who have sleep difficulties which have not resolved following behavioral interventions, a trial of melatonin to improve sleep onset should be considered (SIGN).

Use of melatonin should follow consultation with a pediatrician or psychiatrist with expertise in the management of sleep medicine in children and/or ASD, and be in conjunction with behavioral interventions (SIGN).

- Melatonin prescription should be reviewed regularly in the context of any emerging possible side effects and/or reduced therapeutic effect (SIGN)..
- Regularly review to evaluate the ongoing need for a pharmacological intervention and to ensure that the benefits continue to outweigh the side effects and risks (NICE).
- Obtain an adequate baseline sleep diary before any trial of melatonin (SIGN).
- Continue sleep hygiene measures (bedtime and wake-up routine, avoidance of day-time sleep) and a sleep diary, during any medication trial (SIGN).
- If the sleep problems continue to impact on the child or young person or their parents or carers, consider (NICE):
    - referral to a pediatric sleep specialist
    - advice for short breaks with help of family members or house helper
- Short breaks may need to be repeated regularly to ensure that parents or carers are adequately sleeping (NICE).

Gastrointestinal and feeding interventions

- Be aware that feeding problems, including restricted diets can result in nutritional deficiencies that may have serious consequences (NICE).
- Assess for any feeding, growth or nutritional problems, including restricted diets. Monitor and refer if needed (NICE).
- As part of a full nutritional assessment and monitoring, blood tests to check for nutritional deficiencies may be required (NICE).
- Gastrointestinal symptoms in children and young people with ASD should be managed in the same way as in children and young people without ASD (SIGN).
- Advice on diet and food intake should be sought from a dietician for children and young people with ASD who display significant food selectivity and dysfunctional feeding behavior, or who are on restricted diets that may be adversely impacting on growth, or producing physical symptoms of recognised nutritional deficiencies or intolerances (SIGN).

Transition of care from pediatrics to adulthood

- Local autism teams should ensure that young people with autism who are receiving treatment and care from pediatric services are reassessed at around 14 years to establish the need for continuing treatment into adulthood (NICE).
- If continuing treatment is necessary, make arrangements for a smooth transition to adult services and give information to the young person about the treatment and services they may need (NICE).
- The timing of transition may vary locally and individually but should usually be completed by the time the young person is 18 years. Variations should be agreed by both child and adult services (NICE).
- As part of the preparation for the transition to adult services, health and social care professionals should carry out a comprehensive assessment of the young person with autism (NICE).
- The assessment should make best use of existing documentation about personal, educational, occupational, social and communication functioning, and should include assessment of any coexisting conditions, especially depression, anxiety, ADHD, obsessive-compulsive disorder (OCD) and global delay or intellectual disability (NICE).
- Involve the young person in the planning and, where appropriate, their parents or carers (NICE).
- Provide information about adult services to the young person, and their parents or carers (NICE).
- During transition to adult services, consider a formal meeting involving health and social care and other relevant professionals from child and adult services (NICE).

Living in the community

- Careful and timely attention should be paid to planning for people with ASD leaving school and moving into further and post-compulsory education, work (paid or unpaid) or vocational services (NICE).
- Providers of further and post-compulsory education should ensure that their members of staff are aware of the specific educational needs of people with ASD (NICE).
- Work (paid and unpaid) should be considered an option for all people with ASD, regardless of their intellectual ability (NICE).
- Any known support needs of people with ASD, including those relating to cognitive, communication abilities and their sensory needs/sensitivities should be taken into account when transitioning into any work environment. Supported employment services for people with ASD should be developed (NICE).
- Vocational services of a high standard should be available to people with ASD who are not ready or able to access post-compulsory education and work (NICE).

Recreation and leisure

- All children and adults with ASD should have access to leisure facilities and meaningful activity tailored to their needs and interests. This is supported by person-centred plans designed by staff who have received specialist education for the role using strategies to promote social inclusion. Plans should be regularly evaluated (NZ MOH).
- Leisure and recreation planning should be included in a student/young person's transition program and this information shared with post school providers (NZ MOH).

Recommendations for future research and health policy makers

Recommendations for future research and health policy makers

Gathering information in schools or nurseries: Does routine additional information from educational settings (such as nursery or school) improve accuracy in diagnosing autism among children or young people up to the age of 19 compared with signs and symptoms alone?

Training professionals: Does training professionals to recognise signs and symptoms of autism lead to earlier assessment of needs and earlier diagnosis (and by implication reduce morbidity/improve health outcomes) among children and young people with suspected autism compared with no training.

Additional assessments: Do additional assessments (for IQ, language ability and motor ability) improve accuracy in diagnosing autism among preschool children (younger than 5 years) compared with signs and symptoms alone?

Comparative genomic hybridisation array: What is the effectiveness and acceptability of comparative genomic hybridisation (CGH) array compared with current genetic testing in children and young people with identified autism?

Telehealth and occupational therapy services protocols: what are the best practice protocols to guide telehealth and occupational services?

ASD assessment tools: Conduct validation studies for ASD assessment tools (e.g. ADOS).

Adaptation for the interventions (e.g. ESDM, triple P, Sensory etc.).

Implementation

Implementation Strategies 63

Implementation Tools 63

Implementation Strategies:

Several implementation frameworks and manuals have recommended the following strategies or interventions[15]:

1. Leadership commitment, engagement, and support.
2. Local clinical and quality champions.
3. Dissemination (printed and electronic).
4. Regular training and education.
5. Regular audit and feedback (along with regular review and update promotes the concept of the 'living CPGs'.
6. Networking with relevant existing projects
7. Parents or carers as champions for changes

Implementation Tools:

Tools from NICE CPG13: Link: https://www.nice.org.uk/guidance/cg128/resources

NICE Baseline assessment tool

this tool could be adapted according to the recommendations selected for pilot or full implementation of the CPG.

NICE Clinical scenarios with slides sets (8 cases)13

Clinical Algorithms (7 flowcharts)

Note: An explanation of each item is included in the Recommendations section of this CPG.

NICE Do not do information

Do not rely on any autism-specific diagnostic tool alone to diagnose autism. Do not routinely perform any medical investigations as part of an autism diagnostic assessment, but consider the following in individual circumstances and based on physical examination, clinical judgment and the child or young person's profile: -genetic tests, as recommended by your regional genetics centre, if there are specific dysmorphic features, congenital anomalies and/or evidence of intellectual disability -electroencephalography if there is suspicion of epilepsy.

Autism spectrum disorder overview

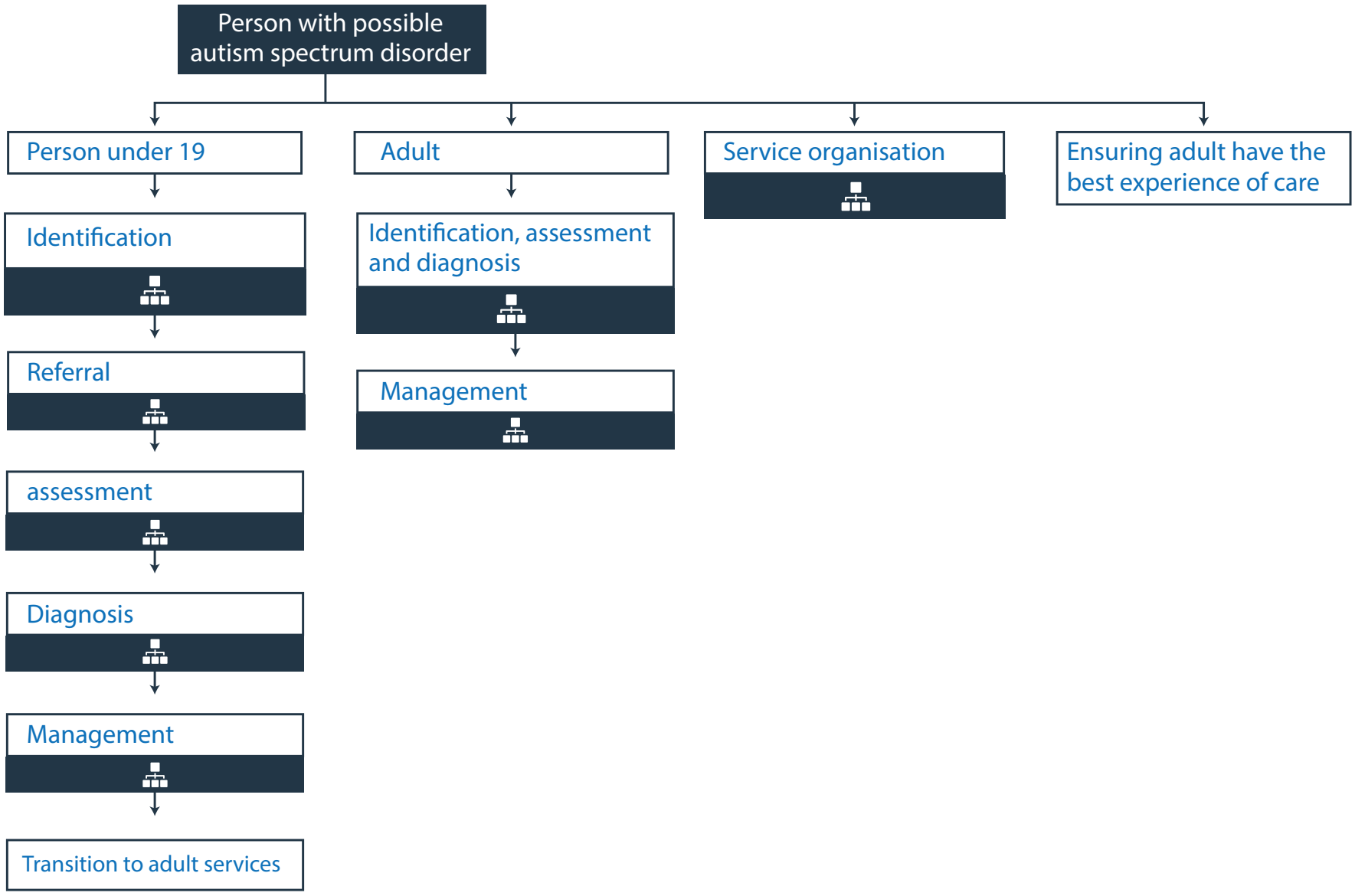

Referral of under 19s with pssible autism spectrum disorder

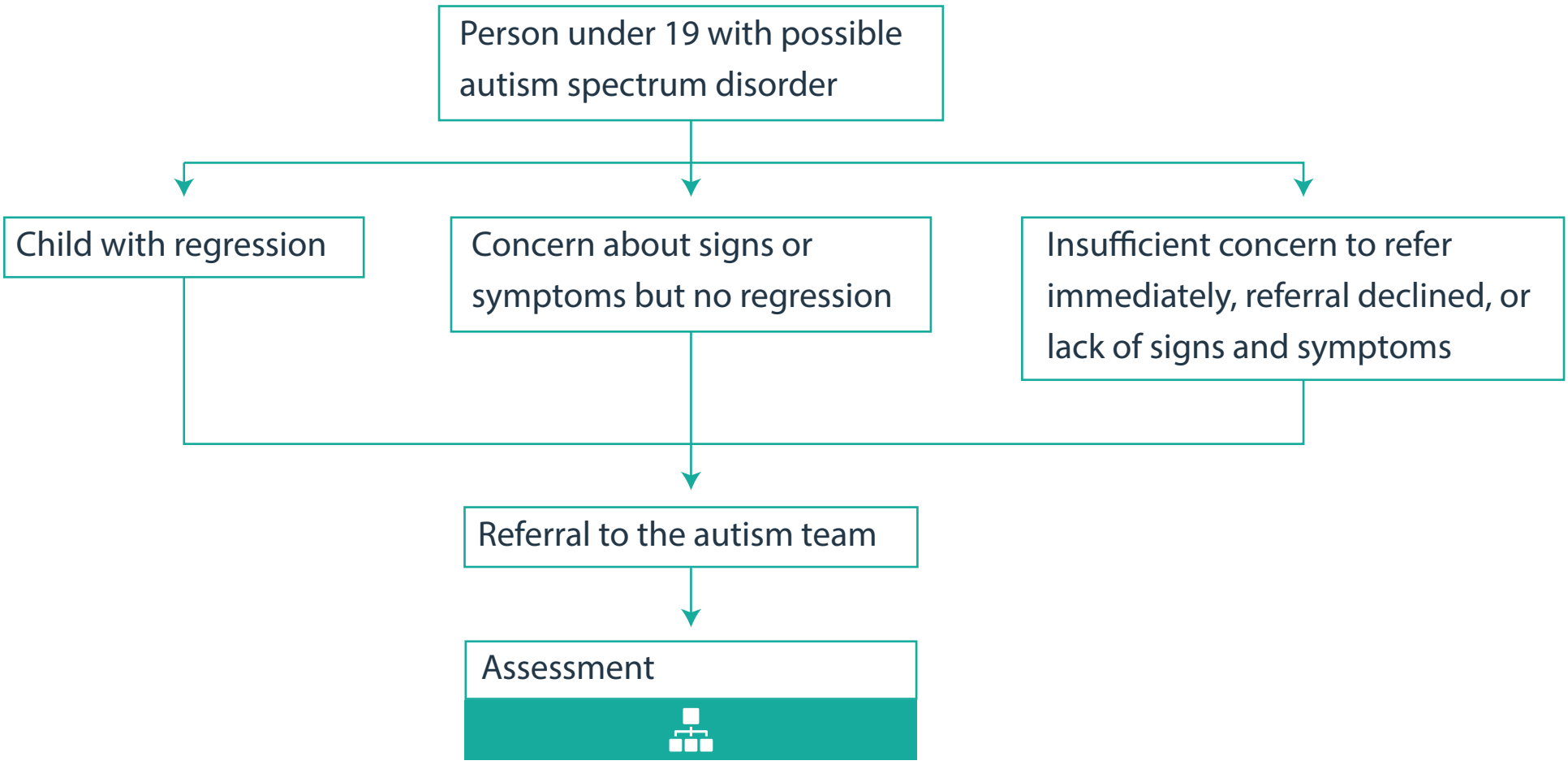

Early Identification and Diagnosis of Children Suspected of ASD

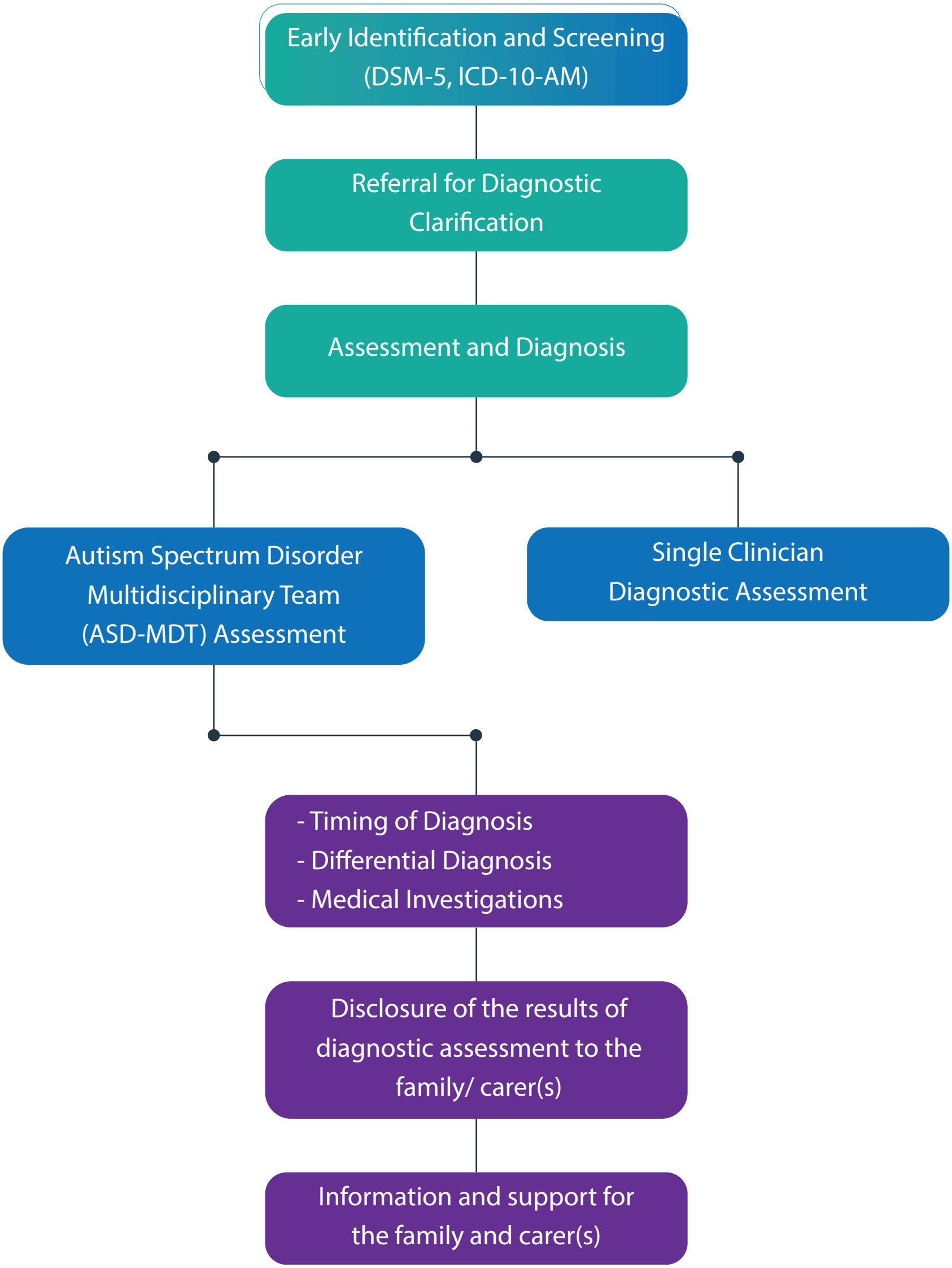

ASD managment overview

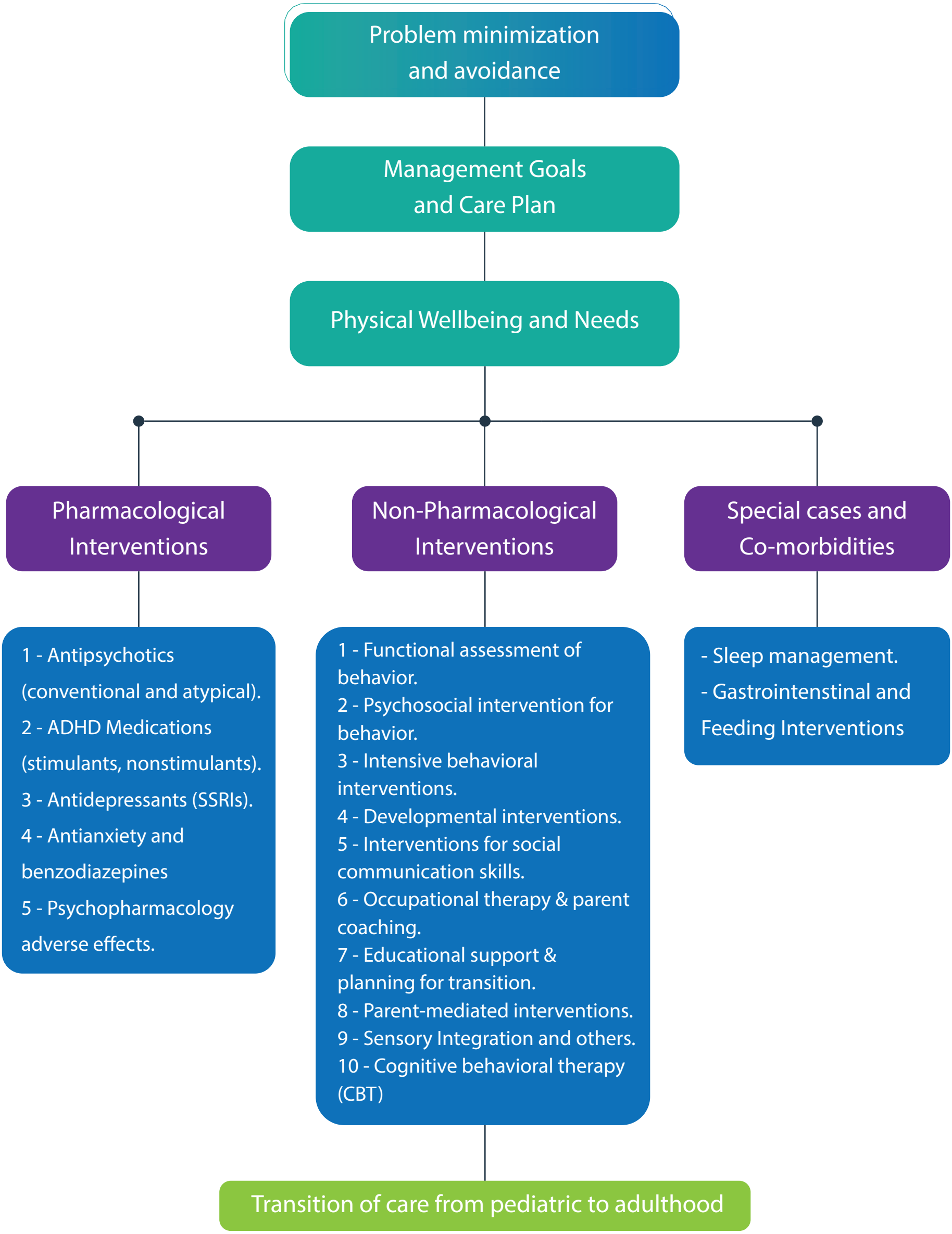

Sleep management algorithm

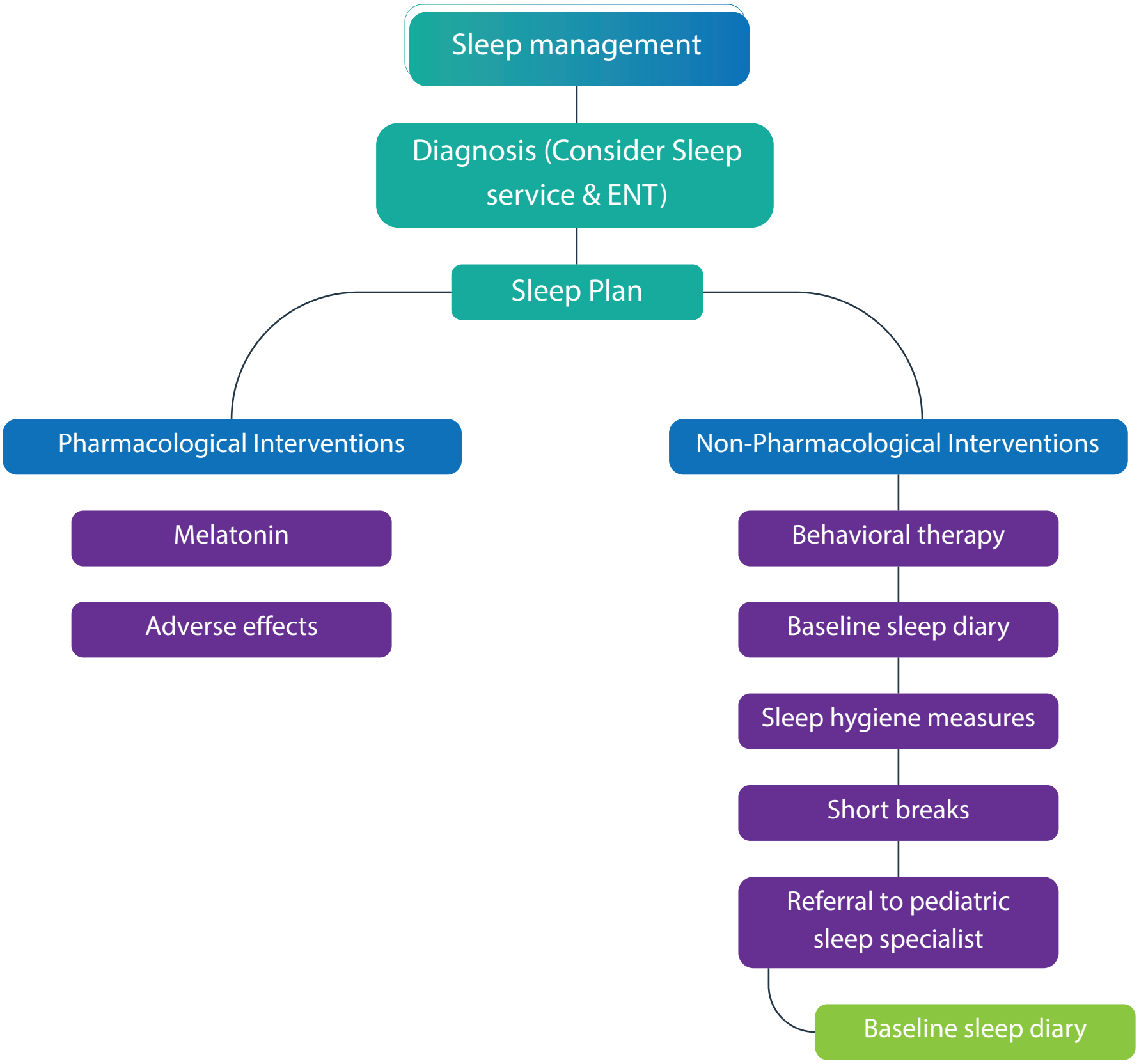

Behavior that challenges in autistic people under 19

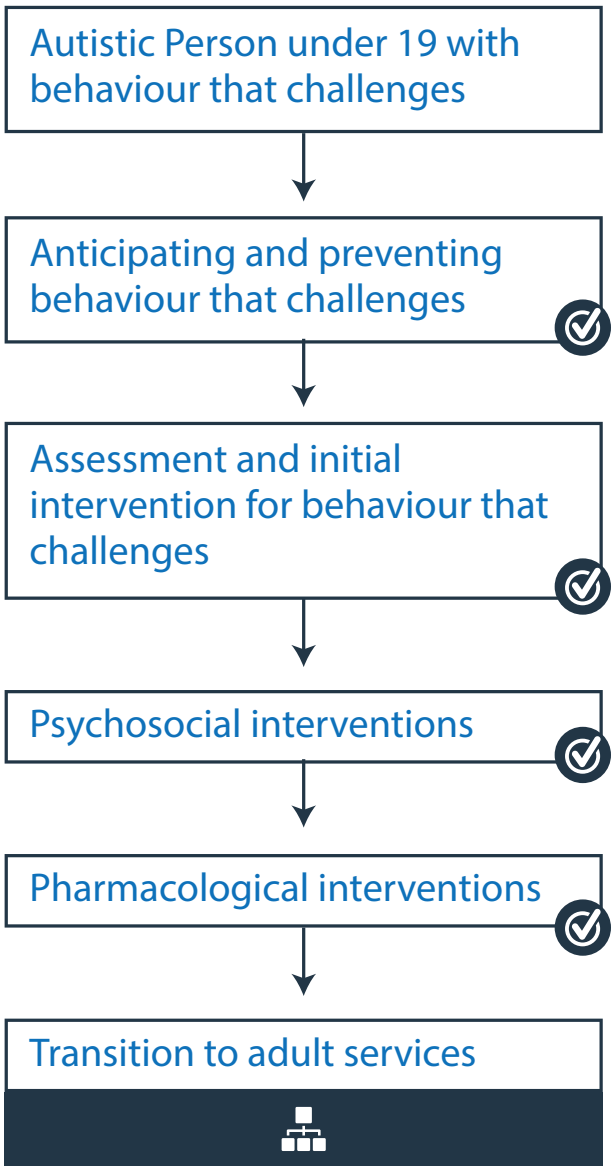

NICE Signs and Symptoms Tables: 13

[1] Table - Signs and symptoms of possible autism in Preschool School children (or equivalent mental age) 13

(Use this table to alert you to the possibility of autism if concerns have been raised).

The signs and symptoms in this table are a combination of delay in expected features of development and the presence of unusual features, and are intended to alert professionals to the possibility of autism in a child or young person about whom concerns have been raised. It is not intended to be used alone, but to help professionals recognize a pattern of impairments in reciprocal social and communication skills, together with unusual restricted and repetitive behaviors.

Social interaction and reciprocal communication behaviours

Spoken language:

- Language delay (in babble or words, for example less than ten words by the age of 2 years)
- Regression in or loss of use of speech
- Spoken language (if present) may include unusual:
  - non-speech like vocalisations.
  - odd or flat intonation.
  - frequent repetition of set words and phrases ('echolalia').
  - reference to self by name or 'you' or 'she/he' beyond 3 years.
- Reduced and/or infrequent use of language for communication, for example use of single words although able to speak in sentences.

Responding to others:

- Absent or delayed response to name being called, despite normal hearing.
- Reduced or absent responsive social smiling.
- Reduced or absent responsiveness to other people's facial expressions or feelings.
- Unusually negative response to the requests of others (demand avoidant behaviour).
- Rejection of cuddles initiated by parent or carer, although may initiate cuddles themselves.

Interacting with others:

- Reduced or absent awareness of personal space, or unusually intolerant of people entering their personal space.
- Reduced or absent social interest in others, including children of his/her own age – may reject others; if interested in others, may approach others inappropriately, seeming to be aggressive or disruptive.
- Reduced or absent imitation of others' actions.
- Reduced or absent initiation of social play with others, plays alone.
- Reduced or absent enjoyment of situations that most children like, for example, birthday parties.
- Reduced or absent sharing of enjoyment.

Eye contact, pointing and other gestures:

- Reduced or absent use of gestures and facial expressions to communicate (although may place adult's hand on objects)
- Reduced and poorly integrated gestures, facial expressions, body orientation, eye contact (looking at people's eyes when speaking) and speech used in social communication
- Reduced or absent social use of eye contact, assuming adequate vision
- Reduced or absent joint attention shown by lack of:
    - gaze switching.
    - following a point (looking where the other person points to – may look at hand) .
    - using pointing at or showing objects to share interest.

Ideas and imagination:

Reduced or absent imagination and variety of pretend play

Unusual or restricted interests and/or rigid and repetitive behaviours:

- Repetitive 'stereotypical' movements such as hand flapping, body rocking while standing, spinning, finger flicking.
- Repetitive or stereotyped play, for example opening and closing doors.
- Over-focused or unusual interests.
- Excessive insistence on following own agenda.
- Extremes of emotional reactivity to change or new situations, insistence on things being 'the same'.
- Over or under reaction to sensory stimuli, for example textures, sounds, smells.
- Excessive reaction to taste, smell, texture or appearance of food or extreme food fads.

NICE Signs and Symptoms Tables: 13

[2] Table – Signs and symptoms of possible autism in Primary School children (age 11–5 years or equivalent mental age) 13

(Use this table to alert you to the possibility of autism if concerns have been raised)

The signs and symptoms in this table are a combination of delay in expected features of development and the presence of unusual features, and are intended to alert professionals to the possibility of autism in a child or young person about whom concerns have been raised. It is not intended to be used alone, but to help professionals recognize a pattern of impairments in reciprocal social and communication skills, together with unusual restricted and repetitive behaviors.

Social interaction and reciprocal communication behaviors

Spoken language:

- Spoken language may be unusual in several ways:
  - very limited use.
  - monotonous tone.
  - repetitive speech, frequent use of stereotyped (learnt) phrases, content dominated by excessive information on topics of own interest.
  - talking 'at' others rather than sharing a two-way conversation.
  - responses to others can seem rude or inappropriate.

Responding to others:

- Reduced or absent response to other people's facial expression or feelings.
- Reduced or delayed response to name being called, despite normal hearing.
- Subtle difficulties in understanding other's intentions; may take things literally and misunderstand sarcasm or metaphor.
- Unusually negative response to the requests of others (demand avoidant behavior).

Interacting with others:

- Reduced or absent awareness of personal space, or unusually intolerant of people entering their personal space.
- Reduced or absent social interest in people, including children of his/her own age – may reject others; if interested in others, may approach others inappropriately, seeming to be aggressive or disruptive.
- Reduced or absent greeting and farewell behaviours.
- Reduced or absent awareness of socially expected behaviour .
- Reduced or absent ability to share in the social play or ideas of others, plays alone.
- Unable to adapt style of communication to social situations, for example may be overly formal or inappropriately familiar.
- Reduced or absent enjoyment of situations that most children like.

Social interaction and reciprocal communication behaviors

Eye contact, pointing and other gestures:

- Reduced and poorly integrated gestures, facial expressions and body orientation, eye contact (looking at people's eyes when speaking), and speech used in social communication.
- Reduced or absent social use of eye contact, assuming adequate vision.
- Reduced or absent joint attention shown by lack of:
  - gaze switching.
  - following a point (looking where the other person points to – may look at hand).
  - using pointing at or showing objects to share interest.

Ideas and imagination:

- Reduced or absent flexible imaginative play or creativity, although scenes seen on visual media (for example television) may be re-enacted.
- Makes comments without awareness of social niceties or hierarchies.

Unusual or restricted interests and/or rigid and repetitive behaviors:

- Repetitive 'stereotypical' movements such as hand flapping, body rocking while standing, spinning, finger flicking.
- Play repetitive and oriented towards objects rather than people.
- Over-focused or unusual interests.
- Rigid expectation that other children should adhere to rules of play.
- Excessive insistence on following own agenda.
- Extremes of emotional reactivity that are excessive for the circumstances .
- Strong preferences for familiar routines and things being 'just right'.
- Dislike of change, which often leads to anxiety or other forms of distress (including aggression).
- Over or under reaction to sensory stimuli, for example textures, sounds, smells.
- Excessive reaction to taste, smell, texture or appearance of food or extreme food fads.

Other factors that may support a concern about autism:

- Unusual profile of skills or deficits (for example, social or motor coordination skills poorly developed, while particular areas of knowledge, reading or vocabulary skills are advanced for chronological or mental age).
- Social and emotional development more immature than other areas of development, excessive trusting (naivety), lack of common sense, less independent than peers.

NICE Signs and Symptoms Tables: 13

[3] Table – Signs and symptoms of possible autism in Secondary School children (older than 11 years or equivalent mental age) 13

(Use this table to alert you to the possibility of autism if concerns have been raised)

The signs and symptoms in this table are a combination of delay in expected features of development and the presence of unusual features, and are intended to alert professionals to the possibility of autism in a child or young person about whom concerns have been raised. It is not intended to be used alone, but to help professionals recognise a pattern of impairments in reciprocal social and communication skills, together with unusual restricted and repetitive behaviours.

Social interaction and reciprocal communication behaviors

Spoken language:

- Spoken language may be unusual in several ways:
  - very limited use .
  - monotonous tone.
  - repetitive speech, frequent use of stereotyped (learnt) phrases, content dominated by excessive information on topics of own interest.
  - talking 'at' others rather than sharing a two-way conversation.
  - responses to others can seem rude or inappropriate.

Interacting with others:

- Reduced or absent awareness of personal space, or unusually intolerant of people entering their personal space
- Long-standing difficulties in reciprocal social communication and interaction: few close friends or reciprocal relationships
- Reduced or absent understanding of friendship; often an unsuccessful desire to have friends (although may find it easier with adults or younger children)
- Social isolation and apparent preference for aloneness
- Reduced or absent greeting and farewell behaviors
- Lack of awareness and understanding of socially expected behavior
- Problems losing at games, turn-taking and understanding 'changing the rules'
- May appear unaware or uninterested in what other young people his or her age are interested in
- Unable to adapt style of communication to social situations, for example may be overly formal or inappropriately familiar
- Subtle difficulties in understanding other's intentions; may take things literally and misunderstand sarcasm or metaphor
- Makes comments without awareness of social niceties or hierarchies
- Unusually negative response to the requests of others (demand avoidant behavior).

Eye contact, pointing and other gestures:

- Poorly integrated gestures, facial expressions, body orientation, eye contact (looking at people's eyes when speaking) assuming adequate vision, and spoken language used in social communication

Ideas and imagination:

- History of a lack of flexible social imaginative play and creativity, although scenes seen on visual media (for example, television) may be re-enactedIdeas and imagination.

Unusual or restricted interests and/or rigid and repetitive behaviors:

- Repetitive 'stereotypical' movements such as hand flapping, body rocking while standing, spinning, finger flicking.
- Preference for highly specific interests or hobbies.
- A strong adherence to rules or fairness that leads to argument.
- Highly repetitive behaviors or rituals that negatively affect the young person's daily activities
- Excessive emotional distress at what seems trivial to others, for example change in routine.
- Dislike of change, which often leads to anxiety or other forms of distress including aggression.
- Over or under reaction to sensory stimuli, for example textures, sounds, smells.
- Excessive reaction to taste, smell, texture or appearance of food and/or extreme food fads.

Other factors that may support a concern about autism:

- Unusual profile of skills and deficits (for example, social or motor coordination skills poorly developed, while particular areas of knowledge, reading or vocabulary skills are advanced for chronological or mental age).
- Social and emotional development more immature than other areas of development, excessive trusting (naivety), lack of common sense, less independent than peers.

NICE Quality Measures (8 Key Performance Indicators)

https://www.nice.org.uk/guidance/qs51

Quality statement 1: Diagnostic assessment by an autism team

Quality statement 2: Assessment and diagnosis

Quality statement 3: Personalised plan

Quality statement 4: Coordination of care and support

Quality statement 5: Treating the core features of autism: psychosocial interventions

Quality statement 6: Treating the core features of autism: medication

Quality statement 7: Assessing possible triggers for behaviour that challenges

Quality statement 8: Interventions for behaviour that challenges

(For details of the KPI equations and data sources, refer to the NICE website)

Autism Multidisciplinary Team (ASD-MDT) members and qualifications

Composition

- Physician (child psychiatrist, developmental pediatrician, pediatric neurologist who is trained on autism).
- Speech therapist.
- Occupational therapist.
- Clinical psychologist .

Qualifications

- refer to the standards of CBAHI Autism service certification program: https://portal.cbahi.gov.sa/english/cbahi-standards

Roles and responsibilities

- It is recommended that all clinicians involved in assessment of ASD concerns obtain relevant training and expertise covering all the following areas:
  - Developmental assessment (typical/atypical)
  - Neurodevelopmental disorders clinical presentation.
  - Signs and symptoms associated with common co-occurring or differential diagnosis conditions.
  - The criteria for ASD and co-occurring or differential diagnosis conditions described by the current version of international diagnostic manuals (e.g. DSM and/or ICD).
  - The impact of other important considerations, such as intellectual and/or communication capacity, culturally, linguistically and/or socio-economically diverse background.
  - Clinical reasoning in weighing evidence, integrating findings and reaching assessment conclusions.
  - Communicating with individuals on the autism spectrum and their caregivers.

ASD Referral Form (Components)

- Antenatal and perinatal history
- Developmental milestones
- Factors associated with an increased prevalence of autism (See Table below)
- Relevant medical history and investigations
- Information from previous assessments.

Table – Factors associated with an increased prevalence of ASD

- A sibling with autism.
- Birth defects associated with central nervous system malformation and/or dysfunction, including cerebral palsy.
- Gestational age less than 35 weeks.
- Parental schizophrenia-like psychosis or affective disorder.
- Maternal use of sodium valproate in pregnancy.
- A learning (intellectual) disability.
- Attention deficit hyperactivity disorder.
- Neonatal encephalopathy or epileptic encephalopathy, including infantile spasms.
- Chromosomal disorders such as Down's syndrome.
- Genetic disorders such as fragile X.
- Muscular dystrophy.
- Neurofibromatosis.
- Tuberous sclerosis.

ASD Screening Tools

- Modified Checklist for Autism in Toddlers (M-CHAT)
- Checklist for autism in toddlers (CHAT)
- Social communication questionnaire (SCQ)
- Early screening for autistic traits (ESAT)
- Childhood autism spectrum test (CAST)
- Screening tool for autism in toddlers (STAT)
- Autism spectrum screening questionnaire (ASSQ)
- The Survey of Well-being of Young Children (SWYC)

	Tool	Validation KSA	Age group	Arabic version availability
1	M-CHAT	Validated	16 – 30 months	Yes
2	CHAT	Validated	18 – 24 months	Yes
3	SCQ	Validated	Over 4 Y with mental age > 2 years	No
4	ESAT	No	0 – 36 months	No
5	CAST	No	4 – 11 years	No
6	STAT	No	24 – 36 months	No
7	ASSQ	No	6 – 17 years	No
8	SWYC	No	2 – 60 months	Yes

Useful Links and Resources

Modified Checklist for Autism in Toddlers, Revised (M-CHAT-R)	
Age group	30 - 16 months of age
English sources	https://mchatscreen.com/<br>https://www.autismspeaks.org/screen-your-child
Arabic version	https://mchatscreen.com/wp-content/uploads/05/2015/M-CHAT-R_F_Arabic.pdf
Arabic version with pictures	https://secureservercdn.net/545/104.238.68.130.e42.myftpupload.com/wp-content/uploads/05/2016/M-CHAT-R_F_Arabic_v2.pdf

Checklist for autism in toddlers (CHAT)	
Age group	18 – 24 months
English sources	https://www.autismresearchcentre.com/tests/checklist-for-autism-in-toddlers-chat/
Arabic version	https://docs.autismresearchcentre.com/tests/CHAT_Arabic.pdf

Social Communication Questionnaire (SCQ)	
Age group	Over 4 years with mental age over 2 years
English sources	https://www.wpspublish.com/scq-social-communication-questionnaire
Arabic version	Not available

Early Screening of Autistic Traits (ESAT)	
Age group	0 – 36 months of age
English sources	http://disabilitymeasures.org/ESAT/
Arabic version	Not available

Childhood Autism Spectrum Test (CAST)	
Age group	4 – 11 years
English sources	https://psychology-tools.com/test/cast
Arabic version	Not available

Screening tool for autism in toddlers (STAT)	
Age group	24 – 36 months
English sources	http://stat.vueinnovations.com/about
Arabic version	Not available

Autism spectrum screening questionnaire (ASSQ)	
Age group	6 – 17 years
English sources	https://www.gu.se/en/gnc/gncs-resources/screening-questionnaires/assq-autism-spectrum-screening-questionnaire#About-the-ASSQ<br>https://www.gu.se/en/gnc/gncs-resources/screening-questionnaires-and-protocols/assq-autism-spectrum-screening-questionnaire<br>https://psychology-tools.com/test/autism-spectrum-screening-questionnaire
Arabic version	Not available

The Survey of Well-being of Young Children (SWYC)	
Age group	2 – 60 months
English sources	https://pediatrics.tuftsmedicalcenter.org/the-survey-of-wellbeing-of-young-children/overview
Arabic version	https://www.tuftschildrenshospital.org/The-Survey-of-Wellbeing-of-Young-Children/Translations/Arabic-SWYC

Ages & Stages Questionnaire Social/Emotional 2 (ASQSE 2)	
Age group	2 – 60 months
English sources	https://agesandstages.com/products-pricing/asqse-2/
Arabic version	Not available

CBAHI ASD certification program (for autism centers)

32 standards https://portal.cbahi.gov.sa/english/cbahi-standards

National ASD Registry

(a national project under development)

ASD service providers in KSA

Can be accessed through the official websites of the following organizations:-

The National Center for Developmental and Behavioral Disorders (NCBDC), Saudi Health Council (SHC)

https://shc.gov.sa/Arabic/NCDBD/servicecenterssite/Pages/default.aspx

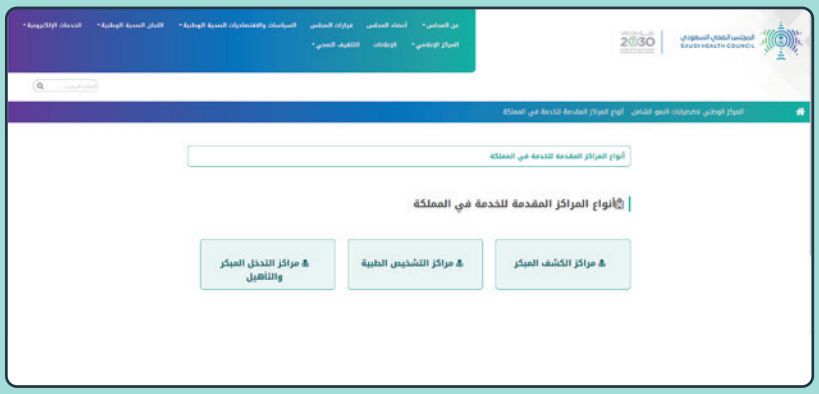

The Autism Center of Excellence (ACE):-

https://acesaudi.org/center/

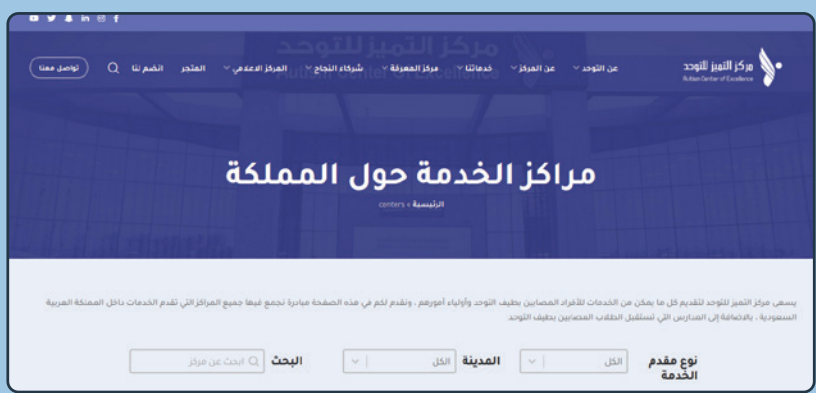

Patient and Family information in Arabic

اضطراب طيف التوحد	85
التشخيص	85
العلاج	85
إدارة الحالات الطبية والنفسية الآخرى	86
التخطيط للمستقبل	87

اضطراب طيف التوحد

التشخيص

سيقيم طبيب العائلة طفلكم بحثاً عن علامات تأخر نمائي في أثناء زيارات الفحص المنتظمة. إذا اعترى طفلك أي من أعراض اضطراب طيف التوحد، فستتم إحالتك على الأرجح إلى مختص يعالج أطفال اضطراب طيف التوحد، كطبيب نفسي للأطفال، أو أخصائي نفسي، أو طبيب أعصاب أطفال، أو طبيب أطفال متخصص في النمو؛ وذلك للتقييم.

قد يكون من الصعب الوصول إلى تشخيص؛ نظرًا للتنوع الكبير في أعراض اضطراب طيف التوحد وشدته. ولا يوجد اختبار طبي معين لتحديد الإصابة بهذا الاضطراب. فعوضًا عن ذلك، قد يقوم المختص بأي مما يلي:

- ملاحظة الطفل، والسؤال عن الكيفية التي تطورت على نحوها تفاعلات الطفل الاجتماعية، ومهاراته التواصلية، وسلوكه، وكيف تغيرت كل منها بمرور الوقت.
- إجراء اختبارات لطفلك، تتناول السمع، والتخاطب، واللغة، والمستوى النمائي، والأمور الاجتماعية والسلوكية.
- تقديم تفاعلات اجتماعية وتواصلية ذات بنية محددة لطفلك، وحساب نتيجة أدائه فيها.
- تطبيق المعايير التشخيصية كما في الدليل التشخيصي والإحصائي للاضطرابات العقلية بنسخته الخامسة، الذي نشرته جمعية الطب النفسي الأمريكية أو التصنيف الدولي للأمراض، المراجعة العاشرة (النسخة الأسترالية) حسب التوصيات المحلية.
- إشراك أخصائيين آخرين في تحديد التشخيص.
- التوصية بإجراء اختبار جيني للتعرف على ما إذا كان طفلك مصابًا باضطراب جيني، كمتلازمة ريت، أو متلازمة X الهش.

العـلاج

لا يوجد علاج شافٍ بعد لاضطراب طيف التوحد، وليست هناك طريقة علاج واحدة تناسب جميع الحالات. الهدف من الخطة العلاجية هو زيادة قدرة الطفل على أداء الأعمال بأكبر قدر ممكن من خلال الحد من أعراض اضطراب طيف التوحد ودعم النمو والتعلم لديه. يمكن للتدخل المبكر خلال سنوات ما قبل المدرسة أن يساعد طفلك على تعلم المهارات الاجتماعية والوظيفية والسلوكية الحيوية ومهارات التواصل.

وقد تساعد مجموعة من طرق العلاج والتدخلات المنزلية والمدرسية في علاج اضطراب طيف التوحد، كما قد تتغير احتياجات طفلك بمرور الوقت. يمكن لمقدم الرعاية الصحية أن يوصي بخيارات ويساعدك على التعرف على الموارد في منطقتك.

إذا تم تشخيص طفلك باضطراب طيف التوحد، تحدث إلى الخبراء بشأن وضع استراتيجية للعلاج وتكوين فريق من المتخصصين لتلبية احتياجات طفلك.

قد تشمل خيارات العلاج:

العلاجات السلوكية والاتصالية: تعالج العديد من البرامج مجموعة من الصعوبات الاجتماعية واللغوية والسلوكية المرتبطة باضطراب طيف التوحد. وتركز بعض البرامج على الحد من السلوكيات المثيرة للمشاكل، وتعليم مهارات جديدة. وتركز البرامج الأخرى على تعليم الأطفال كيفية التصرف في المواقف الاجتماعية أو التواصل بشكل أفضل مع الآخرين. كذلك يمكن أن يساعد تحليل السلوك التطبيقي (ABA) الأطفال على تعلم مهارات جديدة وتعميم هذه المهارات في حالات متعددة من خلال نظام التحفيز القائم على المكافآت.

العلاجات التربوية: غالبًا ما يستجيب الأطفال المصابون باضطراب طيف التوحد جيدًا للبرامج التربوية التي تتميز بدرجة عالية من التنظيم. وتتضمن البرامج الناجحة عادةً فريقًا من الاختصاصيين، ومجموعة متنوعة من الأنشطة لتحسين المهارات الاجتماعية ومهارات الاتصال والسلوك. وغالبًا ما يظهر الأطفال قبل سن المدرسة ممن يحظون بتدخلات سلوكية فردية مركزة تقدمًا جيدًا.

العلاج الأسري: يمكن أن يتعلم الآباء وأفراد الأسرة الآخرون كيفية اللعب والتفاعل مع أطفالهم المرضى بطرق تحفز المهارات الاجتماعية وتعالج المشكلات السلوكية وتعلمهم مهارات الحياة اليومية والتواصل.

العلاجات الأخرى: بناء على احتياجات طفلك، فإن علاج النطق لتحسن مهارات التواصل، والعلاج المهني لتعليم أنشطة الحياة اليومية، والعلاج الطبيعي لتحسين الحركة والتوازن قد يكون مفيدًا. وقد يوصي الطبيب النفسي باتباع طرق لعلاج مشاكل السلوك.

الأدوية للاضطرابات المصاحبة: ليس هناك أي دواء في إمكانه تحسين العلامات الأساسية لاضطراب طيف التوحد، ولكن هناك أدوية معينة تساعد في السيطرة على الأعراض. فعلى سبيل المثال، قد توصف بعض الأدوية لطفلك في حال كان يعاني من فرط النشاط؛ تستخدم الأدوية المضادة للذهان أحيانًا في علاج المشكلات السلوكية الحادة؛ كما قد توصف مضادات الاكتئاب لعلاج القلق. أطلع مقدمي الرعاية الصحية بشأن أي دواء أو مكمل غذائي يتناوله الطفل أولاً بأول. ففي بعض الأحيان، قد تتفاعل الأدوية مع المكملات الغذائية، وتُسبب آثارًا جانبية خطيرة.

إدارة الحالات الطبية والنفسية الأخرى

بالإضافة إلى اضطراب طيف التوحد، يمكن أن يعاني الأطفال والمراهقون والبالغون الآتي:

مشاكل الصحة النفسية: قد يصاب الأطفال المصابون باضطراب طيف التوحد أيضًا بمشاكل طبية، مثل الصرع واضطرابات النوم أو تفضيلات غذائية محدودة أو مشاكل في المعدة. اسأل طبيب طفلك عن كيفية تحسين إدارة هذه الحالات معًا.

مشاكل مع الانتقال إلى مرحلة البلوغ: قد يواجه المراهقون والشباب الذين يعانون اضطراب طيف التوحد صعوبة في فهم تغيرات الجسم. كما أن الأوضاع الاجتماعية تزداد تعقيدا في مرحلة المراهقة، وقد يكون هناك قدر أقل من التسامح إزاء الاختلافات الفردية. مشاكل السلوك قد تكون صعبة خلال سنوات المراهقة.

اضطرابات الصحة النفسية الأخرى: غالبًا ما يعاني المراهقون والبالغون المصابون باضطراب طيف التوحد اضطرابات نفسية أخرى، مثل القلق والاكتئاب. يمكن أن يقدم طبيبك وأخصائي الصحة النفسية ومنظمات مناصرة للمرضى مجتمعية وخدمية المساعدة.

التخطيط للمستقبل

عادة ما يستمر الأطفال المصابون باضطراب طيف التوحد في التعلم والتعويض عن المشكلات طوال الحياة، ولكن معظمهم سيظل بحاجة إلى مستوى معين من الدعم. يمكن أن يجعل التخطيط لفرص طفلك المستقبلية، مثل التوظيف والكلية ووضع المعيشة والاستقلال والخدمات المطلوبة للدعم هذه العملية أكثر سلاسة.

https://www.mayoclinic.org/ar/diseases-conditions/autism-spectrum-disorder/diagnosis-treatment/drc-20352934

كما يوفر موقع موسوعة الملك عبد الله العربية للمحتوى الصحي معلومات صحية موثوقة باللغة العربية عن التوحد:

حقائق عن التوحد

دليل الوالدين للتعامل مع مريض التوحد

Plan for Scheduled Review and Update

Plan for Scheduled Review and Update

The CPG Adaptation Group decided to review this adapted CPG for updates after two years from its publication date (2023) which should be on (2026) after checking for updates in the source guidelines, consultation of expert opinion on the changes needed for updating according to the newest evidence and recommendations published in this area and the clinical audit and feedback from implementation efforts in the Saudi Arabian setting.

List of Funding Sources

List of Funding Sources

The funding body of this CPG was the National Center for Developmental and Behavioral Disorders, Saudi Health Council that is an official governmental organization and it has not influenced the formulation of the final evidence-based recommendations.

Moreover, this CPG adaptation project was not related to any pharmaceutical, industrial, or any other commercial entity.

References

1 American Psychiatric Association. Autism spectrum disorder. In: Diagnostic and Statistical Manual of Mental Disorders. 5th ed. American Psychiatric Association: Arlington, VA, 2013; p. 50.

2 Faras, H., Al Ateeqi, N., Tidmarshb, L. Autism spectrum disorders. Ann Saudi Med. ;2010 300-295 :(4)30 DOI: 4947.65261-0256/10.4103

3 Hassan, A. (2019). Arab views on autism. Encyclopedia of Autism Spectrum Disorders. Springer, New York, NY, 305-302 ;2019. https://doi.org/10.1007/978-3-319-91280-6_102309

4 Yazbak FE. Autism seems to be increasing worldwide, if not in London. BMJ. 2004 Jan 7-226:(7433)328;24. doi: 10.1136/bmj.328.7433.226-c. PMID: 14739196; PMCID: PMC318519.

5 Salhia HO, Al-Nasser LA, Taher LS, Al-Khathaami AM, El-Metwally AA. Systemic review of the epidemiology of autism in Arab Gulf countries. Neurosciences (Riyadh). 2014 Oct;6-291:(4)19. PMID: 25274588; PMCID: PMC4727667.

6 Mostafa, A. Addressing autism in the Arab world. Nature Middle East. 147;2011. doi:10.1038/nmiddleeast.2011.147.

7 Alnemary, F.M., Aldhalaan, H.M., Simon-Cereijido, G. and Alnemary, F.M. Services for children with autism in the Kingdom of Saudi Arabia. Autism. 602-592 :(5)21 ;2017. https://doi.org/10.1177/1362361316664868

8 Khan, A.S., AlGhadeer, H.A., Mohammed, A., Al-Qassimi, T.M.A.J., Al-Momen, H.H. and Al-Nazzal, M.Y. Autism in Saudi Arabia, a challenge to Saudi families: a cross-sectional study. International Journal of Medicine in Developing Countries. 1458–1453:(9)4 ;2020. https://doi.org/10.24911/IJMDC.51-1595277794

9 Towle, P. O., Patrick, P. A., Ridgard, T., Pham, S., & Marrus, J. Is earlier better? The relationship between age when starting early intervention and outcomes for children with autism spectrum disorder: a selective review. Autism Research and Treatment. 2020. https://doi.org/10.1155/2020/7605876.

10 Almandil, N.B., Alkuroud, D.N., AbdulAzeez, S., AlSulaiman, A., Elaissari, A. and Borgio, J.F. Environmental and Genetic Factors in Autism Spectrum Disorders: Special Emphasis on Data from Arabian Studies. Int. J. Environ. Res. Public Health. 658 ,16 ;2019; doi:10.3390/ijerph16040658.

11 Autism CRC The University of Queensland. 2018. Australian National Guideline for the Assessment and Diagnosis of Autism Spectrum Disorders | Autism CRC. [online] Available at: https://www.autismcrc.com.au/access/national-guideline [Accessed 1 December 2021].

12 Ministry of Health New Zealand (NZ). 2016. New Zealand Autism Spectrum Disorder Guideline. [online] Available at: https://www.health.govt.nz/our-work/disability-services/autism-spectrum-disorder-guideline [Accessed 1 December 2021].

13 National Institute for Health and Care Excellence (NICE). 2017. Autism spectrum disorder in under 19s: recognition, referral and diagnosis | Guidance | NICE. [online] Available at: https://www.nice.org.uk/guidance/cg128 [Accessed 1 December 2021].

14 Scottish Intercollegiate Guidelines Network (SIGN) Healthcare Improvement Scotland (HIS). 2016. Assessment, diagnosis and interventions for autism spectrum disorders. [online] Available at: https://www.sign.ac.uk/our-guidelines/assessment-diagnosis-and-interventions-for-autism-spectrum-disorders/ [Accessed 1 December 2021].

15 Amer YS, Wahabi HA, Abou Elkheir MM, et al. Adapting evidence-based clinical practice guidelines at university teaching hospitals: A model for the Eastern Mediterranean Region. J Eval Clin Pract. 11–1;2018. https://doi.org/10.1111/jep.12927

16 Amer YS, Elzalabany MM, Omar TI, Ibrahim AG, Dowidar NL. The 'Adapted ADAPTE': an approach to improve utilization of the ADAPTE guideline adaptation resource toolkit in the Alexandria Center for Evidence-Based Clinical Practice Guidelines. J Eval Clin Pract. 2015 Dec;106-1095:(6)21. doi: 10.1111/jep.12479

17 Fervers B, Burgers JS, Voellinger R, Brouwers M, Browman GP, Graham ID, Harrison MB, Latreille J, Mlika-Cabane N, Paquet L, Zitzelsberger L. Guideline adaptation: an approach to enhance efficiency in guideline development and improve utilisation. BMJ quality & safety. 2011 Mar 36-228:(3)20;1. https://doi.org/10.1136/bmjqs.2010.043257

18 DynaMed [Internet]. Ipswich (MA): EBSCO Information Services. 1995 - . Record No. T113665, Autism Spectrum Disorders; [updated 2018 Dec 03, cited 2021-11-29]. Available from https://www.dynamed.com/topics/dmp~AN~T113665 [Accessed 1 December 2021] (Registration and login required).

19 Hyman SL, Levy SE, Myers SM; Council On Children with Disabilities, Section On Developmental and Behavioral Pediatrics. Identification, Evaluation, and Management of Children with Autism Spectrum Disorder. Pediatrics. 2020 Jan;(1)145:e20193447. doi: 10.1542/peds.3447-2019. Epub 2019 Dec 16. PMID: 31843864.

20 Amer YS, Alenezi S, Bashiri FA, Alawami AH, Alhazmi AS, Aladamawi SA, et al. AGREEing on Clinical Practice Guidelines for Autism Spectrum Disorders in Children: A Systematic Review and Quality Assessment. Children 9:1050;2022. https://doi.org/10.3390/children9071050

21 Johnston A, Kelly SE, Hsieh SC, Skidmore B, Wells GA. Systematic reviews of clinical practice guidelines: a methodological guide. Journal of clinical epidemiology. 2019 Apr 76-108:64;1. https://doi.org/10.1016/j.jclinepi.2018.11.030

22 Brouwers MC, Spithoff K, Lavis J, Kho ME, Makarski J, Florez ID. What to do with all the AGREEs? The AGREE portfolio of tools to support the guideline enterprise. Journal of Clinical Epidemiology. 2020 Sep 125:191;1. https://doi.org/10.1016/j.jclinepi.2020.05.025

23 Agreetrust.org. 2021. The Appraisal of Guidelines for Research and Evaluation (AGREE) Enterprise website. [online] Available at: <https://www.agreetrust.org/> [Accessed 14 September 2021].

Appendices

- Appendix 1. List of the websites and databases we searched 97
- Appendix 2. Search strategy 98
- Appendix 3. PRISMA 2020 flow diagram 99
- Appendix 4. Adaptation Process Methodology 100

Appendix 1 | List of the websites and databases we searched:

We conducted a systemat.

CPG databases and libraries:

- Guidelines International Network (GIN) International Guidelines Library
- ECRI Guidelines Trust (USA)
- National Institute for Health and Care Excellence (NICE) UK
- Scottish Intercollegiate Guidelines Network (SIGN) UK.
- EBSCO DynaMed (USA) (subscription required)

Bibliographic databases:

- PubMed/ MEDLINE
- Google Scholar

Specialized professional societies:

- American Psychiatry Association (APA1)
- American Academy of Pediatrics (AAP)
- American Academy of Child and Adolescent Psychiatry (AACAP)
- Canadian Academy of Child & Adolescent Psychiatry (CACAP)
- European Society for Child and Adolescent Psychiatry (ESCAP)
- International Association for Child and Adolescent Psychiatry and Allied Professions (IACAPAP)
- The British Psychological Society (BPS)
- American Psychological Association (APA2)
- American Speech-Language-Hearing Association (ASHA)
- Australian Psychological Society (APS)
- American Occupational Therapy Association (AOTA).
- Australian Psychology Society (APS)

Appendix 2| Search strategy

Keywords: autism, autism spectrum disorder, ASD, autistic disorder, infantile autism, childhood autism, pervasive developmental disorder (PDD), Asperger's syndrome, and PDD not otherwise specified (PDD-NOS), pediatrics, pediatric medicine, child health, mental health, treatment, management, pharmacology, practice guidelines, clinical practice guidelines, healthcare quality, patient safety, evidence-based medicine, AGREE II instrument, quality assessment, critical appraisal, evidence-based pediatrics, evidence-based psychiatry.

Search strategy (MEDLINE):

(autism[Title/Abstract]) OR (autism spectrum disorder [Title/Abstract]) OR (ASD[Title/Abstract]) OR (autistic disorder [Title/Abstract]) OR (infantile autism [Title/Abstract]) OR (childhood autism [Title/Abstract]) OR (pervasive developmental disorder [Title/Abstract]) OR (PDD[Title/Abstract]) OR (Asperger's syndrome [Title/Abstract]) OR (PDD[Title/Abstract] NOT otherwise specified [Title/Abstract]) OR (PDD-NOS [Title/Abstract]) AND (pediatrics) OR (pediatric medicine) OR (child health) OR (mental health) AND (practice guidelines) OR (clinical practice guidelines) OR (evidence-based medicine). MY NCBI Filters: Article Type: Practice Guideline. Publication date: From 2015/10/1 to 2022/07/01.

Total number of retrieved articles	Total number of excluded articles	Total number of included CPGs
165	161	4

Appendix 3 | PRISMA 2020 flow diagram for new systematic reviews which included searches of databases and registers only.

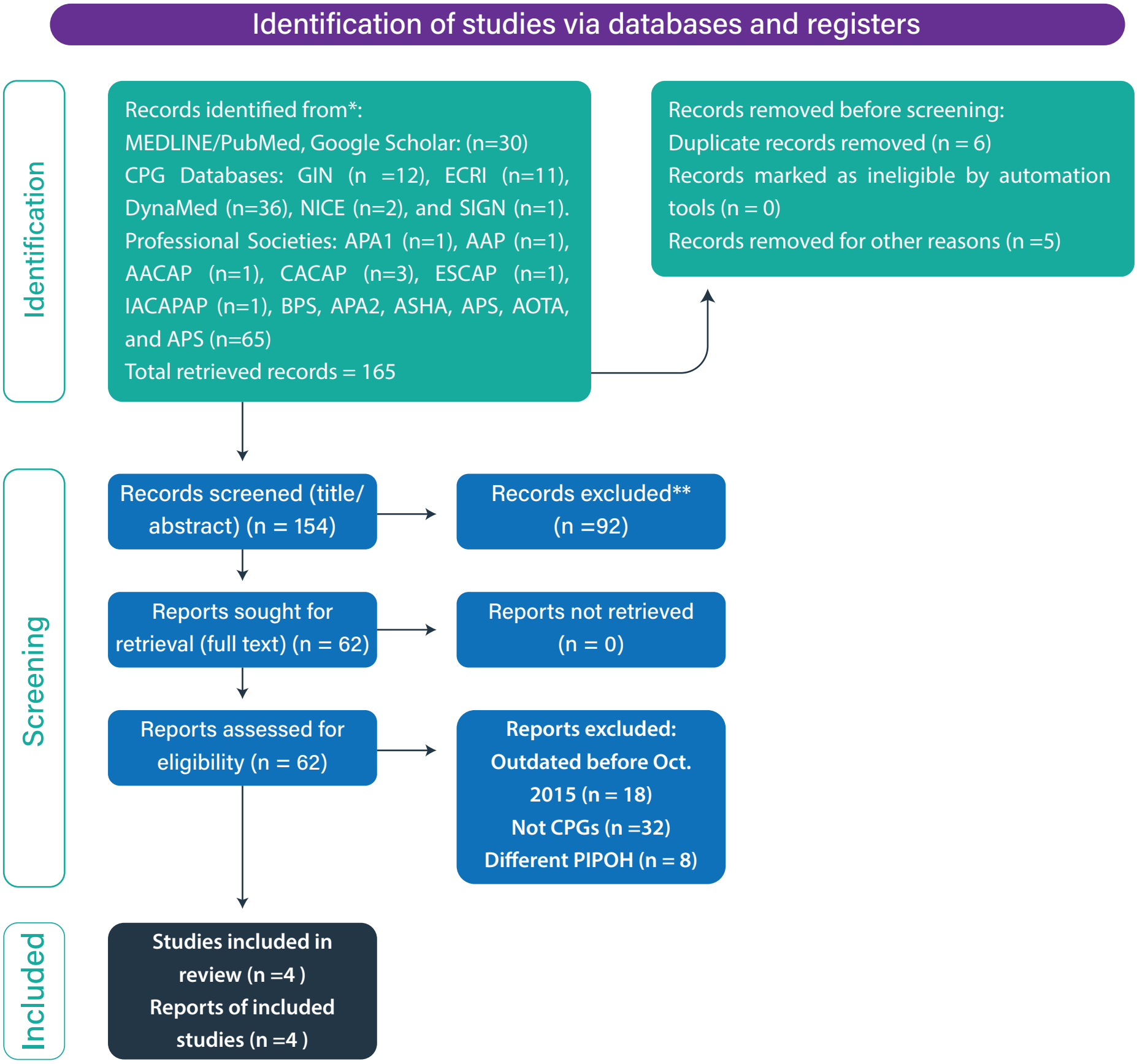

*Consider, if feasible to do so, reporting the number of records identified from each database or register searched (rather than the total number across all databases/registers).

**If automation tools were used, indicate how many records were excluded by a human and how many were excluded by automation tools.
From: Page MJ, McKenzie JE, Bossuyt PM, Boutron I, Hoffmann TC, Mulrow CD, et al. The PRISMA 2020 statement: an updated guideline for reporting systematic reviews. BMJ 372;2021:n71. doi: 10.1136/bmj.n71

For more information, visit: http://www.prisma-statement.org/

Appendix 4 | Adaptation Process Methodology

Phase One – Set-Up:

1. Checking the feasibility for CPG adaptation
Preliminary rapid search showed a large number of published CPGs addressing this topic.

2. Establishing a Guideline Adaptation Group (GAG)
The project lead was assigned by the SHC (SA) who was also nominated as the clinical chair (SA), and worked closely with the methodology chair (YSA), to define the initial scope of the CPG. A group of clinical and methodological experts was assigned from the network of the Saudi Health Council, National Center for Developmental and Behavioral Disorders to be the members of the GAG (full list provided in Table 1). The methodology followed was according to the 'KSU-Modified-ADAPTE' method.<br>Detailed composition of the GAG including clinical specialties and roles.<br>There was a comprehensive ASD stakeholder representation in the GAG and the ERG. The only limitation in the composition of these two groups was the lack of a specialized Health Economist with expertise in Health Technology Assessments or Appraisals (HTAs) (Economic Evidence). It was not feasible to recruit one as this is a rare specialty in the whole country and even region. Issues related to resources and cost were discussed within the GAG and ERG and considered during adaptation of the recommendations.

3. Selecting and prioritizing of the CPG health topic
The topic of ASD was identified by the NCDBD as one of the main high-priority topics for developmental and behavioral disorders in Saudi Arabia.

4. Identifying necessary resources and skills
The methodology chair conducted capacity building sessions for the GAG members on the CPG adaptation methodology, systematic review of CPGs, and evaluation of CPGs at the outset of the project in addition to hands on technical support throughout the process.

Appendix 4 | Adaptation Process Methodology

Phase One – Set-Up:

5. Completing the tasks of the set up phase
All members of the GAG declared their conflicts of interests and agreed on the terms of reference for this CPG adaptation project.<br>Members of both of the GAG and ERG had no conflicts of (or competing) interests to declare at the time of this CPG adaptation project

6. Writing up the CPG adaptation working plan
A timeline for the CPG adaptation project was outlined by the NCDBD-SHC and agreed upon by all GAG members.<br>Thirteen two-hour-meetings were conducted for the larger groups that included two face-to-face and 11 online meetings. Moreover, 10 parallel meetings were conducted by subgroups of the GAG to complete specific tasks within the CPG adaptation process (e.g., search for source CPGs, screening source CPGs, AGREE II assessment of CPGs, drafting the first version of the adapted recommendations, identifying and drafting the CPG implementation tools, and drafting the final version of the adapted CPG based on the external review input).

Appendix 4 | Adaptation Process Methodology

Phase Two – Adaptation:

7. Determining the health questions

Refer to Scope and purpose section. The PIPOH Model was used to identify the health questions that guided the search and screening process.

8. Searching for source CPGs and other relevant documents

A systematic review of evidence-based CPGs for ASD in children was conducted21. Its protocol was registered in the OSF (Open Science Foundation) (20).

9. Screening retrieved source CPGs

See PRISMA 2020 Flow chart (Appendix 3)

10. Reducing the large number of retrieved CPGs

The GAG relied on the inclusion and exclusion (eligibility) criteria for screening and filtration as showed in the PRISMA flowchart (Appendix 3).

Inclusion / Exclusion CPGs Selection Criteria

- **Methods of Development:** Evidence-Based CPGs: (Detailed Methodology of Development Documented; link Recommendations with Evidence; link to Systematic Reviews) rather than Consensus-based CPGs (Expert opinion)
- **Author(s) Organization (CPG development group) from** CPGs Database (Producer or Finder) and Specialized Society (clinical specialty) rather than single authors.
- **Country:** International or national CPGs.
- **Date of Publication:** Any CPG published after October 2015.
- **Language:** English and Arabic CPGs only.
- **Status:** Only original source CPG (de novo developed) rather than adapted CPGs.

A total of 165 records were retrieved, 161 were excluded according to the health questions and the eligibility criteria (for details refer to the PRISMA flowchart (Appendix 3)). Only four source original CPGs were found to be eligible for the quality assessment step. Two reviewers conducted the screening (AHA, SMA) and two additional reviewers (SMA, YSA) resolved any discrepancies by discussions.

Appendix Table 1 | List of retrieved eligible source CPGs

Organization, Country	Health System, Economic Classification	CPG Title	Year of publication
Australian Autism CRC (ACRC)	National Health Insurance, High-income country	Australian National Guideline for the Assessment and Diagnosis of Autism Spectrum Disorders	2018
Ministry of Health New Zealand (NZ)	National Health Insurance, High-income country	New Zealand Autism Spectrum Disorder Guideline	2016
National Institute for Health and Care Excellence (NICE) UK	National Health Service, High-income country	Autism spectrum disorder in 19s: recognition, under referral and diagnosis	2017
Scottish Intercollegiate Guidelines Network (SIGN), Healthcare Improvement Scotland (HIS)	National Health Service, High-income country	Assessment, diagnosis and interventions for autism spectrum disorders	2019

11. Assessment of the Quality of the Source CPGs using the AGREE II Instrument

The Appraisal of Guidelines Research & Evaluation (AGREE) II Instrument 22,23 was used to assess the quality of the four eligible Source CPGs using the online platform (MY AGREE PLUS) by four independent appraisers from the GAG including three clinicians (SAA, SA, MWB) and one CPG methodologist (YSA). Disagreements in the ratings of the AGREE II items were resolved by discussions (online meetings) (20).

Appendix Table 2 | AGREE II assessment results and the standardized domain scores for the four included CPGs

Source CPGs/ AGREE II Domains	ACRC 2018	NICE 2017	NZ 2016	SIGN 2019
D1: Scope and Purpose	99	96	93	97
D2: Stakeholder Involvement	99	72	71	79
D3: Rigour of Development	84	93	86	85
D4: Clarity and Presentation	93	97	94	93
D5: Applicability	92	89	54	85
D6: Editorial Independence	92	92	69	92
Overall assessment 1	92	96	88	92
Overall assessment 2 (Recommend for use)	Yes - 3, Yes with modifications - 1, No - 0	Yes - 2, Yes with modifications - 2, No - 0	Yes - 4, Yes with modifications - 0, No - 0	Yes - 2, Yes with modifications - 2, No - 0

12. Assessing the currency of the selected source CPG

Appendix Table 3 | Currency survey of the CPG developer

1. Are you aware of any new evidence relevant to this CPG statement? No.
2. Is there any new evidence to invalidate any of the recommendations comprising the CPG? No.
3. Are there any plans to update the CPG in the near future? No.
4. When the CPG was last updated? What is the citation for the latest version? (See References 11-14).

Steps from 15-13 were not conducted as the GAG relied on the results of the AGREE II assessment to reach the required assessments of CPG content, consistency, acceptability, and applicability.

Steps 16 and 17: the AGREE II assessments were discussed among the CRC 2018, MOH NZ 2016, NICE 2017, and SIGN-HIS 2016 CPGs and adapt all of its recommendations and implementation tools.

14. Step 18 Preparing the draft adapted CPG.

The first draft of the adapted CPG was prepared and sent for external review via email with the related clinical review and methodology review forms.

Phase Three – Finalization:

15. Step 19. External review (Clinical Content and Methodology)

Details of the members of the external review group.
The first draft of the adapted ASD CPG draft was sent to a national external review group (ERG) representing different healthcare sectors in Saudi Arabia. This ERG conducted clinical content and methodology reviews. The ERG clinical reviewers included a consultant psychoanalyst, child and adolescent psychiatrist, a consultant developmental and behavioral pediatrician, a consultant clinical neuropsychologist, a consultant occupational therapist, a consultant speech and language pathologist, a senior nurse academic, a senior clinical psychiatric pharmacist, and a medical technologist and ASD patient representative and advocate. Additionally, a senior expert guideline methodologist and academic was invited to review the CPGA methodology. The GAG reviewed the feedback received from the ERG and addressed their comments and suggestions in the final draft of the CPG.
ASD patients values and preferences were captured by inviting a patient representative (parent of an ASD child) as a member of the external review of the finalized CPG. The finalized ASD CPG was shared then a face-to-face meeting and focus group discussion was conducted at the Saudi Health Council among the other external reviewers for the clinical content. Several amendments were made based on that feedback.

16. Step 20. Consulting with endorsement bodies

The adapted CPG will be endorsed by the Saudi Health Council, the National Center for Developmental and Behavioral Disorders, Riyadh, Saudi Arabia.

17.Step 21. Consulting with the source CPG developer (s)

We have contacted the developer organizations of the four source CPGs (ACRC 2018, NZ MOH 2016, NICE 2017, and SIGN-HIS 2016) requesting their permission to use their CPGs in our National ASD CPG Adaptation Project:

- **SIGN-HIS:** Permission granted on 2021/12/3 (Correspondence: Roberta James, SIGN Programme Lead, Healthcare Improvement Scotland).
- **NZ MOH:** Permission granted on 2022/02/7 (Correspondence: Helen Hayes, SME for Autism within Ministry of Health Disability Directorate, Autism Guidelines Group for New Zealand Ministry of Health).
- **ACRC:** Permission granted on 2022/04/13 (Correspondence: Therese Conway, Research Program Manager, Autism CRC).
- **NICE:** Permission granted on 2022/04/25 (Correspondence: Iain Moir, Programme Manager: intellectual property, content and business management Science, Evidence and Analytics Directorate, National Institute for Health and Care Excellence).

Phase Three – Finalization:

18. Step 22. Acknowledging the source documents

The source CPG, relevant articles and websites have been clearly acknowledged in the adapted CPG document.

19. Step 23. Planning for aftercare of the adapted CPG

The GAG decided on a plan for reviewing and updating the adapted CPG using the CheckUp Tool that is freely provided by the AGREE Enterprise

20. Step 24. Producing the final adapted CPG document

This document represents the finalized adapted CPG full document. Table 4 presents the full map of the CPG adaptation process including the steps that were followed and the steps that were not followed with the relevant reasons.

The finalized adapted ASD-CPG was composed of a set of recommendations that were adapted (or sometimes adopted) from the four source high-quality CPGs as relevant to the Saudi Arabian healthcare system, the currently available ASD services and centers, and consideration of the available information of the latest updates of the 2030 vision health model transformation at the time of the finalization of this CPG project relevant to ASD care in Saudi Arabia.

The GAG was divided into four groups. Each group was assigned to draft the set of recommendations from one of the four source CPGs using a tabular template including a comprehensive list of options of care for the management of ASD that were identified during the PIPOH (step. This was done twice; firstly, without any modifications at all for the comparison table of the published SR of ASD CPGs and secondly, done for the adapted ASD-CPG that included the customization of some recommendation statements to be suitable for implementation in the current Saudi Arabia healthcare system and ASD care services.

This finalized set of adapted recommendations was reflected as well on the adoption, adaptation, and/ or development of relevant CPG Implementation Tools.

All of the focus group discussions for the ASD CPG implementability concerns (e.g. referral systems, available ASD centers, ASD teams, required qualifications, relevant specific ASD accreditation standards, telehealth, etc.) were repeated for both the ASD CPG Adaptation Group (GAG) and the External Revie Group.

All of the members reviewed the final draft of the Adapted ASD-CPG and consensus was made by an informal voting technique.

Secretarial support was provided by the NCCDD-SHC to organize and facilitate 13 two-hour-meetings for the larger groups that included two face-to-face and 11 online meetings. Moreover, 10 parallel meetings were conducted by subgroups of the GAG to complete specific tasks within the CPGA process (e.g. search for source CPGs, screening source CPGs, quality assessment of CPGs, drafting the first version of the adapted recommendations, identifying and drafting the CPG implementation tools, and drafting the final version of the adapted CPG based on the external review input).

Facilitators and Barriers were discussed and considered twice with the GAG and the ERG and this was reflected on the selection and the final set of implementation strategies and tools.

Appendix Table 4 | Decision Support for the ASD Guideline Adaptation Group

PHASE	MODULE	STEP	TOOL	DECISION		REASON *(if not utilized)*
				Utilized	Not utilized	
ONE: SET-UP	1.1. Preparation	1	1	√		
			2	√		
		2		√		
		3		√		
		4		√		
		5	3	√		
			4	√		
			1	√		
		6	5	√		
TWO: ADAPTATION	2.1. Scope and Purpose	7	6	√		
	2.2. Search and Screen	8	2	√		
			7	√		
		9	8	√		
		10	9		√	Decided to rely on inclusion/ exclusion criteria (filters) & PIPOH compatibility
			10		√	
	2.3. Assessment	11	9	√		
			10	√		
		12	11	√		
		13	12		√	Decided to select the relevant r ecommendations from the four CPGs: ACRC 2018, MOH NZ 2016, NICE 2017, and SIGN-HIS 2016 to the Saudi Arabian Healthcare settings
		14	13		√	Decided to rely on D3 Scores of AGREE II
			14		√	
		15	15		√	Decided to rely on D5, D2 Scores of AGREE II
	2.4. Decision and Selection	16	Appendix Table 4	√		
		17	Decision making and selection	√		The panel modified the options to be two (Accept or Reject) rather than five according to the KSU-Modified-ADAPTE
	2.5. Customization	18	16	√		
THREE: FINALIZATION	3.1. External Review and Acknowledgment Module	19	17	√		
		20		√		
		21		√		
		22		√		
	3.2. Aftercare Planning	23	18	√		
	3.3. Final Production	24		√		

Appendix Table 5 | AGREE Reporting Checklist ASD CPG

CHECKLIST ITEM AND DESCRIPTION	REPORTING CRITERIA	Page #
**DOMAIN 1: SCOPE AND PURPOSE**		
**1. OBJECTIVES:** Report the overall objective(s) of the guideline. The expected health benefits from the guideline are to be specific to the clinical problem or health topic.	☒ Health intent(s) (i.e., prevention, screening, diagnosis, treatment, etc.)<br>☒ Expected benefit(s) or outcome(s)<br>☒ Target(s) (e.g., patient population, society).	10
**2. QUESTIONS:** Report the health question(s) covered by the guideline, particularly for the key recommendations.	☒ Target population<br>☒ Intervention(s) or exposure(s)<br>☐ Comparisons (if appropriate)<br>☒ Outcome(s)<br>☒ Health care setting or context	10, PIPOH model was used (Not PICO) Ref. 14
**3. POPULATION:** Describe the population (i.e., patients, public, etc.) to whom the guideline is meant to apply.	☒ Target population, sex and age<br>☐ Clinical condition (if relevant)<br>☐ Severity/stage of disease (if relevant)<br>☐ Comorbidities (if relevant)<br>☐ Excluded populations (if relevant)	10
**DOMAIN 2: STAKEHOLDER INVOLVEMENT**		
**4. GROUP MEMBERSHIP:** Report all individuals who were involved in the development process. This may include members of the steering group, the research team involved in selecting and reviewing/rating the evidence and individuals involved in formulating the final recommendations.	☒ ame of participant<br>☒ Discipline/content expertise (e.g., neurosurgeon, methodologist)<br>☒ Institution (e.g., St. Peter's hospital)<br>☒ Geographical location (e.g., Seattle, WA)<br>☒ A description of the member's role in the guideline development group	9
**5. TARGET POPULATION PREFERENCES AND VIEWS:** Report how the views and preferences of the target population were sought/considered and what the resulting outcomes were.	☐ Statement of type of strategy used to capture patients'/publics' views and preferences (e.g., participation in the guideline development group, literature review of values and preferences)<br>☐ Methods by which preferences and views were sought (e.g., evidence from literature, surveys, focus groups)<br>☐ Outcomes/information gathered on patient/public information<br>☐ How the information gathered was used to inform the guideline development process and/or formation of the recommendations	NA

Appendix Table 5 | AGREE Reporting Checklist ASD CPG

CHECKLIST ITEM AND DESCRIPTION	REPORTING CRITERIA	Page #
**DOMAIN 2: STAKEHOLDER INVOLVEMENT**		
**6. TARGET USERS:**<br>Report the target (or intended) users of the guideline.	☒ he intended guideline audience (e.g. specialists, family physicians, patients, clinical or institutional leaders/administrators)<br>☒ How the guideline may be used by its target audience (e.g., to inform clinical decisions, to inform policy, to inform standards of care)	10, Ref 14
**DOMAIN 3: RIGOUR OF DEVELOPMENT**		
**7. SEARCH METHODS:**<br>Report details of the strategy used to search for evidence.	☒ Named electronic database(s) or evidence source(s) where the search was performed (e.g., MEDLINE, EMBASE, PsychINFO, CINAHL)<br>☒ Time periods searched (e.g., January 2004 ,1 to March 2008 ,31)<br>☐ Search terms used (e.g., text words, indexing terms, subheadings)<br>☒ Full search strategy included (e.g., possibly located in appendix)	14, Ref 34
**8. EVIDENCE SELECTION CRITERIA:**<br>Report the criteria used to select (i.e., include and exclude) the evidence. Provide rationale, where appropriate.	☒ Target population (patient, public, etc.) characteristics<br>☒ Study design<br>☐ Comparisons (if relevant)<br>☒ Outcomes<br>☒ Language (if relevant)<br>☒ Context (if relevant)	Ref 14, Evidence here refers to Source CPGs
**9. STRENGTHS & LIMITATIONS OF THE EVIDENCE:**<br>Describe the strengths and limitations of the evidence. Consider from the perspective of the individual studies and the body of evidence aggregated across all the studies. Tools exist that can facilitate the reporting of this concept.	☐ Study design(s) included in body of evidence<br>☐ Study methodology limitations (sampling, blinding, allocation concealment, analytical methods)<br>☐ Appropriateness/relevance of primary and secondary outcomes considered<br>☐ Consistency of results across studies<br>☐ Direction of results across studies<br>☐ Magnitude of benefit versus magnitude of harm<br>☐ Applicability to practice context	AGREE II appraisal was done for the Source CPGs. Ref 14

Appendix Table 5 | AGREE Reporting Checklist ASD CPG

CHECKLIST ITEM AND DESCRIPTION	REPORTING CRITERIA	Page #
**DOMAIN 3: RIGOUR OF DEVELOPMENT**		
**10. FORMULATION OF RECOMMENDATIONS:**<br>Describe the methods used to formulate the recommendations and how final decisions were reached. Specify any areas of disagreement and the methods used to resolve them.	☒ Recommendation development process (e.g., steps used in modified Delphi technique, voting procedures that were considered)<br>☒ Outcomes of the recommendation development process (e.g., extent to which consensus was reached using modified Delphi technique, outcome of voting procedures)<br>☒ How the process influenced the recommendations (e.g., results of Delphi technique influence final recommendation, alignment with recommendations and the final vote)	104 ,106
**11. CONSIDERATION OF BENEFITS AND HARMS:**<br>Report the health benefits, side effects, and risks that were considered when formulating the recommendations.	☐ Supporting data and report of benefits<br>Supporting data and report of harms/side effects/risks<br>☐ Reporting of the balance/trade-off between benefits and harms/side effects/risks<br>☒ Recommendations reflect considerations of both benefits and harms/side effects/risks	23
**12. LINK BETWEEN RECOMMENDATIONS AND EVIDENCE:**<br>Describe the explicit link between the recommendations and the evidence on which they are based.	☒ How the guideline development group linked and used the evidence to inform recommendations<br>☒ Link between each recommendation and key evidence (text description and/or reference list)<br>☐ Link between recommendations and evidence summaries and/or evidence tables in the results section of the guideline	92 ,105

Appendix Table 5 | AGREE Reporting Checklist ASD CPG

CHECKLIST ITEM AND DESCRIPTION	REPORTING CRITERIA	Page #
**DOMAIN 3: RIGOUR OF DEVELOPMENT**		
**13. EXTERNAL REVIEW:**<br>Report the methodology used to conduct the external review.	☒ Purpose and intent of the external review (e.g., to improve quality, gather feedback on draft recommendations, assess applicability and feasibility, disseminate evidence)<br>☒ Methods taken to undertake the external review (e.g., rating scale, open-ended questions)<br>☒ Description of the external reviewers (e.g., number, type of reviewers, affiliations)<br>☒ Outcomes/information gathered from the external review (e.g., summary of key findings)<br>☒ How the information gathered was used to inform the guideline development process and/or formation of the recommendations (e.g., guideline panel considered results of review in forming final recommendations)	,13 ,101<br>104-106
**14. UPDATING PROCEDURE:**<br>Describe the procedure for updating the guideline.	☒ A statement that the guideline will be updated<br>☒ Explicit time interval or explicit criteria to guide decisions about when an update will occur<br>☒ Methodology for the updating procedure	89 ,106
**DOMAIN 4 : CLARITY OF PRESENTATION**		
**15. SPECIFIC AND UNAMBIGUOUS RECOMMENDATIONS**<br>Describe which options are appropriate in which situations and in which population groups, as informed by the body of evidence.	☒ A statement of the recommended action.<br>☒ Intent or purpose of the recommended action (e.g., to improve quality of life, to decrease side effects).<br>☒ Relevant population (e.g., patients, public).<br>☒ Caveats or qualifying statements, if relevant (e.g., patients or conditions for whom the recommendations would not apply).<br>☒ If there is uncertainty about the best care option(s), the uncertainty should be stated in the guideline.	22-59

Appendix Table 5 | AGREE Reporting Checklist ASD CPG

CHECKLIST ITEM AND DESCRIPTION	REPORTING CRITERIA	Page #
**DOMAIN 4 : CLARITY OF PRESENTATION**		
**16. MANAGEMENT OPTIONS**<br>Describe the different options for managing the condition or health issue.	☒ Description of management options<br>☒ Population or clinical situation most appropriate to each option	22-59
**17. IDENTIFIABLE KEY RECOMMENDATIONS**<br>Present the key recommendations so that they are easy to identify.	☒ Recommendations in a summarized box, typed in bold, underlined, or presented as flow charts or algorithms.<br>☒ Specific recommendations grouped together in one section.	22 ,59-63
**DOMAIN 5: APPLICABILITY**		
**18. FACILITATORS AND BARRIERS TO APPLICATION**<br>Describe the facilitators and barriers to the guideline's application.	☐ Types of facilitators and barriers that were considered<br>☐ Methods by which information regarding the facilitators and barriers to implementing recommendations were sought (e.g., feedback from key stakeholders, pilot testing of guidelines before widespread implementation)<br>☐ Information/description of the types of facilitators and barriers that emerged from the inquiry (e.g., practitioners have the skills to deliver the recommended care, sufficient equipment is not available to ensure all eligible members of the population receive mammography)<br>☒ How the information influenced the guideline development process and/or formation of the recommendations	106

Appendix Table 5 | AGREE Reporting Checklist ASD CPG

CHECKLIST ITEM AND DESCRIPTION	REPORTING CRITERIA	Page #
**DOMAIN 5: APPLICABILITY**		
**19. IMPLEMENTATION ADVICE/TOOLS**<br>Provide advice and/or tools on how the recommendations can be applied in practice.	☒ Additional materials to support the implementation of the guideline in practice.<br>☐ **For example:**<br>o Guideline summary documents.<br>o Links to check lists, algorithms.<br>o Links to how-to manuals.<br>o Solutions linked to barrier analysis (see Item 18).<br>o Tools to capitalize on guideline facilitators (see Item 18).<br>o Outcome of pilot test and lessons learned.	63-87
**20. RESOURCE IMPLICATIONS**<br>Describe any potential resource implications of applying the recommendations.	☐ Types of cost information that were considered (e.g., economic evaluations, drug acquisition costs)<br>☐ Methods by which the cost information was sought (e.g., a health economist was part of the guideline development panel, use of health technology assessments for specific drugs, etc.)<br>☐ Information/description of the cost information that emerged from the inquiry (e.g., specific drug acquisition costs per treatment course)<br>☐ How the information gathered was used to inform the guideline development process and/or formation of the recommendations	NA
**21. MONITORING/ AUDITING CRITERIA**<br>Provide monitoring and/or auditing criteria to measure the application of guideline recommendations.	☒ Criteria to assess guideline implementation or adherence to recommendations.<br>☒ Criteria for assessing impact of implementing the recommendations.<br>☒ Advice on the frequency and interval of measurement.<br>☒ Operational definitions of how the criteria should be measured.	76

Appendix Table 5 | AGREE Reporting Checklist ASD CPG

CHECKLIST ITEM AND DESCRIPTION	REPORTING CRITERIA	Page #
**DOMAIN 6: EDITORIAL INDEPENDENCE**		
**22. FUNDING BODY**<br>Report the funding body's influence on the content of the guideline.	☒ The name of the funding body or source of funding (or explicit statement of no funding).<br>☒ A statement that the funding body did not influence the content of the guideline.	91
**23. COMPETING INTERESTS**<br>Provide an explicit statement that all group members have declared whether they have any competing interests.	☐ Types of competing interests considered<br>☐ Methods by which potential competing interests were sought.<br>☐ A description of the competing interests. How the competing interests influenced<br>☐ the guideline process and development of recommendations.	101

From:

Brouwers MC, Kerkvliet K, Spithoff K, on behalf of the AGREE Next Steps Consortium. The AGREE Reporting Checklist: a tool to improve reporting of clinical practice guidelines. BMJ 352;2016:i1152. doi: 10.1136/bmj.i1152.

For more information about the AGREE Reporting Checklist, please visit the AGREE Enterprise website at http://www.agreetrust.org.

https://www.agreetrust.org/resource-centre/agree-reporting-checklist/

Appendix Table 6 | The RIGHT-Ad@pt checklist

7 sections, 27 topics, and 34 items		Assessment	Page(s)*	Note(s)
**BASIC INFORMATION**				
**Title/subtitle**				
1	Identify the report as an adaptation of practice guideline(s), that is include "guideline adaptation", "adapting", "adapted guideline/recommendation(s)", or similar terminology in the title/subtitle.	☒ Yes<br>☐ No<br>☐ Unclear	1 ,4 ,9 ,15 ,100	
2	Describe the topic/focus/scope of the adapted guideline.	☒ Yes<br>☐ No<br>☐ Unclear	20	
**Cover/first page**				
3	Report the respective dates of publication and the literature search of the adapted guideline.	☒ Yes<br>☐ No<br>☐ Unclear	Cover page, 102, Reference 20	
4	Describe the developer and country/region of the adapted guideline.	☒ Yes<br>☐ No<br>☐ Unclear	Cover, 15	
**Executive summary/abstract**				
5	Provide a summary of the recommendations contained in the adapted guideline.	☒ Yes<br>☐ No<br>☐ Unclear	24-59 ,64-69	
**Abbreviations and acronyms**				
6	Define key terms and provide a list of abbreviations and acronyms (if applicable).	☒ Yes<br>☐ No<br>☐ Unclear	5	
**Contact information of the guideline adaptation group**				
7	Report the contact information of the developer of the adapted guideline.	☒ Yes<br>☐ No<br>☐ Unclear	4	
**SCOPE**				
**Source guideline(s)**				
8	Report the name and year of publication of the source guideline(s), provide the citation(s), and whether source authors were contacted.	☒ Yes<br>☐ No<br>☐ Unclear	15,93,103,105	

Appendix Table 6 | The RIGHT-Ad@pt checklist

7 sections, 27 topics, and 34 items		Assessment	Page(s)*	Note(s)
**SCOPE**				
**Brief description of the health problem(s)**				
9	Provide the basic epidemiological information about the problem (including the associated burden), health systems relevant issues, and note any relevant differences compared to the source guideline(s).	☒ Yes<br>☐ No<br>☐ Unclear	17 ,21 ,104, 105	
**Aim(s) and specific objectives**				
10	Describe the aim(s) of the adapted guideline and specific objectives, and note any relevant differences compared to the source guideline(s).	☒ Yes<br>☐ No<br>☐ Unclear	21	
**Target population(s)**				
11	Describe the target population(s) and subgroup(s) (if applicable) to which the recommendation(s) is addressed in the adapted guideline, and note any relevant differences compared to the source guideline(s).	☒ Yes<br>☐ No<br>☐ Unclear	21	
**End-users and settings**				
12	Describe the intended target users of the adapted guideline, and note any relevant differences compared to the source guideline(s).	☒ Yes<br>☐ No<br>☐ Unclear	21	
13	Describe the setting(s) for which the adapted guideline is intended, and note any relevant differences compared to the source guideline(s).	☒ Yes<br>☐ No<br>☐ Unclear	21	
**RIGOR OF DEVELOPMENT**				
**Guideline adaptation group**				
14	List all contributors to the guideline adaptation process and describe their selection process and responsibilities.	☒ Yes<br>☐ No<br>☐ Unclear	9	

Appendix Table 6 | The RIGHT-Ad@pt checklist

	7 sections, 27 topics, and 34 items	Assessment	Page(s)*	Note(s)
**RIGOR OF DEVELOPMENT**				
**Adaptation framework/methodology**				
15	Report which framework or methodology was used in the guideline adaptation process.	☒ Yes<br>☐ No<br>☐ Unclear	15 ,97-107	
**Source guideline(s)**				
16	Describe how the specific source guideline(s) was(were) selected.	☒ Yes<br>☐ No<br>☐ Unclear	102	
**Key questions**				
17	State the key questions of the adapted guideline using a structured format, such as PICO (population, intervention, comparator, and outcome), or another format as appropriate.	☒ Yes<br>☐ No<br>☐ Unclear	21	
18	Describe how the key questions were developed/modified, and/or prioritized.	☐ Yes<br>☒ No<br>☐ Unclear	NA	
**Source recommendation(s)**				
19	Describe how the recommendation(s) from the source guideline(s) was(were) assessed with respect to the evidence considered for the different criteria, the judgements and considerations made by the original panel.	☐ Yes<br>☒ No<br>☐ Unclear	NA	
**Evidence synthesis**				
20	Indicate whether the adapted recommendation(s) is/are based on existing evidence from the source guideline(s), and/or additional evidence.	☐ Yes<br>☒ No<br>☐ Unclear	NA	
21	If new research evidence was used, describe how it was identified and assessed.	☐ Yes<br>☒ No<br>☐ Unclear	NA	

Appendix Table 6 | The RIGHT-Ad@pt checklist

7 sections, 27 topics, and 34 items		Assessment	Page(s)*	Note(s)
**RIGOR OF DEVELOPMENT**				
**Assessment of the certainty of the body of evidence and strength of recommendation**				
22	Describe the approach used to assess the certainty/quality of the body/ies of evidence and the strength of recommendations in the adapted guideline and note any differences (if applicable) compared to the source guideline(s).	☐ Yes<br>☒ No<br>☐ Unclear	NA	
**Decision-making processes**				
23	Describe the processes used by the guideline adaptation group to make decisions, particularly the formulation of recommendations.	☒ Yes<br>☐ No<br>☐ Unclear	106	
**RECOMMENDATIONS**				
**Recommendations**				
24	Report recommendations and indicate whether they were adapted, adopted, or de novo.	☒ Yes<br>☐ No<br>☐ Unclear	104 ,106	
25	Indicate the direction and strength of the recommendations and the certainty/quality of the supporting evidence and note any differences compared to the source recommendations(s) (if applicable).	☒ Yes<br>☐ No<br>☐ Unclear	23	
26	Present separate recommendations for important subgroups if the evidence suggests important differences in factors influencing recommendations and note any differences compared to the source recommendations(s) (If applicable).	☒ Yes<br>☐ No<br>☐ Unclear	104 ,106	
**Rationale/explanation for recommendations**				
27	Describe the criteria/factors that were considered to formulate the recommendations or note any relevant differences compared to the source guideline(s) (if applicable).	☒ Yes<br>☐ No<br>☐ Unclear	104 ,106	

Appendix Table 6 | The RIGHT-Ad@pt checklist

7 sections, 27 topics, and 34 items		Assessment	Page(s)*	Note(s)
**EXTERNAL REVIEW AND QUALITY ASSURANCE**				
**External review**				
28	Indicate whether the adapted guideline underwent an independent external review. If yes, describe the process.	☒ Yes<br>☐ No<br>☐ Unclear	13,101,104-106	
**Organizational approval**				
29	Indicate whether the adapted guideline obtained organizational approval. If yes, describe the process.	☒ Yes<br>☐ No<br>☐ Unclear	15	
**FUNDING, DECLARATION, AND MANAGEMENT OF INTEREST**				
**Funding source(s) and funder role(s)**				
30	Report all sources of funding for the adapted guideline and source guideline(s), and the role of the funders.	☒ Yes<br>☐ No<br>☐ Unclear	91	
**Declaration and management of interests**				
31	Report all conflicts of interest of the adapted and the source guideline(s) panels, and how they were evaluated and managed.	☒ Yes<br>☐ No<br>☐ Unclear	101	
**OTHER INFORMATION**				
**Implementation**				
32	Describe the potential barriers and strategies for implementing the recommendations (if applicable).	☒ Yes<br>☐ No<br>☐ Unclear	63	
**Update**				
33	Briefly describe the strategy for updating the adapted guideline (if applicable).	☒ Yes<br>☐ No<br>☐ Unclear	89	
**Limitations and suggestions for further research**				
34	Describe the challenges of the adaptation process, the limitations of the evidence, and provide suggestions for future research.	☒ Yes<br>☐ No<br>☐ Unclear	61	

*page numbers are from the full guideline document

https://www.equator-network.org/reporting-guidelines/a-reporting-tool-for-adapted-guidelines-in-health-care-the-right-adapt-checklist/

المركز الوطني
لاضطرابات النمو الشامل

المجلس الصحي السعودي
Saudi Health Council

www.shc.gov.sa | shc_gov
saudi health council - saudi arabia
